# A Bioorthogonal and Programmable Bacterial Delivery System for Spatiotemporally Targeted Therapy of Solid Tumors

**DOI:** 10.1002/EXP.20240396

**Published:** 2025-12-18

**Authors:** Yu‐Jia Wang, Wen‐Jie Jiang, Hua‐Jun Zhao, Jian‐Qun Deng, Yi‐Min Cai, Yi Li, Xiao‐Lin Meng, Jin Hou, Feng‐Shan Wang, Ju‐Zheng Sheng

**Affiliations:** ^1^ School of Pharmaceutical Sciences Cheeloo College of Medicine Key Laboratory of Chemical Biology of Natural Products (Ministry of Education) Shandong University Jinan China; ^2^ Shandong Provincial Hospital Affiliated to Shandong First Medical University Jinan Shandong China; ^3^ National Glycoengineering Research Center Shandong University Jinan China; ^4^ Institute of Immunopharmaceutical Sciences School of Pharmaceutical Sciences Cheeloo College of Medicine Shandong University Jinan China; ^5^ The State Key Laboratory of Microbial Technology Shandong University Qingdao China

**Keywords:** bioengineered hydrogels, long‐term antitumor protection, spatiotemporal localization of therapeutic bacteria

## Abstract

Rapid advances in synthetic biology are driving the development of microbes as therapeutic agents. While the immunosuppressive tumor microenvironment creates a favorable niche for the systematic delivery of bacteria and therapeutic payloads, these can be harmful if released into healthy tissues. To address this limitation, we designed a spatiotemporal targeting system for engineered *Escherichia coli* Nissle 1917, controlled by azide‐modified hyaluronic acid hydrogel and near‐infrared radiation induction. Using a temperature‐driven genetic status switch, the system produced durable therapeutic output and promoted the therapeutic activity in solid tumors. The combination of azide‐modified hyaluronic acid hydrogel and temperature‐sensitive, engineered *Escherichia coli* Nissle 1917 provided spatiotemporal targeting of solid tumors, not only showing significant therapeutic effects on primary solid tumors, but also inhibiting the metastasis and recurrence of cancer cells by enhancing tumor‐infiltrating lymphocytes. This system has potential for clinical application.

## Graphical abstract

We developed a spatiotemporal targeting oncolytic bacterial therapy system, the STEPT (spatiotemporal targeting EcN‐pfo terminator) system, which injects azide hyaluronic acid hydrogel into tumors to capture DBCO‐labeled engineered *Escherichia coli* Nissle 1917 (EcN). Acidity and temperature regulate bacterial enrichment and payload release, offering a safe and effective cancer combat method.

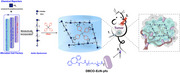

## 1 Introduction

Cell therapy is rapidly becoming an effective class of cancer treatment [1, 2, 3, 4, 5]. Among the candidate cell types for treatment are immune cells [6, 7], which have excelled in treating hematologic malignancies [8, 9, 10]. However, their ability to penetrate and function within the immunosuppressive tumor microenvironment (TME) [11], especially in the immune‐preferred hypoxic core, has hindered their use in solid tumors [12, 13, 14]. Conversely, the reduced immune activity in some tumor cores creates a favorable TME for the growth of certain bacteria [15], which have immunostimulatory effects [16], grow preferentially in hypoxic and immunosuppressive TMEs, and are able to reach tumors via systemic administration [17, 18]. Such tumor‐infiltrating bacteria can be utilized to stimulate antitumor immunity via secretion of payloads that kill tumor cells directly or therapeutically reshape the TME of multiple tumor types [19, 20]. A variety of bacteria have been used in clinical trials as drug delivery systems, including *Lactococcus lactis* [21], *Escherichia coli* Nissle 1917 (EcN) [22, 23, 24, 25, 26], *Listeria monocytogenes* [27, 28], and *Salmonella typhimurium* [29].

EcN is a probiotic that has become the preferred chassis for engineered “smart microbes” used to provide therapeutic effects at disease sites [30]. Its advantages include genetic transmissibility, a proven safety record in humans, and the ability to survive and replicate in the gastrointestinal tract [26] and tumors [31]. EcN strains with therapeutic properties imbued via alteration of their metabolic pathways or introduction of drug delivery systems in situ have shown promise in the treatment of bacterial infections [32], inflammatory bowel disease [33], metabolic disorders [34, 35], and cancer [36]. EcN has been clinically studied as a disease‐targeting carrier in congenital metabolic and cancer diseases [30]. However, as a facultative anaerobic bacterium, EcN exhibits complex tumor‐targeting mechanisms [37]. The disordered vascular system of tumors and their secretion of chemokines hamper the ability of the immune system to clear bacteria in the immunosuppressed TME, allowing preferential growth of bacteria. At the same time, activation of the innate immune response enables such bacteria to further stimulate the immune system by activating various innate immune system signaling pathways [38], including those involving Toll‐like receptors (TLRs) [39], cyclic GMP‐AMP synthase‐stimulator of interferon genes (cGAS‐STING) [40], and NOD‐like receptors (NLRs) [41].

The benefits of microbial tumor therapy are often outweighed by safety concerns regarding the limited control over biological distribution and activity of systemically delivered injections [5]. Specifically, there are documented cases of circulating bacteria colonizing healthy tissues, such as the liver, spleen, and certain hypoxic stem cell niches in vivo. To avoid damaging healthy organs, microbial therapeutics must target tumors specifically.

Bioorthogonal reactions [42], a class of highly efficient and specific chemical reactions that are biocompatible in vivo, may provide solutions to this challenge. The clickable reaction between azide and dibenzocyclooctyne (DBCO) is reportedly one of the most efficient reactions of the bioorthogonal class in vivo [43, 44]. It demonstrated utility in animal model systems, with minimal disturbance to the surrounding biological environment, offering a number of advantages for systematic repeat drug delivery in drug repositories (oral or intravenous). Additionally, hyaluronic acid (HA) hydrogels can be used in spatiotemporal‐positioning strategies. HA is a ubiquitous, non‐sulfated, linear glycosaminoglycan comprising repeats of the disaccharides (β, 1–4)‐gluconic acid (GlcA) and (β, 1–3)‐*N*‐acetylglucosamine (GlcNAc). HA hydrogels have been widely used in tissue engineering, drug storage, cell delivery, and as cell scaffolds for the study of biological processes [45]. However, a safe method for azide‐modified HA (azido‐HA) production is urgently needed. Safe, efficient preparations of clickable HA can be used in hydrogels, which can be crosslinked via bioorthogonal reactions [46]. Therefore, a secure way to produce azido‐HA is urgently needed. Modification of azido‐HA and DBCO with EcN could be used to achieve spatiotemporal tumor targeting. Among the available approaches for regulating microbial function, the use of non‐invasive near‐infrared (NIR) radiation allows temperature‐based transcriptional regulators to be spatiotemporally controlled deeply within tissues. Specifically, the temperature of tumors can be precisely elevated within well‐tolerated ranges, offering great advantages over chemical‐, light‐, and radiation‐based induction methods [47, 48].

Here, we report a combination strategy of spatiotemporal‐targeting with an engineered EcN delivery system that allows the temperature‐sensitive EcN to colonize tumors via intratumor injection of an azido‐HA hydrogel. Briefly, the release of perfringolysin O (pfo, from *Clostridium perfringens*) [49] induced by non‐invasive NIR under acidic tumor conditions initiates a perforating effect on tumor cells, enhancing the spread of tumor antigen release and thereby activating a variety of innate immune signaling pathways, including the TLRs, cGAS‐STING, and NLRs pathways. Simultaneously, the perforating effect has been found to reshape the extracellular TME by disrupting immunosuppression. This enhancement of crosstalk between antigens and dendritic cells (DCs), promotes the infiltration of CD8^+^ T cells and enhances their therapeutic activity. The antitumor effect was offset by antibody‐mediated depletion of CD8^+^ T cells, suggesting that systemic tumor regression was caused by an adaptive immune response. In the cancer models, azido‐HA‐controlled therapeutic microbes were successfully activated by NIR in situ, inducing significant inhibition of tumor growth. Our strategy not only overcomes the immunosuppressive TME but also activates the immune response more fully than the release of tumor antigens mediated by radiotherapy or chemotherapy in immunotherapy of solid tumors. This technology is expected to provide a key tool for the spatiotemporal targeting of potent bacterial therapies in a variety of biological and clinical scenarios.

## 2 Results

### 2.1 Biosynthesis of a Clickable HA Hydrogel Capable of Capturing Engineered Microbes in a Specific Time and Space

**FIGURE 1:**
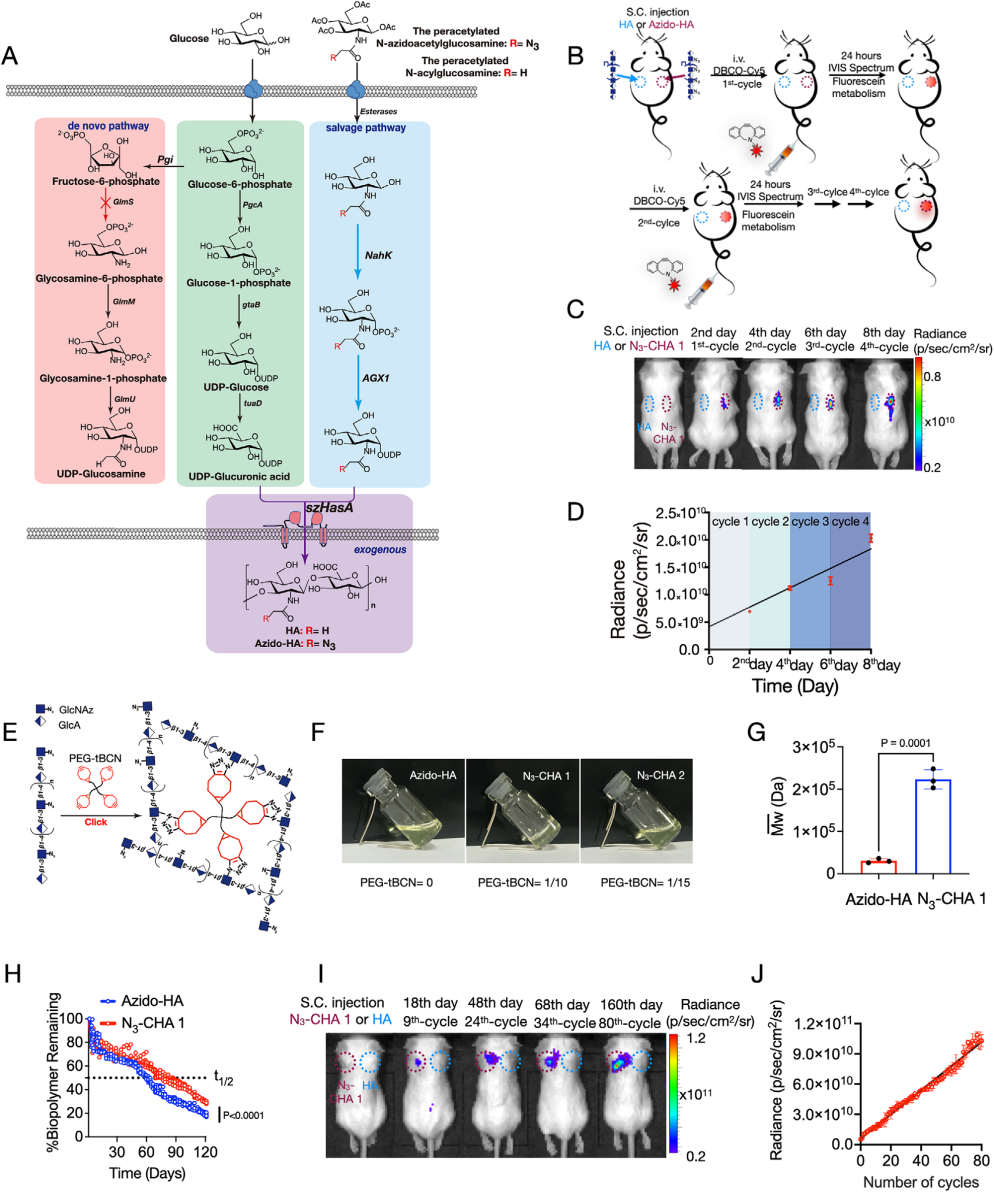
Cell factory secretion of azido‐HA with crosslinking and replenishment ability following fermentation targeting metabolic manufacturing. (A) Extracellular production of azido‐labeled HA via targeted metabolic labeling of capsular polysaccharides in *B. subtilis*. (B–D) Azido‐HA purified from the medium of *B. subtilis* 168SSHA was able to capture circulating bioorthogonal molecules simulated via bioorthogonal reaction, holding them in place for days and exhibiting refilling capability. *n* = 3, biologically independent mice. (B) Schematic representation of bioengineered azido‐HA used as an implantable drug reservoir. IVIS imaging was performed 24 h after each cycle for a total of four cycles. (C) Whole‐body imaging of mice treated with azido‐HA and HA. (D) Fluorescence intensity at the azido‐HA injection site increased approximately linearly with each cycle. Error bars show the SEM of three biologically independent mice. (E–H) Characterization of azido‐HA hydrogel crosslinking through copper‐free click chemistry. (E) Schematic representation of the preparation of azido‐HA crosslinked with PEG‐tBCN to form hydrogels using copper‐free click chemistry. (F) Photos of azido‐HA, N_3_‐CHA 1, and N_3_‐CHA 2. Azido‐HA is normalized to 1. (G) Changes in average molecular weight of azido‐HA before and after crosslinking determined by gel permeation chromatography with multi angle light scattering (GPC‐MALS). Dalton (Da), *n* = 3 biologically independent samples. (H) Time course of the retention percentage of the crosslinked hydrogels and non‐crosslinked hydrogels at the subcutaneous (S.C.) injection site, where *t*_1/2_ was the half‐life of the hydrogels in vivo (see Figure S6). *n* = 7 biologically independent data. (I,J) The increased retention time and biocompatibility of N_3_‐CHA 1 highlighted their potential in an injectable, click‐activated drug delivery system that can be used to recapture DBCO‐labeled molecules. (I) IVIS showing effective, repeated capture of circulating clickable DBCO‐Cy5 molecules at the crosslinked azido‐HA site for 80 cycles (160 days). (J) Linear increase in azido‐HA site fluorescence intensity with increasing cycles; *n* = 3 biologically independent mice. Data in (D,G,H,J) are expressed as the mean ± SEM. *P* values were determined by unpaired two‐tailed Student's *t*‐test (G) and paired two‐tailed Student's *t*‐test (H).

To enable spatiotemporal localization of bacteria, clickable HA was biosynthesized using recombinant *Bacillus* su*btilis* through targeted metabolic labeling [50]. Hyaluronan synthase from *Pasteurella multocida* (pmHAS) was employed for its tolerance to UDP‐*N*‐azidoacetylglucosamine (UDP‐GlcNAz) substrate. However, as a cytoplasmic glycosyl transferase, pmHAS requires additional transporters to export synthesized polysaccharides. Intracellular production of clickable HA by pmHAS in recombinant bacteria makes polysaccharide purification difficult, limiting in vivo application [51]. Therefore, we sought to bioproduce clickable HA as an extracellular polysaccharide using a similar strategy. Notably, the natural bacterial HA polysaccharide is produced by Gram‐negative *P. multocida* Type A and Gram‐positive Groups A and C *Streptococci* [52]. On this basis, we chose the HA synthase from *Streptococcus zooepidemicus* (szHasA) [53], which can directly export HA as a membrane protein, to replace pmHAS as the glycan polymerase in recombinant cells (Figure 1A). So, we designed and constructed recombinant *B. subtilis* strains with the following characteristics [50]: (1) induced expression of exogenous szHasA; (2) deletion of de novo biosynthesis of UDP‐GlcNAc; and (3) constitutive expression of the UDP‐GlcNAz salvage pathway (Figure 1A). The final *B. subtilis* strain (genotype *GlmS*Δ‐*NahK*‐*AGX1*‐*szHasA*‐168) was named *B. subtilis* 168SSHA. Tables S1 and S2 summarize the plasmids, recombinant strains, and primers used in this work.

*B. subtilis* 168SSHA was cultured to produce azido‐HA [50, 54] (Figures S1 and S2A,B). Our data also suggested that UDP‐GlcNAz is a suitable donor substrate for the β1‐3 *N*‐acetylglucosaminyltransferase activity of szHasA. Purified azido‐HA was able to capture circulating bioorthogonal molecules and hold them for weeks, exhibiting refilling capability (Figure 1B–D). Crosslinking azido‐HA with four‐arm polyethylene glycol amine‐tetrad bicyclononyne (PEG‐tBCN) [55] (Figure 1E and Figure S2C) created an injectable N_3_‐CHA 1 hydrogel with optimal viscosity, injectability, and azide group retention (Figure 1F; Figures S2D,E and S3; Tables S3 and S4). Molecular weight (Figure 1G and Figure S4) and structural analyses (Figure S5) confirmed the successful crosslinking. N_3_‐CHA 1 remained longer at the injection site than non‐crosslinked azido‐HA (Figure 1H; Figure S6), without inducing inflammation (Figure S7). This increased retention time and biocompatibility, highlighting the potential of N_3_‐CHA 1 for a click‐activated drug delivery system that can recapture bacteria multiple times (Figure 1I,J; Figure S8). Overall, crosslinked azido‐HA hydrogels warrant further research as an innovative platform for sustained, refillable drug delivery.

### 2.2 Bioorthogonal Strategy Enables Advantageous, Regulated, Spatiotemporal Localization of Bacteria in Tumor

**FIGURE 2:**
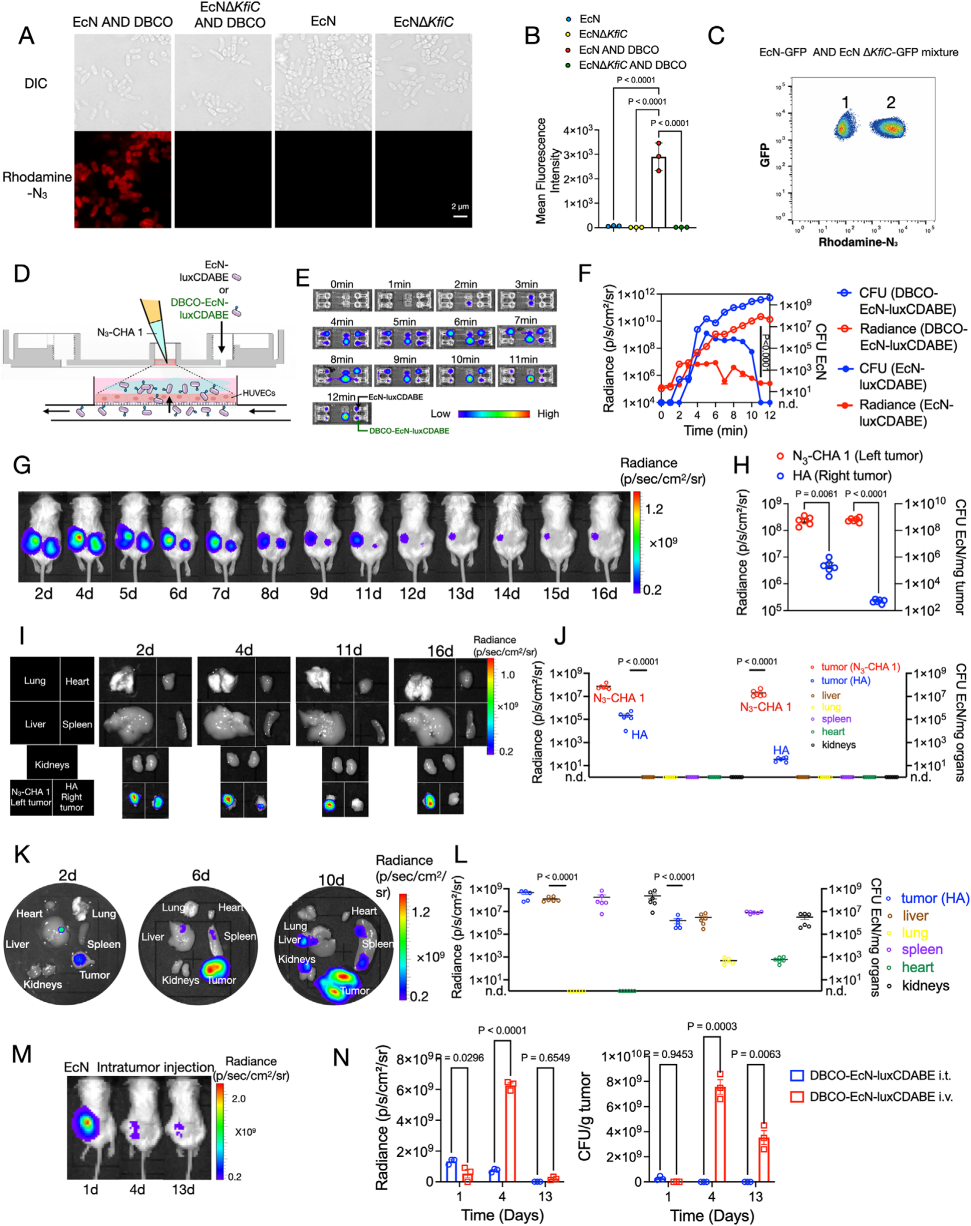
Bioorthogonal strategy for spatiotemporal localization of bacteria in tumor. (A–C) Fluorescein rhodamine‐N_3_ labeling of DBCO on the capsule polysaccharide heparosan of EcN. DIC, Differential interference contrast. The results of structured illumination microscopy (SIM) (A) and flow cytometry (B) showed that DBCO was effectively labeled by EcN only when heparosan was present; Scale bar, 2 µm; *n* = 3 biologically independent samples. (C) Flow cytometry analysis of labeled EcN‐GFP mixed with EcN∆*KfiC*‐GFP in equal proportion, revealing two clusters, in which EcN‐GFP cells and EcN∆*KfiC*‐GFP cells were positive and negative for rhodamine‐N_3_, respectively. (D–F) Simulation of the vascular environment at the tumor site using an extracorporeal circulation device featuring a microfluidic chip. (D) DBCO‐EcN‐luxCDABE, which was used to simulate spatiotemporal localization in vitro, was shown to cross blood vessels. (E) IVIS measurement of the distribution of EcN‐luxCDABE, with or without DBCO modification, at 12 min of circulation via pumping into two chambers of HUVECs pre‐cultured to simulate vascular epithelium, in the presence of N_3_‐CHA 1. (F) The bioluminescence intensity and EcN‐luxCDABE CFU values of the two culture chambers were calculated at the indicated time points; *n* = 3 biologically independent data. (G–J) N_3_‐CHA 1 as the basis of spatiotemporal localization of bacteria in vivo. (G) Bilateral fibrosarcoma model: the left tumor was injected with N_3_‐CHA 1, and the right tumor was injected with ordinary HA. After 1 h, DBCO‐EcN‐luxCDABE (5 × 10^6^ CFU, 100 µL) was injected intravenously for in vivo imaging at the specified time points. *n* = 6, biologically independent mice. (H) EcN load and CFU statistics at day 11. (I) Tumors and organs were dissected for individual imaging. (J) Imaging and CFU analysis at day 20 in other organs and tumors. (K) Mouse model of fibrosarcoma with intratumor injection of ordinary HA and 1 h later intravenous injection of EcN‐luxCDABE (5 × 10^6^ CFU, 100 µL). Bioluminescence imaging of the tumors and organs dissected from the above, showing the distribution of EcN‐luxCDABE colonization on day 2, 6, and 10. (L) Bioluminescent IVIS signals and CFU represent the distribution of EcN‐luxCDABE at day 20 in vivo. *n* = 6, biologically independent mice. (M,N) Comparison between direct intratumor (i.t.) and intravenous (i.v.) injection of DBCO‐EcN‐luxCDABE in a unilateral tumor model. (M) Bioluminescence imaging showing the distribution of EcN‐luxCDABE (i.t.). (N) EcN‐luxCDABE tumor loads and CFUs in the two groups on days 1, 4, and 13; *n* = 3 biologically independent mice. Data in B, F, H, J, L, and N are expressed as the mean ± SEM. *P* values were determined by unpaired, two‐tailed Mann–Whitney test (H, J, L, N); non‐parametric one‐way ANOVA with Dunn's multiple comparisons test (B); two‐way ANOVA and Tukey's multiple comparisons test (F).

To achieve spatiotemporal localization of bacterial therapy, we utilized the principle of bioorthogonal reactions to accurately capture EcN in tumors. Specifically, we achieved effective DBCO‐labeling of EcN in vitro (Figure 2A–C; Figure S9A–D). This labeling method enables full DBCO‐labeling of different batches of EcN (Figure S9E) that is maintained for at least 100 min (Figure S9F), ensuring the uniformity and stability of labeling (Figure S9G). To visualize the biological distribution of bacteria, the bioluminescent EcN‐luxCDABE strain was designed and constructed [20, 31, 56, 57, 58]. Microfluidic simulation assays showed that, with the assistance of N_3_‐CHA 1 hydrogel, DBCO‐labeled EcN‐luxCDABE crossed blood vessels and remained on the side of the upper culture chamber containing azido‐HA hydrogels, whereas unlabeled EcN‐luxCDABE could not colonize the upper chamber (Figure 2D–F; Figure S10). At 12 min, the bioluminescence intensity of DBCO‐EcN‐luxCDABE was significantly higher than that of the unlabeled EcN‐luxCDABE (Figure 2E). Due to the increase in luminescent area of the EcN‐luxCDABE group at the seventh minute, its average bioluminescent intensity suddenly drops, without synchronizing with the decrease in CFU of the EcN‐luxCDABE group (Figure 2F). Simultaneously, in a bilateral tumor model, DBCO‐labeled EcN‐luxCDABE was found to be distributed in both tumors at the beginning because of the hypoxic and immunosuppressive properties of the tumors (Figure 2G). However, DBCO‐EcN‐luxCDABE was specifically enriched for an extended duration in tumors injected with N_3_‐CHA 1 compared with that in tumors injected with regular HA (Figure 2G,H). Notably, during 8 days of treatment, measurements of the dynamic changes of DBCO tags on EcN in tumors revealed that, although the fluorescence intensity gradually decreased, the number of clickable bacteria increased (Figure S11), indicating that some DBCO modifications were still retained on the surface of progeny EcN. Due to the natural tumor tropism of EcN and the ability of azido‐HA hydrogels to capture DBCO‐EcN at the tumor site, in vivo targeting experiments confirmed that the biological distribution of EcN only occurred at the N_3_‐CHA 1 tumor site (Figure 2I,J), with no misdistribution of the bacteria in other organs, as compared with the findings in HA‐injected tumors (Figure 2K,L). We also investigated the effect of direct intratumor injection of DBCO‐EcN‐luxCDABE after intratumor injection of N_3_‐CHA 1 and found that the bacteria did not achieve long‐term colonization of the tumor compared with that following intravenous injection (Figure 2M,N; Figure S14). Furthermore, our findings indicated that the DBCO molecule was attached to the capsular polysaccharide heparosan of EcN (Figure 2A–C). DBCO was not effectively labeled on EcN∆*KfiC* [36], demonstrating that the presence of heparosan is critical for the spatiotemporal localization, similar to azido‐HA.

**FIGURE 3:**
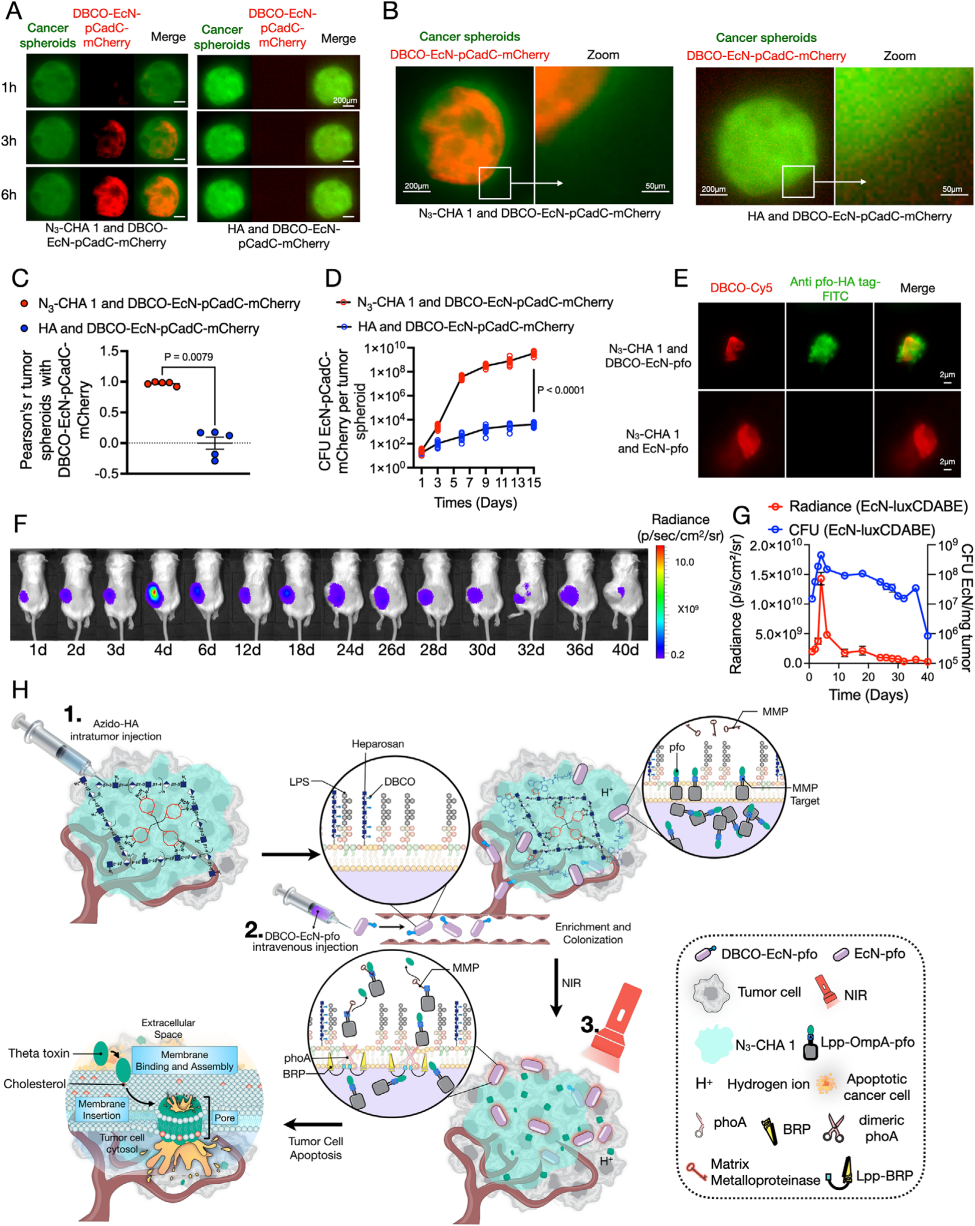
N_3_‐CHA 1 hydrogel enriches and promotes colonization of engineered DBCO‐bacteria in tumor. (A–D) Dynamic observation of changes in MCF7‐GFP containing N_3_‐CHA 1 (or HA)‐derived tumor spheroids within 6 h of the addition of DBCO‐EcN‐pCadC‐mCherry to the medium. (A) Compared with the HA group, N_3_‐CHA 1 achieved co‐localization of tumor spheroids with DBCO‐EcN‐pCadC‐mCherry under acidic conditions (left). With prolonged coculture time, EcN‐pCadC‐mCherry achieved long‐term colonization in the inner core of N_3_‐CHA 1 tumor spheroids; *n* = 5, biologically independent data. Scale bar, 200 µm. (B) The zoom images directly show the distribution of EcN‐pCadC‐mCherry outside the tumor sphere in (A). Scale bar, 200 µm. (C) Fluorescence microscopy of tumor spheroids and EcN‐pCadC‐mCherry at 6 h. At least 30 tumor spheroids were quantified per donor (*n* = 5 donors). (D) The number of CFU of EcN‐pCadC‐mCherry in tumor spheres was quantified within 15 days. (E) Bioorthogonal reaction of azido‐HA with DBCO‐EcN‐pfo demonstrated at the cellular level using the transwell device (see Figure S20A). Scale bar, 2 µm. *n* = 10 biologically independent fields of view. (F,G) Colonization of EcN‐luxCDABE after spatiotemporal localization in a unilateral tumor model in mice in vivo. Bioluminescence imaging (F) and changes (G) in the tumor load and CFU of EcN‐luxCDABE, which peaked on the fourth day after EcN injection; *n* = 3 biologically independent mice. (H) Spatiotemporal targeting EcN‐pfo terminator (STEPT) strategy for the enrichment and colonization of DBCO‐EcN‐pfo in blood circulation. We established the STEPT strategy for the treatment of solid tumors: intratumor injection of azido‐HA hydrogel (1); intravenous injection of DBCO‐EcN‐pfo (2); mild heating induced by non‐invasive NIR treatment at the tumor site to activate EcN, which releases the effective payload, pfo (Perfringolysin O, a water‐soluble monomeric cytolysin secreted by pathogenic *Clostridium perfringens*, oligomerizes and forms large pores upon encountering cholesterol‐containing membranes. Free pfo binds to cholesterol on the membrane of tumor cells, causing pfo monomers to aggregate into polymers, which creates a pore‐forming effect that directly leads to tumor cell death.), thereby initiating an antitumor perforation effect on the cell membrane of targeted tumor cells. Data in (C,D,G) are expressed as the mean ± SEM. *P* values were determined by unpaired two‐tailed Student's *t*‐test (C); two‐way ANOVA and Sidak's multiple comparisons test (D).

To test the regulation of the EcN response within tumors, we first assessed the acidic environment of the tumor as a condition for altered expression of a reporter gene. The EcN‐pCadC‐mCherry strain was engineered to carry the red fluorescent mCherry reporter under the control of promoter pCadC, which is regulated by a membrane‐tethered activator protein (CadC) that exhibits increased activity in medium with an acidic pH compared with a neutral pH [58]. Using the cancer spheroids model [58], we discovered that EcN‐pCadC‐mCherry not only induced the expression of mCherry in tumors but also effectively penetrated the core region of solid tumors containing N_3_‐CHA 1 hydrogel (Figure 3A–C). Simulation of spatiotemporal localization of DBCO‐EcN‐pCadC‐mCherry by using tumor spheroids, showing its ability to penetrate the inner core of tumors, reaching the N_3_‐CHA 1 hydrogel, and proliferating under tumor conditions. This indicated the co‐localization of tumor spheroids with EcN under acidic conditions (Figure 3C,D). Then, we observed that N_3_‐CHA 1 hydrogels can use their advantages to attract engineered bacteria (Figure 3E). We also evaluated the pharmacokinetics of EcN (Figure 3F,G). After intratumor injection of N_3_‐CHA 1 hydrogel and intravenous injection of DBCO‐EcN‐luxCDABE, the number of EcN‐luxCDABE cells in tumors reached a peak on day 4 (Figure 3F), gradually decreasing thereafter with metabolic clearance (Figure 3G) and without off‐target effects (Figure S12), importantly demonstrating the biosafety of the bacterial therapy. Furthermore, when the EcN injection was repeated on day 7, the number of bacteria in the tumor peaked again on day 13 (Figure S13).

### 2.3 Spatiotemporal Targeting EcN‐pfo Terminator (STEPT)‐Mediated Release of Spatiotemporally Regulatable Payloads Shows Strong Therapeutic Potential

After achieving tumor‐targeted bacterial therapy, our next step was to use EcN as a carrier of controlled‐release active substances. Therefore, we modified the STEPT system to enable controlled release of active antitumor payloads in solid tumors by employing synthetic biology tools to precisely adjust the interactions between EcN and the environment, as shown in Figure 3H and Figure S15.

We designed genetic circuits that sense and respond to changes in the acidic environment and local warming (by NIR) of tumors, thereby autonomously regulating the release of the bacterial payload. This improved the safety and effectiveness of STEPT‐mediated bacterial cancer treatment against solid tumors. The final EcN strain, which was named EcN‐pfo (Figure S15), secreted the cytotoxic molecule pfo from *C. perfringens* [49]. The expression of pfo can be controlled more precisely using inducible promoters that help to avoid toxicity to normal tissues. As shown in Figure S15, EcN‐pfo induced the pCadC‐driven expression of pfo precursor proteins carrying matrix metalloproteinase (MMP) cleavage sites under acidic conditions (pH = 6.8) in the tumor. These bacteria that were located in the tumor began to express the pfo precursor protein, which was distributed in the periplasmic space and outer membrane. On day 4 post‐injection, NIR was applied to the tumor, raising the local tumor temperature to 42°C [59] and thereby triggering expression of temperature‐sensitive phosphatase (phoA) and bactericin‐releasing protein (BRP) [60]. As proteins associated with the release of bacteriocins [61, 62], phoA and BRP increase the permeability of the bacterial outer membrane and promote the release of pfo precursors into the TME, where they are cleaved by MMP to release pfo [63]. Monomer pfo binds to cholesterol on the tumor cell membrane, aggregating into polymers with a perforating effect [49] that directly leads to tumor cell death (Figure 3H).

**FIGURE 4:**
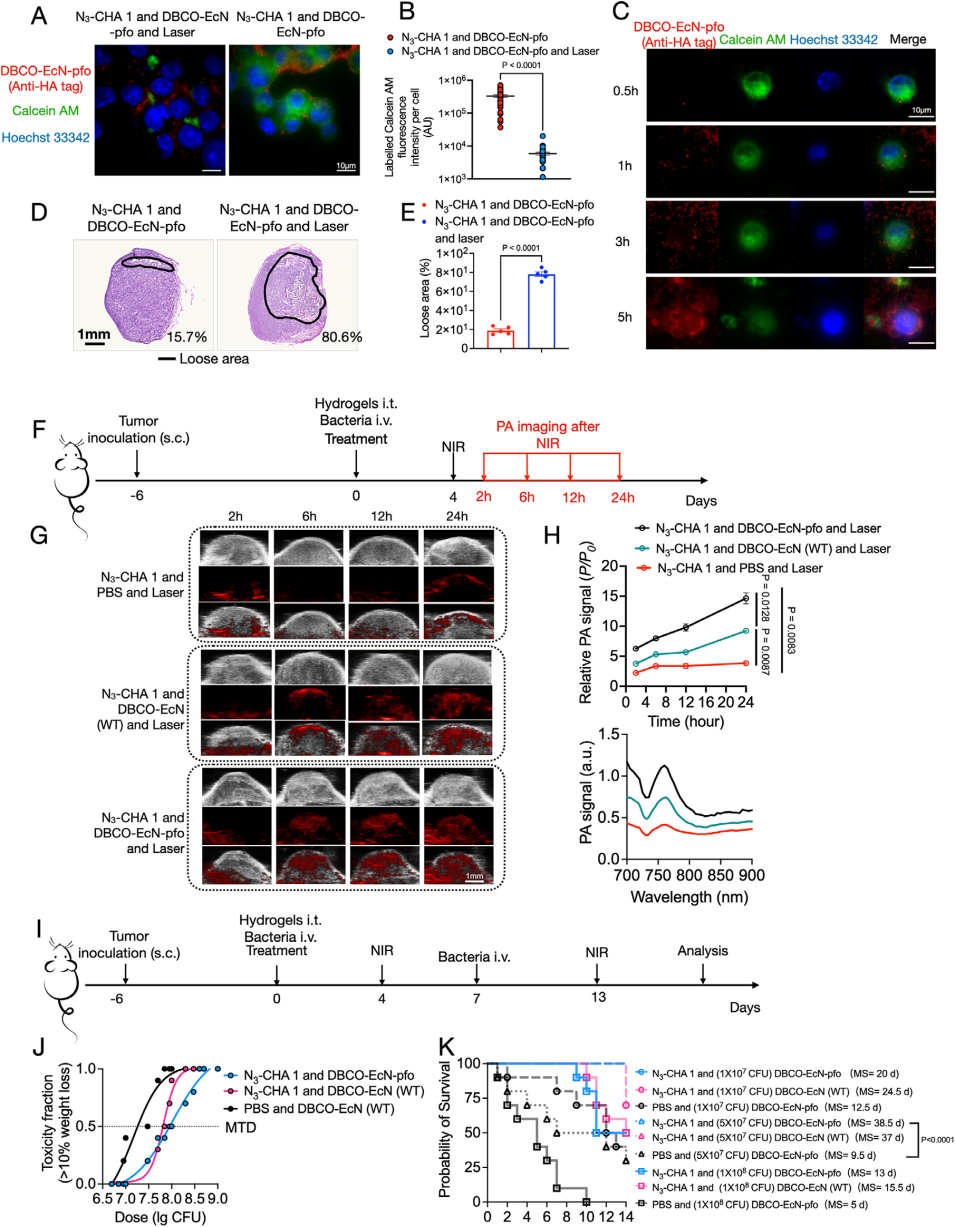
Use of EcN to release pfo in the STEPT strategy for tumor treatment. (A,B) Tumor killing effect of pfo demonstrated at the cellular level. Anti‐HA tag‐tetramethylrhodamine (TRITC) antibody‐labeled EcN‐pfo (with HA tag). Calcein AM labeling of MCF7 tumor cells showed weak fluorescence intensity after laser (NIR); Scale bar, 10 µm. AU, arbitrary units. *n* = 30 biologically independent fields of view. (C) Dynamic observation of the entire process of tumor cell killing in the STEPT system using the transwell device (see Figure S20C); Scale bar, 10 µm. (D,E) H&E staining of tumor sections (D) and analysis of tumor tissue porosity (E) from 4T1‐luc orthotopic breast cancer tumor mice, with and without NIR treatment. (F) Timeline of treatment and PA imaging of fibrosarcoma model mice. (G) PA imaging performed in different groups of mouse tumors at different time points. Scale bar, 1 mm. (H) Comparison of relative PA signals of different groups within 24 h (up panel). PA signals of full wavelength scans varied between groups, with the strongest signal at 760 nm, which is the absorption signal of hemoglobin at the tumor site (bottom panel); *n* = 3 biologically independent mice. (I) Timeline of treatment and survival analysis of the fibrosarcoma model. Weight changes on day 15 (J) and survival times of mice in different groups were measured (K). *n* = 10 biologically independent mice. Data in (B,E,H,J,K) expressed as the mean ± SEM. *P* values determined by unpaired two‐tailed Student's *t*‐test (B,E); two‐way ANOVA with Bonferroni post‐hoc test (H); and log‐rank (Mantel–Cox) test for survival curves (K).

Together with NIR irradiation to accurately control the temperature at the tumor site, we engineered a temperature‐sensitive [59] strain with mCherry reporter expression (EcN‐pR‐pL‐mCherry) to directly reflect the temperature response of the bacteria within the tumor (Figures S16 and S17). The temperature‐sensitive promoter is extremely sensitive to temperature and will only activate the system when the temperature reaches 42°C. The fever temperature of a patient will not reach 42°C, hence it will not trigger the system. The optimal NIR condition is three rounds of periodic irradiation with 0.308 W/cm^2^ at 5 cm for 5 min ON and 5 min OFF (Figure S16). We name the bacteria‐hydrogel combination: Spatiotemporal targeting EcN‐pfo terminator (STEPT). The STEPT system had strong pfo secretion and expression ability (Figures S18 and S19), as well as tumor‐killing ability (Figure 4A–C; Figures S20 and S21). Additionally, we observed that the STEPT system degraded the physical barrier of tumors to a certain extent (Figure 4D,E; Figure S22) and enhanced the photoacoustic signal (Figure 4F–H). These observations indicated that the bacteria exerted a destructive effect by accumulating near the tumor blood vessels, resulting in tumor thrombosis and increased photothermal absorption. Next, we compared intravenously (i.v.) and intratumorally (i.t.) injected bacteria for intratumor distribution of bacteria [64]. With i.t. injection, the number of bacteria increased rapidly in a short time (Figure 2M), the colonization ability was weak (Figure 2N), and the bacteria were mainly distributed in the necrotic area of the distal tumor blood vessels (Figure S23). In contrast, i.v. injected bacteria were distributed in both proximal and distal tumor blood vessels (Figure S23). Bacteria mainly accumulated near blood vessels in tumors, with bacteria‐secreted pfo quickly killing tumors and inducing the destruction of tumor blood vessels, leading to tumor thrombosis [65]. Subsequently, the bacteria exhibited long‐term expansion of colonization (Figure S24). To better visualize the distribution of bacteria within the tumor, we plan to characterize this distribution at the single‐cell level in the future [66]. We will use the invasion‐adhesion‐directed expression sequencing (INVADEseq) approach at the eukaryotic single‐cell level resolution, utilizing the 10× barcode to identify single cells with intracellular bacteria. Notably, the presence of N_3_‐CHA 1 helped to increase the tolerable dose of EcN‐pfo, improving the safety and efficacy of the STEPT system (Figure 4I–K).

### 2.4 Antitumor Efficacy and Safety of STEPT in Tumor Models

**FIGURE 5:**
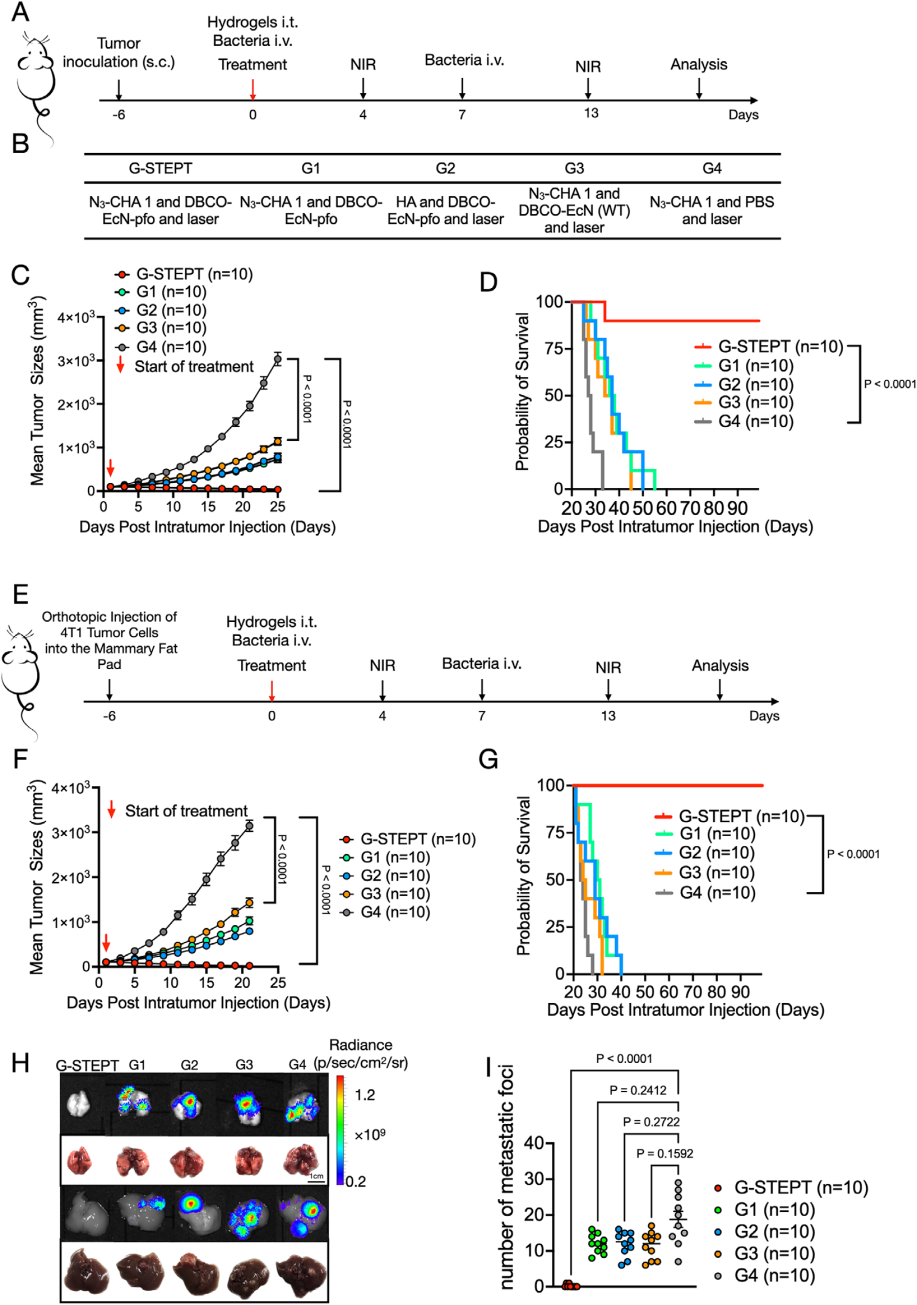
Effect of STEPT on S.C. fibrosarcoma and orthogonal breast cancer with high rates of metastasis in mice. (A–D) Effect of STEPT on S.C. fibrosarcoma in mice. (A) Timeline of STEPT treatment of fibrosarcoma‐bearing model mice. (B) Grouping of treatments. (C) Tumor growth curves after the indicated treatments as recorded shown in Figure S25A; *n* = 10 biologically independent mice. (D) Survival curves over a total monitoring time of 100 days, showing a median survival of > 90 days in the G‐STEPT group, and death within 50 days in the other groups; *n* = 10 biologically independent mice. (E–I) Efficacy of STEPT in treatment of orthogonal breast cancer and prevention of metastasis. (E) Timeline of STEPT treatment of mammary tumor‐bearing model mice established by orthotopic injection of luciferase‐expressing 4T1 (4T1‐luc) cells. Grouping of treatments is the same as (B). (F) 4T1‐luc tumor growth curves showed the G‐STEPT exhibited the strongest inhibition of breast cancer tumor growth; *n* = 10 biologically independent mice. See Figure S25D for IVIS images. (G) Survival curves over a total monitoring time of 100 days, showing a median survival of > 90 days in the G‐STEPT group, and death within 50 days in the other groups; *n* = 10 biologically independent mice. (H,I) Representative bioluminescence images and photographs (H), and corresponding quantification of lung and liver metastatic nodules, after the indicated treatments (I); *n* = 10 biologically independent mice. Data in (C,D,F,G,I) expressed as the mean ± SEM. *P* values determined by two‐way ANOVA with Bonferroni post‐hoc test (C,F), log‐rank (Mantel–Cox) test for survival curves (D,G) and one‐way ANOVA with Tukey's post‐hoc test (I).

The effect of the STEPT system against fibrosarcoma growth was investigated using the WEHI164 tumor mouse model and following the treatment schedule outlined in Figure 5A. Mice were randomly divided into five groups (Figure 5B), including G‐STEPT (according to Figure 5A, intratumor injection of N_3_‐CHA 1, intravenous injection of DBCO‐EcN‐pfo, followed by near‐infrared irradiation), G1 (intratumor injection of N_3_‐CHA 1, intravenous injection of DBCO‐EcN‐pfo), G2 (intratumor injection of HA, intravenous injection of DBCO‐EcN‐pfo, followed by near‐infrared irradiation), G3 (intratumor injection of N_3_‐CHA 1, intravenous injection of DBCO‐EcN, followed by near‐infrared irradiation), G4 (intratumor injection of N_3_‐CHA 1, intravenous injection of PBS, followed by near‐infrared irradiation). As shown in Figure 5C, each group had a similar tumor volume on the first day. However, the G‐STEPT group exhibited the strongest inhibition of tumor growth, attacked tumor cells indiscriminately, and potently inhibited tumor growth as exhibited by tumor shrinkage, blackening, and crusting (Figure S25A), with significant reductions in average tumor volume and mass, and the longest survival time (Figure 5D and Figure S25BC). In the other treatment groups, tumor volumes exceeded 500 cm^3^ (Figure 5C). There were only mild antitumor effects in the G1 and the G2 groups, which may be attributable to the spontaneous distribution of a small amount of EcN‐pfo to tumor tissues. In addition, we found that NIR alone had no significant effects on tumor cells in the TME in this study (Figure S26).

The excellent effect of the STEPT system against fibrosarcoma encouraged us to further investigate its antitumor effects in a metastatic breast cancer tumor model. As expected, the STEPT system effectively inhibited the development of breast cancer (Figure 5E–G; Figure S25D–F) and controlled tumor metastasis (Figure 5H,I). Notably, STEPT treatment effectively inhibited lung and liver metastasis of tumors, which in turn helped to prolong survival (Figure 5G) and achieved a complete response (Figure S25F). Because EcN‐pfo in the STEPT system only colonized tumor sites (Figure S27), these bacteria may have had an effect on proto‐ecological microorganisms in tumor cells (Figure S28). It is possible that our STEPT therapy causes the death and extreme decline of tumor‐native microorganisms that mediate tumor metastasis, changing the microbial niche within the tumor [67].

In addition, the STEPT system exhibited some effectiveness for large tumors (Figure S29), and could be used in combination with cancer vaccines, adoptive T‐cell transfer, and chimeric antigen receptor T‐cell therapy in the future.

We also assessed the safety of the STEPT system, finding that, although tumor mass did not correlate linearly with the colonization of EcN‐pfo (Figure S30A), N_3_‐CHA 1 significantly increased the abundance of EcN‐pfo at tumor sites (Figure S24). Intravenous or oral cefixime administered simultaneously with EcN‐pfo in vivo completely eliminated these bacteria within 2 or 4 days (Figure S30B,C). After STEPT treatment, there was little change in body temperature and weight compared with the healthy mice (Figure S31A). Physiological and biochemical indexes were also normal after this STEPT treatment, with the exception of slight fluctuations in platelet count (Figures S31B and S32A). We also ruled out the possibility of heparin‐induced thrombocytopenia [4] (Figure S32B), and determined that there were no significant changes in mean platelet volume (MPV) or platelet aggregation (Figure S33). Overall, our STEPT system was found to be safe.

### 2.5 Antigens Produced by STEPT Spread and Activate CD8^+^ T Cells via DC Delivery, Leading to the Mediation of Antitumor Immunity

Investigation of the effects of STEPT treatment on the immune system has become our next reasonable goal. Compared with the other groups, the G‐STEPT showed increased proportions and numbers of CD8^+^ T cells, with little effect on CD4^+^ T cells in the breast tumor‐bearing model mice at 14 days (Figures S34A and S35A). CD8^+^ T cells that produced interferon γ (IFN‐γ) and tumor necrosis factor α (TNF‐α) were also expanded (Figures S34B,C and S35B,C), while CD4^+^ T cells had little change (Figures S34D and S35B). We then evaluated the systemic immunoregulatory consequences of STEPT treatment throughout tumor development by analyzing immune responses at early (day 6), intermediate (day 15), and advanced (day 24) time points using flow cytometry (Figure S36A). We found that STEPT treatment induced an antitumorigenic, immune‐stimulating environment in the TME, as characterized by expansion of IFN‐γ‐producing CD4^+^ T helper cell 1 (Th_1_) on the sixth day and CD8^+^ type 1 cytotoxic T (Tc_1_) cells at all‐time points evaluated (Figure S36B,C). These CD8^+^ T cells actively proliferated, as demonstrated by upregulated Ki67 expression (Figure S36D,E). The inhibitory immune checkpoint molecules programmed death 1 (PD‐1) and T cell immunoglobulin and mucin domain–containing molecule 3 (TIM‐3) were not affected by this STEPT protocol (Figure S36F,G), suggesting that it selectively mediated T effector cell responses in the TME.

Although treatment with G‐STEPT affected the frequency and/or absolute number of certain innate immune cell types (Figures S37 and Figure S38), these changes were inconsistent. However, infiltration of antigen‐presenting DCs in the tumor was significantly upregulated (Figure S39). Additionally, G‐STEPT treatment significantly triggered more antigen‐specific DCs (CD45^+^ CD11c^+^ OVA^+^) and activated DCs (CD11c^+^ CD80^+^ CD86^+^) in the lymph nodes (Figure S39), suggesting that DCs mature in the lymph nodes through STEPT treatment [68]. We also found that NIR alone had no significant effect on immune cells in the TME (Figure S40).

Due to the nature of bacterial activation of the innate immune response, we used inhibitors to investigate the signaling pathways triggered by the STEPT system, which included the TLRs, cGAS‐STING, NOD1, and NLRP3 pathways, and their possible roles in STEPT‐induced DC maturation. As shown in Figure S41, treatment with TLR2 inhibitor C29, TLR4 inhibitor resatorvid, cGAS‐STING inhibitor H‐151, TLR7/9 inhibitor hydroxychloroquine sulfate, NOD1 inhibitor NOD‐IN‐1, or NLRP3 inhibitor CY‐09 alone partially inhibited STEPT‐induced maturation of bone marrow‐derived DCs (BMDCs). Treatment with the TLR5 inhibitor TH‐1020 had no significant effect. These findings suggest that STEPT stimulates BMDCs by activating the TLR2, TLR4, TLR7/9, cGAS‐STING, NOD1, and NLRP3 pathways (Figure S41). In the future, we will provide a more detailed discussion on the signaling pathways activated by the bacterial delivery system and the specific roles of different inhibitors in modulating the immune response.

**FIGURE 6:**
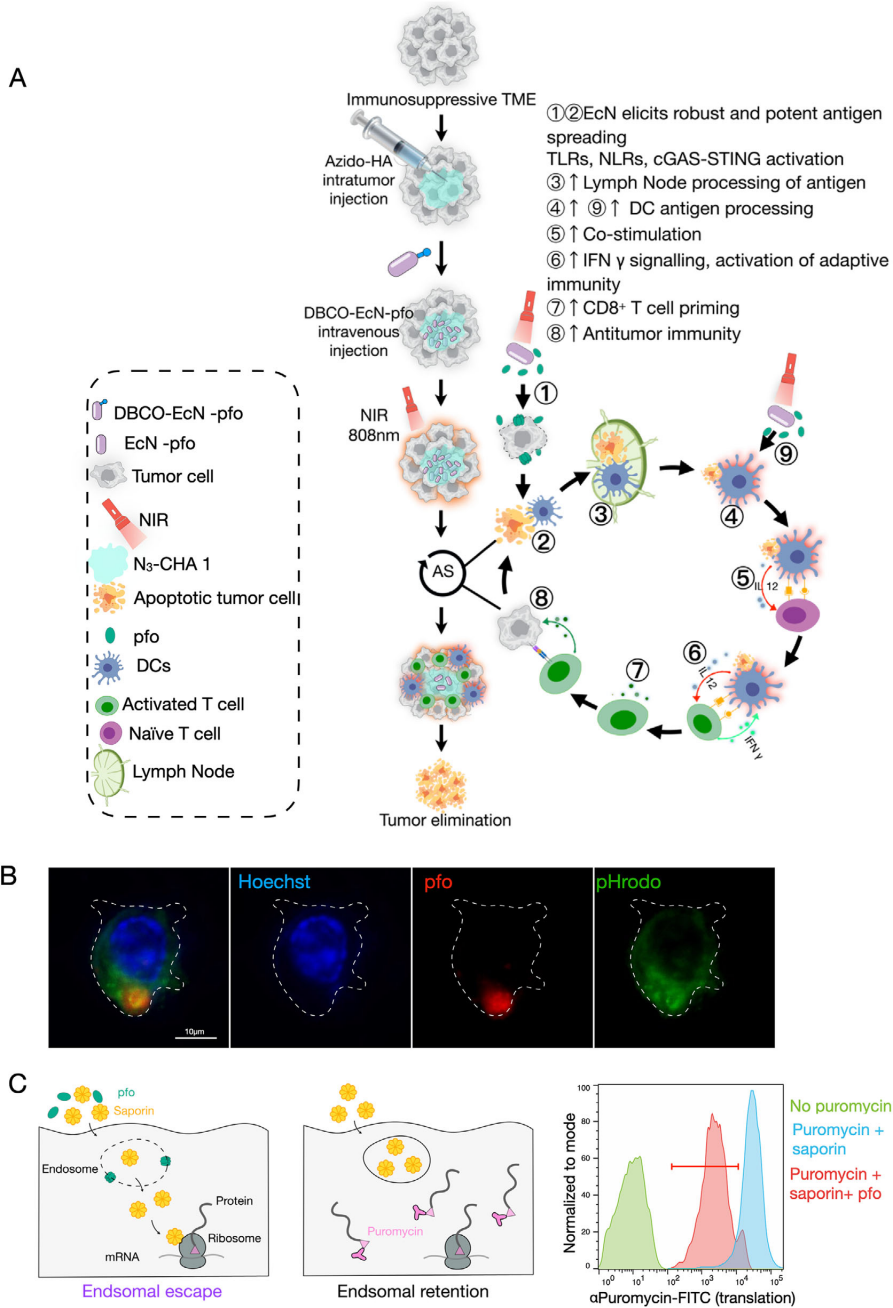
Immune system response induced by STEPT treatment. (A) Immunological mechanism of STEPT system. (B) SIM images of BMDCs in STEPT treatment. pHrodo (green), Hoechst (blue), and pfo (red). Data represent two independent experiments, each with at least 80 cells. (C) Schematic illustration of pfo‐induced endosomal escape (left panels) in BMDCs. To monitor the endocytic escape induced by pfo, we used saporin with a shorter incubation time and lower concentration. We labeled intracellular puromycylated polypeptides with a fluorescent fluorescein isothiocyanate (FITC) antibody, allowing for a flow cytometry‐based readout of translation efficiency, thereby indicating endocytic escape. We developed the saporin‐puromycin assay using BMDCs. (See Figure S20E for details.) If saporin is retained within endosomes, translation remains high; if it escapes into the cytosol, it inhibits ribosomes, inducing translation arrest. Cells in translation arrest are denoted by the red gate. With the help of pfo, a low concentration of saporin is quickly released from the cytoplasm in a short time, indicating that endocytic escape occurs.

Furthermore, depletion studies conducted in vivo showed that CD8^+^ T cells (Figure S43) were critical for STEPT treatment‐mediated tumor suppression, unlike CD4^+^ T cells (Figure S42). Notably, studies have demonstrated that the regulation of matrix viscoelasticity can induce functional differences in T cells, with culturing in slow‐relaxation matrix showing the highest lethality [69]. Therefore, we investigated the possibility that N_3_‐CHA 1 hydrogel in the tumor could alter T‐cell activity. It is known that upregulation of the activated protein 1 (AP‐1) family transcription factor c‐Jun is associated with T‐cell activation [70]. We found that c‐Jun was overexpressed in CD8^+^ T cells following incubation with crosslinked hydrogel (Figure S44A to C), indicating that crosslinked azido‐HA may have potential for saving depleted T cells and enhancing T‐cell activity [69, 70]. Next, we evaluated the relationship between tumor burden and CD8^+^ T cells after G‐STEPT treatment. An increased frequency of tumor‐infiltrating IFN‐γ^+^ CD8^+^ T cells was strongly associated with reduced tumor burden in mice treated with G‐STEPT (Figure S44D), but not in control mice (Figure S44E). These findings supported the hypothesis that STEPT treatment‐induced CD8^+^ T cells are important for the control of metastatic tumors. Based on this analysis, the STEPT system appears to trigger the activation of antitumor CD8^+^ T cells through the antigen‐presentation effect of DCs, demonstrating strong antitumor activity (Figure 6A and Figure S41F).

Studies have shown that perforin‐2 in conventional type 1 DCs (cDC1s) assists in the cross‐presentation of cell‐associated antigens into the cytoplasm [71]. Pfo probably plays the same role as perforin‐2 in immune‐antigen presentation. We observed that the pfo secreted by a small amount of EcN‐pfo, which had no obvious cell‐killing effect, was co‐located with phagosomes in BMDCs (Figure 6B). Before its insertion into the cell outer membrane of BMDCs, pfo is phagocytosed into the phagosome, together with the tumor antigen. Pfo can perforate intracellular vesicles, resulting in local rupture of the phagosomal membrane (avoiding the non‐specific hydrolysis of the antigen) and cross‐presentation of the cytoplasmic antigen, thereby accelerating the initiation of T cells [71]. This is consistent with our observation that pfo enhanced the “leakiness” of endocytic compartments in cells (Figure 6C).

Consistent with previous studies [4], DCs secreted interleukin‐12 (IL‐12) to activate T cells, and T‐cell‐derived IFN‐γ, in turn, triggered DCs to produce IL‐12 (Figure S45A). This positive feedback signal enhanced the expression and cytotoxic activity of IFN‐γ^+^ CD8^+^ T cells during treatment (Figure S45B,C; Figure 6A). The cytokines TNF‐α and IL‐2, secreted by infiltrating immune cells at the tumor site in the breast tumor‐bearing model mice, were also significantly upregulated (Figure S46A). However, the levels of inflammation‐related cytokines in serum remained stable (Figure S46B). We further evaluated the induction of systemic non‐organ‐specific antinuclear antibodies [72] under STEPT treatment. We found that G‐STEPT therapy failed to induce ANAs in either tumor model (Figure S46C), suggesting that the treatment did not induce systemic autoimmunity.

In addition, we also found that intravenous administration of programmable EcN and intratumor injection of clickable hydrogel were both necessary for successful activation of CD8^+^ T cells using the STEPT system (Figure S47 and S48). Furthermore, the antitumor ability of DBCO‐EcN‐pfo was greatly reduced in the absence of heparosan (Figure S49).

### 2.5 Immunological Memory of STEPT Treatment Provides Long‐Term Antitumor Protection

**FIGURE 7:**
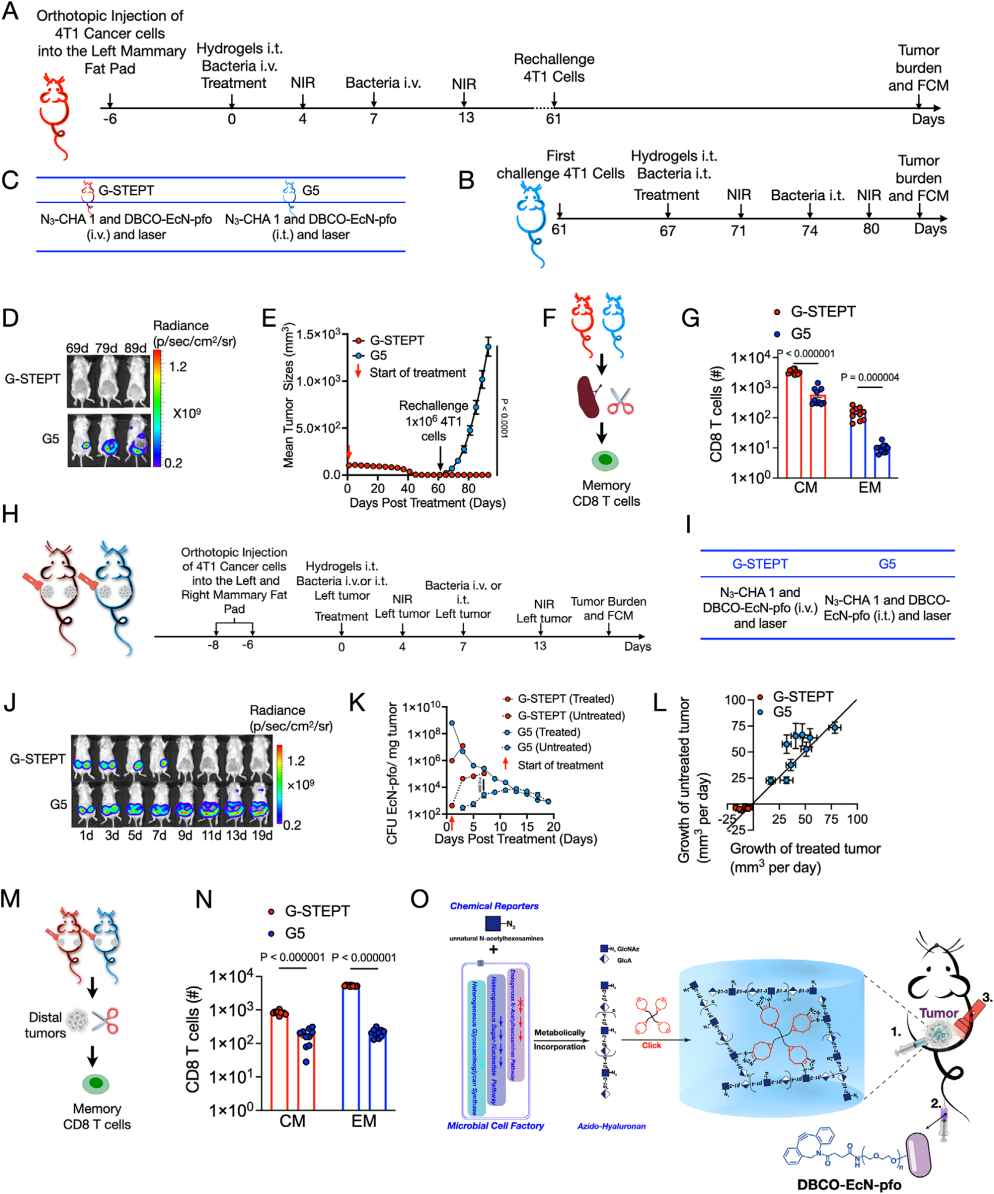
STEPT‐mediated prevention of recurrence and combat distal “cold” tumor in mice model of breast cancer. (A–G) STEPT‐mediated prevention of recurrence in a model of breast cancer. (A–C) Illustration of experimental protocols for STEPT; FCM, Flow cytometry. (B) G5 indicated the control group. (D) Representative bioluminescence images of tumor and (E) 4T1‐luc tumor growth curves of 4T1‐luc tumor‐bearing mice after the indicated treatment in (A) and (B); *n* = 10 biologically independent mice. (F) Illustration of memory CD8^+^ T cells analysis. (G) Quantitative analysis of effector memory (EM) T cells and central memory (CM) T cells in the spleen at day 93 after the indicated treatment; *n* = 10 biologically independent samples. (H–N) Usefulness of STEPT treatment to combat distal “cold” tumor models in mice. (H,I) Illustration of experimental protocols for distal cold tumor therapy in 4T1‐luc tumor‐bearing mice. (I) showed the grouping arrangement. (J) Representative bioluminescence images of 4T1‐luc tumor‐bearing mice; *n* = 10 biologically independent mice. (K) CFUs of EcN‐pfo in tumors at different time points after the indicated treatment; *n* = 10 biologically independent mice. The graphs were discontinued after day 7 because of tumor regression in the G‐STEPT group. (L) Growth rates of proximal (hot) and distal (cold) 4T1‐luc tumors after the indicated treatment. Plot of tumor growth rate (mm^3^/day) of treated compared to untreated tumors for each mouse. Dotted line indicates slope = 1, points represent means, error bars represent S.E.M.; *n* = 10 biologically independent mice. (M) Illustration of memory CD8^+^ T cells analysis by flow cytometry at day 20. (N) Quantitative analysis of the EM T cells and CM T cells in tumors at day 20 after the indicated treatment; *n* = 10 biologically independent samples. (O) Clickable hydrogel and programmable bacteria for spatiotemporally targeted therapy against solid tumors. Data in (E,G,K,L,N) expressed as the mean ± SEM. *P* values determined by unpaired two‐tailed Student's *t*‐test (G,N) or two‐way ANOVA with Bonferroni post‐hoc test (E,K).

To investigate the benefits of G‐STEPT treatment for long‐term immune memory in mice, we induced the formation of 4T1‐luc tumors (Figure 7A,B). When complete responders were re‐challenged with 4T1‐luc cells on day 61, it was found that mice treated with STEPT did not experience tumor recurrence and were resistant to re‐challenge (Figure 7A–E). In contrast, the 4T1‐luc‐tumor bearing mouse model treated with intratumor injection of DBCO‐EcN‐pfo (G5) was used as control (Figures 7B,C), which received the same batch of 4T1‐luc cells developed tumors within a week of injection (Figure 7D,E; Figure S50A). Flow cytometry analysis of spleen cells isolated on day 93 showed that the proportions of effector memory T cells (EM, CD3^+^CD8^+^CD44^+^CD62L^−^) and central memory T cells (CM, CD3^+^CD8^+^CD44^+^CD62L^+^) in the G‐STEPT treatment were higher than G5 (Figure 7F,G; Figure S50B). At the same time, the proportion of CM in the spleen was higher than that of EM. Overall, these results confirmed that STEPT treatment effectively induced immune memory, which is essential for long‐term prevention of tumor recurrence.

We also investigated whether G‐STEPT treatment could delay the growth of untreated tumors (Figure 7H,I). Our observation that STEPT treatment enhanced the functioning of tumor‐infiltrating T cells in treated tumors led us to hypothesize that lasting remission of cancer requires not only elimination of the treated tumor, but also systemic antitumor immunity to clear distant metastases (Figure 7J–L). In untreated tumors, the proportions of effector memory T cells were higher than those of central memory T cells, suggesting that central memory T cells transformed into effector memory T cells and enhanced the tumor‐based activity of effector memory CD8^+^ T cells (Figure 7M,N). These data suggested that phenotypic changes in T cells induced by STEPT treatment may explain their enhanced ability to distal tumors (Figure S50C). Based on this mechanism, the STEPT system could generate strong CD8^+^ memory T cell and prevent tumor recurrence, while inhibiting distant “cold” tumors.

In the STEPT system, the entire tumor‐fighting process involves interactions among bacteria, cancer cells, and immune cells. As shown in Figure 6A, in the initial stage of treatment, pfo molecules are released rapidly, indiscriminately killing cancer cells, and resulting in potent tumor growth inhibition together with tumor shrinkage and crusting (Figure S25A). Exposed tumor antigens were processed and presented by DCs [73] (Figures S39 and S45), triggering an adaptive tumor‐specific immune response mediated by T cells. At the same time, engineered bacteria further stimulated DCs by activating multiple innate immune system signaling pathways, including the TLRs [74], cGAS‐STING [75], and NLRs [76] (Figure S41) in this study. The antigen‐specific CD8^+^ T cells then recognized and attacked not only tumor cells in the locally treated tumor, but also distant tumor cells (Figure 7). The system produced effective vaccine‐like immune memory, which is conducive to inhibiting tumor recurrence and metastasis.

## 3 Discussion

To address the limitations of systematic delivery of bacteria and therapeutic payloads of microbial therapy, we designed a spatiotemporal targeting system for engineered *E. coli*, controlled by bio‐producing azide‐modified HA hydrogel (Figure 7O). This spatiotemporal localization strategy, called STEPT, was used to precisely adjust the interactions between bacteria and the host environment. This system features sensor and responder genetic circuits that autonomously regulate the release of payloads in TME, and was designed to improve the effectiveness and safety of cancer treatments [58]. In addition to targeting solid tumors, STEPT was also found to inhibit tumor recurrence and distant “cold” tumors. The safe and efficient preparation of azido‐HA is crucial for the spatiotemporal localization of the STEPT system. While azido‐HA can be obtained through various chemical modifications, the degree of azide substitution on HA using chemical strategies is often low, and purification can be difficult [77, 78, 79, 80]. This may result in the rejection of by‐products or unexpected physiological toxicity during clinical use. By contrast, fermentation‐derived azido‐HA can be obtained using generally‐recognized‐as‐safe microorganisms and offers the advantages of a high degree of azide substitution and abundant clickable sites.

More critically, the conjugation of DBCO‐EcN‐pfo with N_3_‐CHA 1 in situ may create a polymer spacer that prevents intercellular interactions between EcN‐pfo, cancer cells, and immune cells [44] (Figure 3E). DBCO‐EcN‐pfo uses N_3_‐CHA 1 hydrogel as a spacer barrier by click reaction with azide, which provides a therapeutic opportunity for engineered cells in tumor EcN‐pfo to not be cleared before activation of immune cells at the initial stage of the experiment, buying time for the growth and colonization of DBCO‐EcN‐pfo (Figure S24), thereby enhancing the effect of treatment. This feature indicates that the in situ bioorthogonal clicking reaction between N_3_‐CHA 1 hydrogel and DBCO‐EcN‐pfo could lead to a safer bacterial treatment strategy. Due to the complexity of tumors, we are currently exploring the conditions under which MMP can cleave Lpp‐OmpA‐pfo into pfo, which naturally exists in the tumor environment, and we will provide evidence for this in the future.

In addition, adoptive T‐cell therapy for solid tumors is limited by the apoptosis‐resistance mechanism of tumor cells and the immunosuppressive TME. The effectiveness of tumor vaccines and cell therapies is hindered by the inability of initiated T cells to penetrate tumors. Therefore, we must overcome the limitations of immunotherapy and seek new solutions for solid tumors. The STEPT system can meet these challenges. Mechanically, we found that STEPT triggered the powerful activation of DCs, leading to the induction of an initiation program to enhance endogenous T‐cells to penetrate tumors. We speculate that pfo can induce antigen diffusion from the immunosuppressive TME after destroying the physical barrier of the tumor, which may contribute to antigen spreading [16]. And the immune system can recognize and attack tumor more obviously which may play a role in resolving the heterogeneity of the tumor [81] and tumor‐induced immunosuppression [82]. In the future, we plan to further characterize the distribution of bacteria within the tumor at the single‐cell level [66]. We also aim to explore ways to solve the challenges of antigen‐loss‐mediated tumor escape and tumor antigen heterogeneity using oncolytic bacteria strategies [83, 84, 85, 86]. However, further work is also required to elucidate the mechanisms underlying the tumor and immune system changes that occur during STEPT treatment and to optimize treatment approaches. Furthermore, such optimization should aim to expand the application of STEPT as a safe, effective solid‐tumor treatment strategy.

The greatest advantage of bacterial therapy is its genetic flexibility, enabling personalized treatment and targeting of specific tumor sites [87]. Once perfected, anti‐cancer bacteria are expected to become an essential clinical tool, capable of performing functions in inhibiting and treating tumors [88, 89]. At the same time, compared with traditional immune cell anti‐cancer therapies which are very expensive, oncolytic bacteria can be produced at low cost in a simple, sustainable process. This is expected to greatly improve the accessibility of clinical cancer treatment [90]. The transition of any new treatment from laboratory bench to bedside requires a great deal of effort, and this study has demonstrated the feasibility of the STEPT strategy for improving the aggregative efficiency of engineered bacteria in tumors. In the future, we will consider using non‐natural amino acids as clickable handles and design lysogenic bacteria with passaging labels using gene‐encoding methods. In addition, we will consider the implantation of optical fiber probes for temperature measurement and micro laser probes for irradiation. Furthermore, we expect that the post‐optimized STEPT strategy will have great clinical potential for a variety of cancer indications [91].

In terms of clinical conversion, our future research direction involves the design of remote‐controlled, wearable patch light‐emitting diodes to replace NIR, which is expected to further improve clinical operability. Additionally, the selection of solid tumor types and bacterial cells to be used as treatment in this system will be broadened. For deep internal tumors in the digestive tract or other organs, endoscopic and fiber optic techniques can be utilized to precisely guide injection and irradiation with the assistance of ultrasound, computed tomography, or magnetic resonance imaging [92]. Azido‐HA hydrogels can be introduced into the postoperative tumor resection site via an indenture catheter, which is often surgically used to drain postoperative fluid.

## 4 Materials and Methods

### 4.1 Materials

Ac_4_GlcNAz (1,3,4,6‐tetra‐*O*‐acetyl‐2‐deoxy‐2‐[(2‐azidoacetyl) amino]‐β‐D‐glucopyranose, SAM209) was purchased from Jinan Samuel Pharmaceutical Co., Ltd. GlcNAc (*N*‐acetyl‐d‐glucosamine (*N*‐acetyl‐2‐amino‐2‐deoxy‐d‐glucose), B34227) was purchased from Shanghai Yuanye Bio‐Technology Co., Ltd. DBCO‐Cy5 (cyanine 5 dibenzocyclooctyne, D30F0) was purchased from Lumiprobe Ltd. Sulfo DBCO‐amine (sulfo dibenzocyclooctyne‐amine HY‐127056), chloramphenicol (HY‐B0239), kanamycin sulfate (HY‐16566A), Ampicillin (HY‐B0522), DMSO (dimethyl sulfoxide, HY‐Y0320), astragaloside IV (HY‐N0431) and D‐(+)‐xylose (HY‐N0537) were purchased from MedChemExpress LLC. DBCO‐PEG2000‐amine (PS2‐NDB‐2K) was purchased from Fansh Biosciences. Centrifugal filters (Amicon Ultra‐15 Millipore, UFC903096), acetonitrile (hypergrade for HPLC‐MS, 900667), BCN‐NHS ester ((1*R*,8*S*,9*S*)‐Bicyclo[6.1.0]non‐4‐yn‐9‐ylmethyl *N*‐succinimidyl carbonate, 744867), four‐arm PEG‐NH_2_ (JKA7011), calcein AM solution (C1359), Hoechst 33342 (bisBenzimide H 33342 trihydrochloride, B2261), anti‐HA tag‐tetramethylrhodamine (TRITC) antibody (mouse monoclonal antibody, H9037), d‐luciferin potassium salt (50227) and saporin (S9896) were purchased from Sigma Aldrich, Merck kGaA. M9 minimal salts (A507024‐0250) and 4% paraformaldehyde fix solution (E672002) were purchased from Sangon Biotech. Superfrost Plus microscope slides (12‐550‐15), chromogenic coliform agar (CM1205B), and pHrodo BioParticles (P35365) were purchased from Thermo Fisher Scientific. RPMI 1640 (Roswell Park Memorial Institute 1640, 11875093), PBS (phosphate‐buffered saline, 10010023), Dulbecco's PBS (DPBS, 14190136), penicillin–streptomycin (15070063), and FBS (inactivated fetal bovine serum, 16140089) were obtained from Gibco Life Technologies. Dulbecco's modified Eagle's medium (DMEM; 319‐005‐CL) was obtained from Wisent Bio Products. Luer lock syringes (three‐way valve injector, DP‐055) were purchased from CARENT Co., Ltd. The BE‐TRANSFLOW device and microfluidic flow control system connection kit were from BEONCHIP. Cover glass (474030‐9000‐000) was from Zeiss. Transwells (3428 and 3450) and Corning Costar ultra‐low adhesion perforated plates (CLS7007‐24EA) were from Corning. Fresh sheep red blood cell suspension (RCB001) was from Bersee. The mouse CD8^+^ T‐cell isolation kit (19853) and EasySep Mouse CD11c Positive Selection Kit II (18780) were from STEMCELL. HA tag monoclonal antibody (Alexa Fluor 488, A21287), HA tag monoclonal antibody (26183), goat anti‐mouse IgG (H+L) cross‐adsorbed secondary antibody (Pacific Blue, P31582), and lennox L agar (22700025) were from Invitrogen. The 1‐(3‐dimethylaminopropyl)‐3‐ethylcarbodiimide (EDC; 39391) and *N*‐hydroxysuccinimide (NHS; 130672) were purchased from Sigma Aldrich and Merck kGaA. Resatorvid (HY‐11109), lipopolysaccharides (LPS, HY‐D1056), hydroxychloroquine sulfate (HY‐B1370), CpG (HY‐146245C), C29 (HY‐100461), TH‐1020 (HY‐116961), H‐151 (HY‐112693), c‐di‐AMP‐disodium (HY‐12326A), NOD‐IN‐1 (HY‐100691), TriDAP (HY‐P5522), CY‐09 (HY‐103666), and QS‐21 (HY‐101092) were purchased from MedChemExpress. Heat‐killed *L. monocytogenes* (HKLM) and flagellin from *S. typhimurium* (FLA‐ST) were purchased from InvivoGen.

### 4.2 Instrumentation

NanoDrop One/One^C^ Microvolume UV–vis Spectrophotometer (701‐058112) was obtained from Thermo Scientific. Agilent Zorbax C18 5‐µm HPLC column (870100‐914), Agilent Purification HPLC AP‐SMLADI10‐MSI, and UHPLC‐Q Exactive Orbitrap mass spectrometer were obtained from Agilent. HPLC‐MS analyses were performed on a liquid chromatograph in conjunction with an ESI‐Q‐TOF quadrupole time‐of‐flight tandem high‐resolution mass spectrometer (Maxis II, Bruker) and a Luna analytical hydrophilic interaction chromatography (HILIC) column (200 Å, 150 × 2.0 mm, 3 µm; 00F‐4449‐B0, Phenomenex). Fluorescent images of animals were obtained using the in vivo imaging system (IVIS) Spectrum (IVIS Kinetic, PerkinElmer). We also used a ZE5 Cell Analyzer (Bio‐Rad), an automatic colony counter (Czone 8 colony counter, Czone), an ultra‐high resolution multi‐mode ultrasonic PA imaging system for animals (Vevo LAZR, VisualSonics), and a TissueFAXS SPECTRA imaging system (TissueGnostics). The temperature probe is from Neoptix (T1‐02‐B05); 808‐nm NIR laser (MDL‐III‐808nm‐2.5W‐BK10063) is from Changchun New Industries Optoelectronics Technology Co. Ltd.

### 4.3 Bacterial Strain Construction

We chose the HA synthase from *Streptococcus zooepidemicus* (szHasA) to replace pmHAS as the glycan polymerase in recombinant cells. Unlike the cytosolic pmHAS, type I HASs from *Streptococcus* contain a single catalytic domain and secrete the nascent HA polymers through a transmembrane channel formed by their membrane‐embedded segment. We hypothesized that disrupting de novo UDP‐GlcNAc biosynthesis in *B. subtilis* would force the cells to survive on GlcNAc (or GlcNAz) via the artificial nucleotide sugar salvage pathway, facilitating the incorporation of GlcNAz into HA polymers. In wild‐type bacteria, the five‐step de novo UDP‐GlcNAc biosynthetic pathway is responsible for converting fructose to UDP‐GlcNAc. Glutamino‐6‐phosphofructose aminotransferase (GlmS) is a highly conserved enzyme in this pathway and is essential for the viability of *B. subtilis* under normal growth conditions. However, if extracellular GlcNAc is provided as a supplement, *B. subtilis glmS*Δ strains can be rescued via the functional *nagP*‐*ypqE*‐*yyzE* pathway. Next, because GlcNAz is tolerated by biosynthesis machinery consisting of N‐acetylhexosamine 1‐kinase (NahK) from *Bifidobacterium longum* and UDP‐N‐acetylhexosamine pyrophosphorylase from *Homo sapiens* (AGX1), we introduced these two exogenous enzymes into *B. subtilis* (*glmS*Δ‐168) [50]. *NahK* and *AGX1* were placed in the *B. subtilis* 168 genomes under the control of the constituent Pveg promoter. The sz*HasA* gene from *S. zooepidemicus* was placed in vector pHT43 under the control of the inducible PxylA promoter. The final *B. subtilis* strain (genotype *GlmS*Δ‐*NahK*‐*AGX1‐szHasA*‐168) was named 168SSHA.

The plasmids involved in the construction of recombinant *B. subtilis* and EcN strains are listed in Table S1. pUC57‐*neo* was digested with *Bam*HI to obtain a neomycin‐resistance gene between homologous flanking fragments of the gene *glmS*. After recovery and purification, the fragment was transferred into *B. subtilis* 168 to achieve a genomic knockout of *glmS*, generating strain *BS*168S (*glmS*△‐168). pUC57‐Pveg‐*AGX1*‐Pveg‐*NahK* was cut with *Acc*65I and *Sma*I to obtain homologous arm fragments of the neomycin‐resistance gene with the kanamycin‐resistance gene. The Pveg‐*AGX1*‐Pveg‐*NahK* gene fragment with homologous flanking sequences of the neomycin‐resistance gene was integrated into the genome of *BS*168S (*glmS*△‐168) via homologous recombination, and positive transformants were obtained as strain *BS*168SS (*glmS*Δ‐*NahK*‐*AGX1*‐168).

The expression frame of *szHasA* was amplified by polymerase chain reaction (PCR) of pUC57‐PxylA‐*szHasA* with 5′‐*Acc*65I and 3′‐*Sma*I restriction sites using primers *szHasA*_F/*szHasA*_R. The PCR product was then linked to pHT43 to produce pHT43‐PxylA‐*szHasA*.

Gene insertions were confirmed using sequencing primers. To verify the homologous recombinatorial knockout of *glmS*, primers glmS_F (*B. subtilis*)/glmS_R (*B. subtilis*) and neo_F/neo_R were used. To verify neomycin‐resistance gene knockout by homologous recombination, primers NahK_2F/NahK_2R and tuaD_F/tuaD_R were used. For pHT43‐PxylA‐*HasA*, primers *szHasA*_F/ *szHasA*_R were used. Receptive *BS*168SS (*glmS*Δ‐*NahK*‐*AGX1‐*168) was electrotransformed with pHT43‐PxylA‐*HasA* to generate *BS*168SSHA (*glmS*Δ‐*NahK*‐*AGX1*‐*szHasA‐*168).

Lpp‐BRP‐*phoA* and Axe‐Txe expression frames, pCadC‐Lpp‐ompA‐*pfo*, Lpp‐ompA‐*pfo*‐HA tag, and *mCherry* were cloned into the pBV220 vector (Genscript) individually or simultaneously. The expression frame of Lpp‐BRP‐*phoA* was amplified by polymerase chain reaction (PCR) using primers phoA F/phoA R. The expression frame of Axe‐Txe was amplified by polymerase chain reaction (PCR) using primers axetxe F/axetxe R. The expression frame of PCadC‐Lpp‐ompA‐*pfo* was amplified by polymerase chain reaction (PCR) using primers CadC F/pfo R. The expression frame of PL‐PR‐mCherry was amplified by polymerase chain reaction (PCR) using primers pLpR‐mCherry F/pLpR‐ mCherry R. The expression frame of PCadC‐mCherry was amplified by polymerase chain reaction (PCR) using primers CadC F/CadC‐mCherry R. The PCR product was then linked to pBV220 to produce pBV220‐BRP‐*phoA*‐pCadC‐Lpp‐ompA‐S_MMP_‐*pfo*‐Axe‐Txe. The luxCDABE expression frame amplified by polymerase chain reaction (PCR) using primers luxCDABE F/luxCDABE R was cloned into the pGEN vector (Genscript). The HA tag is sequence YPYDVPDYA added to the C‐terminal of pfo. Primer sequences are listed in Table S2. Additional genes and all PCR primers were synthesized using integrated DNA techniques. Plasmids were constructed using Gibson assembly or standard restriction digestion and ligation cloning, and transformed into DH5α competent cells (Invitrogen). Gene knockout in EcN uses CRISPR Cas9 to obtain EcN∆*KfiC* [36]. All constructed plasmids were electrotransformed into EcN or EcN∆*KfiC* and grown in Luria‐Bertani broth at 37°C with shaking at 180 rpm, with or without appropriate antibiotics.

### 4.4 Purification of Azido‐HA and HPLC‐MS Disaccharide Analysis

*BS*168SSHA was activated overnight in LB broth containing GlcNAc (100 µg mL^−1^), chloramphenicol (5 µg mL^−1^), and kanamycin (50 µg mL^−1^) at 37°C with 225 rpm shaking. On the second day, the bacteria were collected by centrifugation (4427 ×*g*, 5 min), washed three times with PBS, and transferred to M9 medium. After allowing the cells to consume the original GlcNAc for 1 h, GlcNAz (final concentration, 500 µg mL^−1^) was added, and the culture was continued at 37°C with 225 rpm shaking until the optical density at 600 nm (OD_600_) reached 0.45. Xylose (20 mg mL^−1^) was then added, and the culture was continued for a further 6 h at 37°C with 225 rpm shaking. In the control group, the conditions were the same except that GlcNAc was added without azide monosaccharide.

Azido‐HA was purified from the fermentation broth of recombinant strains as follows. Fermentation cultures were centrifuged (5000 ×*g*, 20 min, 4°C) to collect the cells. Protein impurities were removed from the culture supernatants by the addition of trichloroacetic acid (10% w/v), incubation on ice for 2 h, and centrifugation (5000 ×*g*, 20 min, 4°C). The supernatants were neutralized using 0.5 M NaOH, followed by the addition of 0.5 M NaCl and pH adjustment to 7.0, then incubated for 1 h to remove impurities. The azido‐HA was precipitated by adding two volumes of ethanol, followed by incubation at 4°C for 18 h. The HA salt was collected by centrifugation (5000 ×*g*, 20 min, 4°C) and then resuspended in triple‐steamed water, to which one volume of 0.1 M acetate buffer and acetone was added and incubated at −20°C for 2 h to remove nucleic acid impurities. The HA salt was re‐harvested by centrifugation (5000 ×*g*, 20 min, 4°C), and the pellet was washed with two volumes of absolute ethanol and dissolved in triple‐steamed water. A vacuum freeze dryer was used to obtain solid azido‐HA, which was stored at −20°C. All procedures are performed under sterile conditions. For HPLC‐MS disaccharide analysis, solid azido‐HA was suspended in 200 µL triple‐steamed water to a concentration of 0.1 mg mL^−1^ containing 50 mM Tris‐HCl (pH 7.0) and 0.2 mg mL^−1^ AsChnAC, an endo‐eliminase [54]. Protein was removed by ultrafiltration (30‐kDa cutoff), and the disaccharides were analyzed by HPLC and high‐resolution MS under the following liquid chromatography conditions: mobile phase A, 5 mmol/L ammonium acetate aqueous solution; mobile phase B, 5 mmol/L ammonium acetate, 98% acetonitrile solution. The liquid phase gradient was 0–5 min, 95% B; 5–15 min, 95%–77% B; 15–20 min, 77%–40% B; 20–24 min, 40% B; 24–26 min, 40%–95% B; 26–30 min, 95% B. The flow‐rate was 0.15 mL/min. The absorbance of the elution peak was measured at 232 nm. MS parameters were as follows: spray voltage, 4.5 kV; spray gas flow rate, 20 arb; capillary transfer tube temperature, 200°C; scanning range, 50–800 *m*/*z*. All data were processed and analyzed on Bruker Data Analysis software. The quality errors were all < 20 ppm.

### 4.5 Cell Culture

WEHI164 cells, 4T1‐luc cells, B16‐OVA cells, MCF7 cells, and MCF7‐GFP cells are gifts from Professor Fengshan Wang. Human umbilical vein endothelial cells (HUVECs, PCS‐100‐013) were purchased from American Type Culture Collection (ATCC) and propagated in RPMI 1640 medium containing 10% FBS and supplemented with 100 U/mL penicillin and 100 µg mL^−1^ streptomycin at 37°C in a 5% CO_2_ incubator. All cell lines tested negative for mycoplasma in a DNA‐based PCR test (Beyotime). All cells were obtained with written informed consent and collected using a standard protocol approved under the Review Board Protocol of Shandong University.

### 4.6 Animals

All animal experiments were conducted in accordance with guidelines approved by the Animal Care and Use Committee of Shandong University Cheeloo College of Medicine, and experiments were approved by the Animal Ethics Committee of Shandong University (Approval number 230085). BALB/c and C57BL/6 mice were purchased from Beijing Vital River Laboratory Animal Technology Co., Ltd (Beijing, China) and housed by the Shandong University Medical School Animal Care Facility under pathogen‐free conditions. All mice used for animal experiments were 6–8 weeks of age, and both sexes were included in the study, except that only female BALB/c mice were used for the engraftment of 4T1 breast cancer cells. Mice were maintained in specific‐pathogen free conditions, and each group was kept in a separate cage and allowed to eat and drink freely. Artificial light was provided in a 12 h/12 h cycle. For all of the animal experiments, mice were randomly allocated to groups. The investigator was aware of these group allocations, as demanded by the experimental design.

### 4.7 Experimental Animal Models

For animals carrying tumors, our university guidelines stipulate that euthanasia is required when the tumor burden reaches 2 cm in diameter or is recommended by a veterinarian. BALB/c mice were blindly, randomly divided into groups. A tumor model was established by S.C. injection of mouse fibrosarcoma WEHI164 cells or by orthotopic injection of 4T1 luciferase breast cancer cells into a mammary fat pad. C57BL/6 mice were blindly, randomly divided into groups. A tumor model was established by S.C. injection of mouse melanoma B16‐OVA cells. All cells were cultured in RPMI‐1640 medium supplemented with 10% FBS and 1% penicillin‐streptomycin, and maintained in a tissue culture incubator at 37°C and 5% CO_2_. The tumor cells were implanted at a concentration of 5 × 10^7^ cells/mL in RPMI 1640 (without phenol red) at a volume of 100 µL (5 × 10^6^ cells/implant). Before the experiment, the tumors grew to approximately 100 mm^3^ on average. The total tumor volume was quantified using caliper measurements of tumor length and width (length × width^2^ × 0.5). For experiments that required larger tumors to grow, we waited until the tumor had grown to approximately 270 mm^3^ on average before starting the experiment. Mice were euthanized by CO_2_ inhalation. The maximum tumor diameter of 20 mm permitted by the ethics committee was not exceeded.

### 4.8 Synthesis of the Crosslinking Agent PEG‐tBCN

BCN‐NHS ester was dissolved in 800 µL of a 50:50 mixture of DMSO and methanol (25.5 mM), to which 14.2 µL triethylamine and 52.8 µL four‐arm PEG amine were added, mixed by vortexing (5 min), and then subjected to analysis on an HPLC analytical system to confirm the reaction. The PEG‐tBCN was directly purified by preparative HPLC. The collected material was evaporated on a rotary evaporator to remove methanol, then frozen and lyophilized to remove water. The compound was confirmed by NMR analysis and FT‐NIR. The collected PEG‐tBCN was resuspended in DMSO at a 10 mM concentration, from which 50–200 µL aliquots were prepared to prevent excessive freeze–thaw cycles of the stock.

### 4.9 Quantification of Azides in Azido‐HA Hydrogels

Quantification of azide groups on HA was performed by measuring the decrease in DBCO absorbance upon incubation of azido‐HA with an excess amount of DBCO. Azido‐HA samples were dissolved in PBS at 1% w/v (1 mg in 1 mL). Sulfo DBCO‐amine (700 nmol) was dissolved in 500 µL PBS. Azido‐HA solution (500 µL) was added to DBCO‐amine solution (500 µL), mixed, and incubated overnight. Samples were analyzed on a NanoDrop One/One^C^ Microvolume UV–vis Spectrophotometer at a wavelength of 308 nm. Azide concentrations were determined from the decrease in absorbance at 308 nm of DBCO‐amine with azido‐HA sample compared with that of the DBCO‐amine (extinction coefficient at 308 nm = 10,600 M^−1^cm^−1^). The degree of substitution was calculated using the mass and MW of azido‐HA, taking into account the change in MW due to azide modification.

$$\mathrm{DS}\;=\frac{\mathrm{mol}\;\mathrm{azide}\times \mathrm{MW}\;\mathrm{azido}\;\mathrm{HA}}{\mathrm{mass}\;\mathrm{azido}\;\mathrm{HA}-\mathrm{mol}\;\mathrm{azide}\times \mathrm{MWazide}}$$

In this equation, mol azide = moles of azide in solution calculated from NanoDrop, MW azido‐HA = MW of azido‐HA; mass azido HA = mass of azido‐HA in solution; MW azide = MW of the azide contribution to the azide‐HA.

### 4.10 Crosslinked Formulation and Rheology Testing of Hydrogels

To prepare N_3_‐CHA 1 hydrogel for in vivo experiments, 800 µL azido‐HA (2 mmol/L) was dissolved in PBS and pulled into a 1‐ml Luer lock syringe. 80 µL PEG‐tBCN (2 mmol/L) crosslinker solution was added to a separate 1‐ml Luer lock syringe and the two syringes were joined, being careful to avoid introducing air into the hydrogel. Hydrogels were formed by mixing (pushing and pulling the syringes back and forth 20 times). For the preparation of three other hydrogel ratios, namely N_3_‐CHA 2 (1:15), N_3_‐CHA 3 (1:12), and N_3_‐CHA 4 (1:5), the 2 mM PEG‐tBCN volumes were 53, 67, and 160 µL, respectively. Rheological analysis was performed on a rheometer (MCR302, Anton Paar). Coated in mineral oil to prevent drying out, the hydrogel samples exhibited a 1% shear strain (oscillation) at an angular frequency of 10 rad/s. Hydrogels are formed when the storage modulus exceeds the loss modulus. A linear shear rate sweep from 0.01 to 100 s^−1^ was used to characterize the shear‐thinning kinetics of the hydrogels. Next, injectability was tested by using a syringe with a 25‐gauge needle. The morphology of the hydrogels was examined via field‐emission scanning electron microscopy imaging. Digital images were taken with an iPhone 7 Plus.

### 4.11 Repeat Replenishment of the Azido‐HA Hydrogel Reservoir by DBCO‐Cy5 Before and After Crosslinking

Because the purpose of azido‐HA hydrogel was to replenish drug repositories, DBCO‐Cy5 molecules were used to visualize retention and filling after replenishment in vivo. Female BALB/c mice were treated with a 100 µL S.C. injection of azido‐HA or N_3_‐CHA1 hydrogel (1 mg mL^−1^ in PBS) in one shoulder and a 100 µL S.C. injection of HA (1 mg mL^−1^ in PBS) in the other shoulder. Each mouse was also given DBCO‐Cy5 (10 µL, 5 mM in DMSO) intravenously every 2 days. Imaging was performed 24 h after each injection. Azido‐HA was injected into DBCO four times before crosslinking and 80 times after crosslinking. Fluorescence imaging was conducted using 646 nm excitation and 662 nm emission filters to visualize Cy5, and the signal was quantified as the total radiant efficiency of the biopolymer injection site. Briefly, mice received subcutaneous injection with azido‐HA and intravenous injection with Cyanine 5 DBCO (DBCO‐Cy5), a clickable fluorescent molecule, on the same day. Thereafter, these circulating molecular simulates were injected every 2 days. As expected, fluorescence appeared around the azido‐HA injection site, suggesting that azide groups on HA captured DBCO‐Cy5 molecules. Additionally, the fluorescence intensity gradually increased with the increasing number of DBCO‐Cy5 refills, exhibiting a roughly linear increase in fluorescence at 24 h after each administration of DBCO‐Cy5.

### 4.12 Assessment of In Vivo Stability and Histology of Azido‐HA Hydrogel Before and After Crosslinking

BALB/c mice were treated with a 100‐µL shoulder S.C. injection of azido‐HA (1 mg mL^−1^ in PBS) or N_3_‐CHA1 (1 mg mL^−1^ in PBS) hydrogel, which was modified with DBCO‐Cy5 fluorophore to allow for in vivo detection. Starting 48 h post injection, in vivo hydrogel residency was assessed over 100 days using the IVIS imaging system on days 1, 2, 3, 4, 7, 10, and 14, then twice or thrice weekly until the end of the experiment. Fluorescence imaging was conducted using 646‐nm excitation and 662‐nm emission filters to visualize DBCO‐Cy5. The percentage of the biopolymer remaining was determined by fluorescence radiant efficiency at the injection site. A fluorescent signal remained evident at the biopolymer injection site for approximately 4 months and was quantified as total radiant efficiency in vivo.

Mice received a 100‐µL shoulder S.C. injection of azido‐HA (1 mg mL^−1^ in PBS) or N_3_‐CHA1 hydrogel (1 mg mL^−1^ in PBS). Mice were sacrificed after day 140, major organs (heart, liver, kidneys, lung, and spleen) were removed, embedded in paraffin, sliced, and stained with hematoxylin and eosin (H&E). Histological specialists assessed the inflammatory response to the hydrogels.

### 4.13 DBCO Labeling of EcN

EcN strains were cultured in LB broth and grown overnight (12 h). A 1:100 dilution of bacteria in the same medium was grown to OD_600_ = 0.1–0.4, then collected by centrifugation (2000 ×*g*, 10 min, room temperature). The bacteria were washed three times with DPBS and then resuspended in 1 mL DPBS containing 100 µM DBCO‐PEG2000‐amine to a concentration of 2.5 × 10^8^ colony‐forming units (CFU)/mL. We verified the accuracy of the bacterial counts through repeated dilution and high‐throughput methods, EDC (0.55 mg) and NHS (0.65 mg) were added to the bacterial suspension (pH = 7.2–7.4). In amide condensation, EDC can activate carboxylic groups to form unstable *O*‐acyl groups. The addition of NHS to the reaction mixture stabilizes the active carboxyl group and subsequently improves the conversion of amide condensation. After stirring at 37°C for 2 h, the bacteria were collected by centrifugation at 2000 ×g for 3 min at room temperature, washed with cold sterile DPBS three times, and normalized to the required number of CFU. The labeled EcN cells were then used for treatment, flow cytometry analysis, and fluorescence microscopy. Because the forward scattering (FSC) and side scattering (SSC) values of bacterial cells are relatively small, the FSC and SSC thresholds were set to the minimum values available in flow cytometry (200 for both FSC and SSC in ZEN) and the FSC and SSC voltages were adjusted to capture the bacterial cell population.

### 4.14 Sodium Dodecyl Sulfate (SDS)‐Polyacrylamide Gel Electrophoresis (PAGE) Analysis of Protein

EcN strains were grown in LB broth overnight and normalized by OD_600_. To perform SDS‐PAGE of secreted proteins, 1 mL supernatant from an overnight culture (OD_600_ = 10) was incubated on ice with 250 µL 50% trichloroacetic acid (w/v) for 20 min. The precipitated protein was formed into pellets by centrifugation (18,000 ×g, 10 min, 4°C), washed twice with 250 µL cold water and acetone, dried at 95°C for 2–5 min, dissolved in 100 µL protein sample buffer (B7703, New England Biolabs), and boiled for 10 min at 95°C before loading onto an SDS‐PAGE protein gel (Bio‐Rad).

### 4.15 Hemolysis Test

The hemolytic activity of EcN was measured by incubating culture supernatant samples with 7 × 10^7^ sheep red blood cells (RBCs) for 45 min at 37°C. After centrifugation (200 ×*g*, 5 min, 4°C), the levels of hemoglobin released by the RBCs were estimated from absorption measurements at 406 nm using NanoDrop One/One^C^ Microvolume UV–vis spectrophotometer and expressed as a percentage of hemolytic activity. Maximum activity (positive control) was obtained by osmotic dissolution of RBCs with water and was considered to represent 100% hemolysis. For the negative control, RBCs were re‐suspended in saline in the absence of a protein sample.

### 4.16 Structured Illumination Microscopy (SIM) Imaging of EcN and Mammalian Cells

Transwell analysis was used to analyze the tumor‐killing ability and NIR‐induced pfo activity of DBCO‐labeled EcN‐pfo‐HA. HUVECs (grown in RPMI‐1640 medium containing 10% FBS) were deposited on the bottom of the apex chamber of the transwell unit (3428). A polylysine‐treated coverslip was placed in the lower chamber, which was cultured with MCF7 cells (grown in RPMI‐1640 medium containing 10% FBS) and N_3_‐CHA 1 mixture. DBCO‐EcN‐pfo‐HA (200‐µL samples of logarithmic growth cultures) dispersed in DPBS at a density of 1 × 10^8^ colony‐forming units (CFU) mL^−1^ were added to the top chamber, and the assembled device was incubated at 37°C. Three rounds of periodic NIR were applied under the following conditions: 0.308 W/cm^2^ and 5 cm for 5 min ON; and 5 min OFF. SIM imaging of tumor‐killing ability at 0.5, 1, 3, and 5 h was performed after staining with 2 µg Hoechst 33342, 1 µM calcein AM, and 1 µM anti‐HA tag‐tetramethylrhodamine (TRITC) monoclonal antibody. For analysis of NIR‐induced pfo activity, SIM imaging was performed following staining with 2 µg Hoechst 33342, 1 µM calcein AM and 1 µM anti‐HA tag‐tetramethylrhodamine (TRITC) monoclonal antibody staining at 5 h. The control groups were treated the same as the experimental groups, except that they did not receive NIR treatment.

The ability of DBCO‐EcN‐pfo‐HA to combine with N_3_‐CHA 1 was also analyzed using a transwell system. HUVECs (grown in RPMI‐1640 medium containing 10% FBS) were deposited on the bottom of the apex chamber of the transwell unit (3428). A polylysine‐treated coverslip was placed in the lower chamber, which was coated with N_3_‐CHA 1 labeled with DBCO‐Cy5. 200‐µL samples of logarithmic growth cultures of DBCO‐EcN‐pfo‐HA dispersed in DPBS at a density of 1 × 10^8^ CFUs mL^−1^ were each added to the top chamber separately, and the assembled device was incubated at 37°C. Three rounds of periodic NIR were applied under the following conditions: 0.308 W/cm^2^ and 5 cm for 5 min ON; 5 min OFF. Then, EcN‐pfo‐HA labeled with 1 µM HA Tag Monoclonal Antibody (Alexa Fluor 488). SIM imaging was performed 1 h later.

### 4.17 Microfluidic Chip System

Microfluidic chip devices bypass the cardiopulmonary system to simulate the vascular environment at tumor sites. We used a BE‐TRANSFLOW microfluidic device to simulate EcN penetration of blood vessels. HUVECs were pre‐cultured in a culture tank to simulate vascular epithelium. N_3_‐CHA 1 was added to the culture tank, and DBCO‐modified EcN‐luxCDABE and unlabeled EcN‐luxCDABE (10^8^ CFUs/mL in DPBS) were simultaneously circulated via pumping at a rate of 0.25 µL/min. The distribution and CFUs of EcN‐luxCDABE at each time point were measured using an IVIS.

### 4.18 3D Tumor Spheroid Experiment

Tumor spheroids are produced by seeding cells in round‐bottom, ultra‐low attachment 96‐well plates (Corning). Each well contained 2500 MCF7‐GFP (green fluorescent protein) cells in 100 µL RPMI‐1640 containing 10% FBS, without antibiotics, to which N_3_‐CHA 1 (or HA for the control group) was added. The plates were spun at 300 ×*g* for 5 min to aggregate the cells at the bottom of the plates, then placed in a tissue culture incubator for 4 days. DBCO‐labeled EcN‐pCadc‐mCherry‐HA (≈10^6^ CFUs/well), which had been cultured overnight in a vibrator at 37°C to induce a quiescent state, was inoculated into wells containing 4‐day mature tumor spheroids and returned to a tissue culture incubator. After 2 h of co‐culture, the medium was removed. Images of spheroids were obtained using the EVOS FL Auto 2 cell imaging system (Invitrogen). The range and accessories were programmed using Celleste imaging analysis software (Thermo Fisher Scientific).

Bacterial colonization of the tumor spheroids was quantified using colony counting. Briefly, spheres containing bacteria on the bottom of the wells were repeatedly washed with DPBS (200 µL). After washing, the spheroids were re‐suspended in DPBS (100 µL) and homogenized using a sterile tip mechanical dissociation and repeated pipetting. Microscopy confirmed the destruction of the spheroids, and dilutions were used to inoculate appropriate agar plates.

### 4.19 In Vivo Administration of STEPT

When the average tumor growth reached approximately over 100 mm^3^, intratumor injection of N_3_‐CHA 1 (40 µL) was initiated. One hour later, DBCO‐EcN‐pfo was administered intravenously. EcN‐pfo cultures were grown overnight in LB broth, then diluted 1:100 in LB broth and grown in a vibrator at 37°C to OD_600_ = 0.1–0.4 (mid‐logarithmic stage). After labeling EcN with DBCO as described above, the cultures were centrifuged (2000 ×*g*, 3 min, room temperature), washed three times with cold sterile DPBS, and then normalized to the desired CFU. Unless otherwise indicated, all were administered intravenously at a dose of 5 × 10^8^ cells/mL in DPBS and a total volume of 100 µL per mouse. On day 4, the mouse tumors were irradiated with three rounds of periodic NIR treatment (808 nm, 0.308 W/cm^2^ at 5 cm for 5 min ON; 5 min OFF). On day 7, the same dose of DBCO‐EcN‐pfo was again administered intravenously, and on day 13, the mouse tumors were irradiated with NIR again.

### 4.20 Photothermal Therapy (PTT)

For mice with tumors, 4 days after the first or 6 days after the second intravenous injection of bacteria, an 808‐nm NIR laser (MDL‐III‐808nm‐2.5W‐BK10063, Changchun New Industries Optoelectronics Technology Co. Ltd.) was used to periodically irradiate the tumors for three rounds at a power density of 0.308 W/cm^2^ at 5 cm for 5 min ON; 5 min OFF. The tumor temperature was recorded by an infrared camera (FLIR E50).

A temperature conduction device (Figure S17) was used to adjust the power density of NIR for the exploration of the temperature changes of the tumor. The NIR probe was placed 5 cm away from the tumor edge in the mice. The NIR probe was directed to the intra‐injection site of the azido‐HA, and a thin fiber optic temperature probe (Neoptix, T1‐02‐B05) was temporarily implanted into the tumor to measure the internal tumor temperature during heating. The custom probe had a sensing tip with a diameter of 400 µm and a length of < 2 mm. We shaved the hair from the tumor site in the treatment group, inserted a 25‐gauge needle into the tumor to guide the temperature sensing probe, inserted a fiber optic probe into the path created by the needle, and secured the probe with duct tape, running a closed‐loop thermal measurement script on the computer to measure the temperature signal (Qualitrol KIT‐057 Portable Optical Fiber Thermometer). To precisely increase the tumor temperature, the following NIR conditions were fixed: wavelength, 808 nm; irradiation distance, 5 cm; irradiation time, 5 min ON and 5 min OFF for each cycle, for three cycles lasting a total of 30 min. Effective thermal control was achieved by modifying the power density of the NIR to achieve the desired temperature in the target tissue.

### 4.21 Mouse Imaging

Mice were treated with isoflurane air anesthesia for 10 min. For bioluminescence or fluorescence imaging, the following parameters were used: exposure time, 1 s; binning, medium; f‐stop, 2; electron multiplying gain, off. For fluorescence imaging, the excitation and emission filters were based on the Ex and Em wavelengths of different fluoresceins (DBCO‐Cy5: Ex, 646 nm; Em, 662 nm). Photography parameters included the following: binning, medium; f‐stop, 16; overlay. Bioluminescence or fluorescence intensity in selected regions of interest (ROIs) was quantified using the IVIS Spectrum imaging software.

### 4.22 Biological Distribution and Imaging In Vivo

The DBCO‐EcN‐luxCDABE strain used in this study incorporates a luxCDABE box that can be visualized using the IVIS Spectral Imaging System and quantified using the Living Image software (version 4.3.1.16427). In a mouse model of breast cancer, tumor growth was monitored by bioluminescent imaging of fluorescein in 4T1‐luc cells. Bioluminescent imaging was initiated 15 min before the injection of 150 mg/kg D‐luciferin potassium salt (50227, Sigma Aldrich). Living Image software was used to quantify the luminous intensity of the ROI of each mouse. For fibrosarcoma and breast cancer model mice, images, body temperature, and body weight of the mice were obtained daily from the day of administration until the end of the study. At the study endpoint, the mice were euthanized with carbon dioxide, and the tumors and organs (spleen, liver, kidneys, heart, and lung) were extracted, weighed, and imaged. During tumor dissection, the areas away from blood vessels were necrotic, and the areas near blood vessels were marginal, and CFUs were counted separately under sterile conditions. Later, they were homogenized using a mild MACS tissue separator (C Tubes, Miltenyi Biotec). Tissue homogenates were serially diluted with sterile PBS, plated on LB agar containing ampicillin, and incubated overnight at 37°C. CFUs were counted the next day. Calculated CFUs/mg of tissue were then used to extrapolate the total tissue burden using total tissue weights.

### 4.23 PA Imaging

At different time points (2, 6, 12, and 24 h) post intravenous injection of bacteria, mouse tumors were imaged by a PA imaging system. The central region of the tumor was selected as the ROI with which to calculate the average tumor PA signal.

### 4.24 Histological Analysis

To determine the proportion of loose tumor tissue, 4T1‐luc breast cancer mice were euthanized on day 2 after STEPT treatment, the tumors were removed, harvested, fixed in 4% paraformaldehyde, embedded in paraffin, sectioned, and stained with Haematoxylin and eosin staining (H&E) using routine methods, in accordance with the manufacturer's protocol. Tissue segmentations were observed using a TissueFAXS SPECTRA imaging system (TissueGnostics). Normal/loose tissue segmentation analysis was performed by inform software (Akoya Biosciences).

### 4.25 Flow Cytometry Analysis

The tumor, spleen, and the ipsilateral inguinal lymph nodes near the tumor of tumor‐bearing mice were collected under sterile conditions using an autoclaved tool, and the weight was recorded. The spleen was crushed and lysed using a mouse erythrocyte lysis kit (WL2000; R&D Systems), and the remaining spleen cells were analyzed by flow cytometry. The ipsilateral inguinal lymph nodes nearby those tumors were homogenized into single‐cell suspensions for flow cytometry assay. For flow cytometry analysis of immune cells within the tumor, the tumor was collected and cut into small pieces within a specified time after treatment, then digested in Hank's balanced salt solution (60147ES76, Yeasen) containing 2 mg mL^−1^ collagenase V (40511ES60, Yeasen) and 0.05 mg mL^−1^ DNAse I (10104159001, Sigma‐Aldrich) for 30 min at 37°C followed by a 40% Percoll (17089101, Cytiva) purification step, subsequent erythrocyte lysis, and an enrichment of CD45^+^ cells as described: The cells were then passed through a 70‐µm cell filter to obtain a single‐cell suspension and cleaned with FACS buffer (DPBS, 2% FBS). The single‐cell suspension was pre‐blocked at 4°C for 30 min with CD16/32 antibody (101319, Biolegend). Then followed by a 15 min incubation on ice with biotinylated anti‐CD45 (103104, Biolegend). Cells were washed and incubated with streptavidin beads (557812, BD Biosciences) for 20 min, followed by a 5 min incubation in an EasySep magnet (18000, STEMCELL). Cells poured out from the magnet were discarded, and remaining cells were used for flow cytometry analysis. The following Biolegend antibodies were incubated at the indicated dilution ratios: CD45 (147707, 1:100), CD3 (100235 and 100203, 1:100), CD4 (100421, 1:100), CD8 (100728, 100803, and 100721, 1:100), Tim‐3 (134019, 1:50), CD11b (101205 and 101215, 1:100), F4/80 (123113, 1:100), CD11c (17305, 1:100), MHC II (750281, 1:100), CD80 (104723, 1:100), CD86 (159203, 1:100), PD‐1 (135213, 1:100), Gr‐1 (108405, 1:100), NK1.1 (156513, 1:100), CD44 (156005, 1:100), CD62L (104437, 1:50). Cells were stained at 4°C for 10 min with Zombie Yellow Fixable Viability Kit (423104, 1:100) and washed in FACS buffer to eliminate dead cells.

For intracellular and intranuclear staining after surface staining, the cell suspension was fixed and permeated with a True‐Nuclear Transcription Factor Buffer Set (424401; Biolegend) in accordance with the manufacturer's protocol. Cells were stained with the following intracellular or intranuclear antibodies: IFN‐γ (505817, 505805, and 505826, 1:50), TNF‐α (506323, 1:50), and Ki67 (652421, 1:100) from Biolegend, c‐jun from Invitrogen (MA5‐15881, 1:50), and Rabbit anti‐Mouse IgG (H+L) Cross‐Adsorbed Secondary Antibody, Alexa Fluor 488 (A‐11059, Invitrogen) were analyzed by flow cytometry. FlowJo (version 10.0.0.0) was used to analyze the data.

### 4.26 DC Maturity Assay In Vitro

Cells with a maturity level of about 20% CD86^+^ CD80^+^ cells among CD11c^+^ cells were collected at 5–7 days and used for the study. BMDCs (5 × 10^5^ cells/mL) and different inhibitors were added to the lower transwell chamber, and 4T1 cells (1 × 10^5^ cells/mL) and bacteria (5 × 10^7^ CFUs) were added to the upper chamber. The upper chamber was irradiated by NIR under STEPT conditions, and the cells were cultured for 12 h before collecting for flow cytometry analysis. For flow cytometry analysis, BMDCs were first incubated with anti‐CD16/32, then stained with fluorescently labeled anti‐CD11c, ‐CD86, and ‐CD80 antibodies for 30 min. Cells treated with pathway agonists were used as positive controls.

### 4.27 Enzyme‐linked Immunosorbent Assay of Cytokines and Biochemical Indexes in Animal Models In Vivo

Intratumor and serum levels of cytokines IFN‐γ (KMC4021, Invitrogen), TNF‐α (BMS607‐3, Invitrogen), IL‐2 (BMS601, Invitrogen), ANAs (EM1607, FineTest), and IL‐12 (p70) (BMS6004, Invitrogen) in mice were measured in vivo at the specified times post‐treatment. Serum was obtained from blood collected from the submandibular vein and centrifuged (2000 ×*g* in a refrigerated centrifuge at 4°C for 10 min). Supernatants were collected and stored at –80°C until cytokine levels were measured using the ELISA kit in accordance with the manufacturer's protocol. Biochemical analysis was performed on whole blood or serum and included routine factors of blood, liver, and kidney function.

### 4.28 Culture of BMDCs and Cross‐Presentation Assays

To obtain bone marrow, femurs and tibias from mice were cut at both ends, and the bone marrow was flushed into BMDC media by brief centrifugation at 10,000 ×*g*. RBCs were lysed with RBC lysis buffer (420301, BioLegend) for 1 min at room temperature. Tissue CD11c^+^ cells were enriched using CD11c Positive Selection Kit II (18780, STEMCELL). T cells were enriched using an EasySep Mouse Naïve CD8^+^ T‐cell Isolation Kit or a Naïve CD8a^+^ T‐cell isolation kit (19853, STEMCELL). Both tissues were mashed and passed through a 7 0‐µm filter.

For BMDC cultures, 5 × 10^6^ bone marrow cells from 8‐week‐old male or female BALB/c mice were resuspended in media supplemented with 5 ng/mL granulocyte macrophage‐colony stimulating factor, GM‐CSF (SRP3201, Merck), and 20 ng/mL Flt3‐L (SRP3198, Merck), then were seeded in a 10‐cm nontreated tissue culture plate. Cells were differentiated for 9 or 10 days, with the addition of 10 mL fresh media containing 5 ng/mL GM‐CSF and 20 ng/mL Flt3‐L on the fifth or sixth day, and 5 mL media containing 20 ng/mL GM‐CSF on the seventh day.

Cultured DCs were added to the lower chamber of a transwell dish containing RPMI‐1640. A mixture of azido‐HA and 4T1‐luc cells cultured in RPMI‐1640 was added to the upper chamber, followed by 200 µL logarithmically‐growing DBCO‐EcN‐pfo dispersed in DPBS at a density of 1 × 10^8^ CFU mL^−1^. The assembled infusion unit was incubated at 37°C. The upper chamber was exposed to three rounds of periodic NIR under the following conditions: 0.308 W/cm^2^ and 5 cm for 5 min ON; and 5 min OFF. After 2 h, DCs in the lower chamber were removed and incubated with naïve CD8^+^ T cells for 12 h. The cells were then collected and stained with antibodies against CD45^+^, CD3^+^, CD8^+^, and IFN‐γ^+^, and then analyzed by flow cytometry.

To assess cross‐presentation of pfo, EcN‐pfo‐HA (1 × 10^5^ CFU, upper chamber, laser treatment) was incubated with murine BMDCs (lower chamber) in a transwell (3450) at 37°C for 30 min (Figure S20D), and stained by pHrodo BioParticles, HA‐tag antibody, and Hoechst 33342 before being examined using SIM.

### 4.29 T‐cell Depletion

A mouse model of breast cancer was established as previously described in Figures S42A and S43A. To consume CD8^+^ or CD4^+^ T cells, 250 mg InVivoMAb anti‐mouse CD8α (BP0004‐1, BioXCell), anti‐mouse CD4α (BP0003‐1, BioXCell) or isotype control (BP0089, BioXCell) were intraperitoneally injected on days −1, 6, 15, and 24 of the courses of treatment. Images and parameters, including mouse body weight data, were obtained daily from the day of administration to the end of the study, at which time the mice were euthanized with carbon dioxide and the tumor tissue was analyzed by flow cytometry.

### 4.30 Saporin Assay

Saporin assay was performed as previously reported [71]. To monitor saporin‐mediated activation, BMDCs (3 × 10^5^/well) were seeded in 96‐well treated tissue culture U‐bottom plates. Cells were pulsed for 30 min at 37°C with saporin (S9896; Merck), washed once in PBS, spun at 380 ×*g* for 3 min, resuspended in the transwell lower chamber with DC media containing 0.01 mg mL^−1^ puromycin (A1113803, Gibco) (Figure S20E). EcN‐pfo (NIR treatment) was added to the upper chamber and incubated for 30 min at 37°C. The control was treated with no laser. Incorporation was determined by staining with an αPuromycin‐AF488 antibody (MABE343, Merck) in Wash buffer for 45 min on ice and analyzing the cells by flow cytometry.

### 4.31 Immune Memory Assessment of Prevention Tumor Recurrence

At 61 days post elimination of the primary 4T1‐luc tumor following treatment with STEPT, a second batch of 4T1‐luc cells (1 × 10^6^) was again orthotopically injected into the same mice. A control group (G5) of blank mice was also treated with orthogonal in situ injection of DBCO‐EcN‐pfo (1 × 10^8^ cells/mL in 40 µL), using the same intratumor loading strategy (Figure 7A). Tumor size was then closely recorded. Bioluminescence imaging was used to visualize antitumor effects. At day 93, tumor tissue and spleen were harvested and homogenized into a single‐cell suspension, which was then analyzed by flow cytometry. Details of the antibody panels used for spectral flow cytometry analysis are listed in Table S5. To analyze the percentage of memory T cells, spleens collected from different groups of mice were incubated with the antibodies listed in Table S5. Flow cytometry was used to analyze the percentages of central memory T cells (CD3^+^ CD8^+^ CD62L^+^ CD44^+^) cells and effector memory T cells (CD3^+^ CD8^+^ CD62L^−^ CD44^+^).

Harvested tumor tissues were also homogenized in sterile PBS, and the residual bacteria were collected by centrifugation, then diluted and cultured on agarose medium for 12 h. The number of bacterial colonies was obtained using a colony counter.

### 4.32 Distant Immune Memory Assessment of Tumor Suppression

The first tumor inoculation, 4T1‐luc cells (1 × 10^6^) were injected orthotopically into the left mammary fat pad of each mouse (day −8). A second tumor inoculation was performed 2 days later (day −6), with orthotopic injection of 4T1‐luc cells (1 × 10^6^) into the right mammary fat pad of each mouse (Figure 7H). The mice were then randomly divided into two groups. The primary tumors were initiated with STEPT on day 0, and the size of distant tumors was measured. Large doses of DBCO‐EcN‐pfo (1 × 10^8^ cells/mL, 40 µL) were injected into the tumor of the control group (G5). Bioluminescence imaging was used to visualize antitumor effects. Tumor tissue was harvested 20 days after treatment and homogenized into a single‐cell suspension, which was then analyzed by flow cytometry. Harvested tumor tissues were also homogenized in sterile PBS, and the residual bacteria were collected by centrifugation, then diluted and cultured on agarose medium for 12 h. The number of bacterial colonies was obtained using a colony counter.

### 4.33 Statistical Analysis

For quantitative analyses, a minimum of three biological replicates was analyzed. ^1^H NMR, FT‐NIR, GPC‐MALS, SIM experiments, and H&E experiments were repeated three times. FlowJo (version 10.0.0.0) was used to analyze the flow cytometry data. GraphPad Prism (version 8.3.0.538) was used for all other statistical analyses. Data are expressed as the mean ± standard error of the mean (SEM) unless otherwise indicated. Comparisons of two groups were performed using unpaired or paired two‐tailed Student's *t*‐tests. For multiple comparisons, one‐way analysis of variance with a Tukey's post‐hoc test was used when more than two groups were analyzed, and two‐way ANOVA with a Bonferroni post‐hoc test was used when two parameters were considered. A *P* value <0.05 was considered to indicate significance.

## Author Contributions

Conceptualization: Ju‐Zheng Sheng. Methodology: Yu‐Jia Wang, Wen‐Jie Jiang, Hua‐Jun Zhao, Xiao‐Lin Meng, and Feng‐Shan Wang. Animal experiments: Yu‐Jia Wang, Jian‐Qun Deng, Yi‐Min Cai, and Yi Li. Cell experiments: Yu‐Jia Wang, Wen‐Jie Jiang, and Jin Hou. Writing: Ju‐Zheng Sheng and Yu‐Jia Wang.

## Funding

This work was supported in part by the National Key R&D Program of China (2023YFA0914300), the Projects of Science and Technology Department of Shandong Province (Project no. 2022SFGC0104 to JS), Projects of Science and Technology Department of Jinan (Project no. 202323011) and Open Projects Fund of NMPA Key Laboratory for Quality Research and Evaluation of Carbohydrate‐based Medicine and Shandong Key Laboratory of Carbohydrate Chemistry and Glycobiology.

## Conflicts of Interest

One patent has been published relating to this article. The authors declare no conflicts of interest.

## Supporting information



Supporting information

Supporting information

## Data Availability

All data supporting the results are available in the main text or the Supporting Information. Additional data are available from the corresponding author upon reasonable request. *E. coli* Nissle 1917 genome sequences and other sequences were obtained from the National Center of Biotechnology Information under accession code NZ_CP022686.1, NZ_CP075979.1, GenBank: CCQ07420.1, and addgene: Plasmid #192473. All data are available in the main text or the supplementary material. Source data are provided with this paper.
