# Supplementary material for: A Bioorthogonal and Programmable Bacterial Delivery System for Spatiotemporally Targeted Therapy of Solid Tumors
Source: Exploration (Beijing). 2025 Dec 18;5(6):20240396. doi: 10.1002/EXP.20240396 (PMC12752581; doi:10.1002/EXP.20240396)
Supplement: Supplementary file 1 — Supporting information [file EXP2-5-20240396-s001.pdf]

Supporting Information for

A bioorthogonal and programmable bacterial delivery system for spatiotemporally targeted therapy of solid tumors

Yu-Jia Wang[1,5], Wen-Jie Jiang[1,2], Hua-Jun Zhao[3], Jian-Qun Deng[1], Yi-Min Cai[1], Yi Li[1], Xiao-Lin Meng[1], Jin Hou[4], Feng-Shan Wang[1,2], Ju-Zheng Sheng[1,2*]

[1]Key Laboratory of Chemical Biology of Natural Products (Ministry of Education), School of Pharmaceutical Sciences, Cheeloo College of Medicine, Shandong University; Jinan 250012, China.

[2]National Glycoengineering Research Center, Shandong University; Jinan 250012, China.

[3]Institute of Immunopharmaceutical Sciences, School of Pharmaceutical Sciences, Cheeloo College of Medicine, Shandong University; Jinan 250012, China.

[4]The State Key Laboratory of Microbial Technology, Shandong University; Qingdao, 266000, China.

[5]Shandong Provincial Hospital Affiliated to Shandong First Medical University, Jinan, Shandong, 250021, China.

*Ju-Zheng Sheng

Email: shengjuzheng@sdu.edu.cn

This PDF file includes:

Figures S1 to S50

Tables S1 to S5

SI References

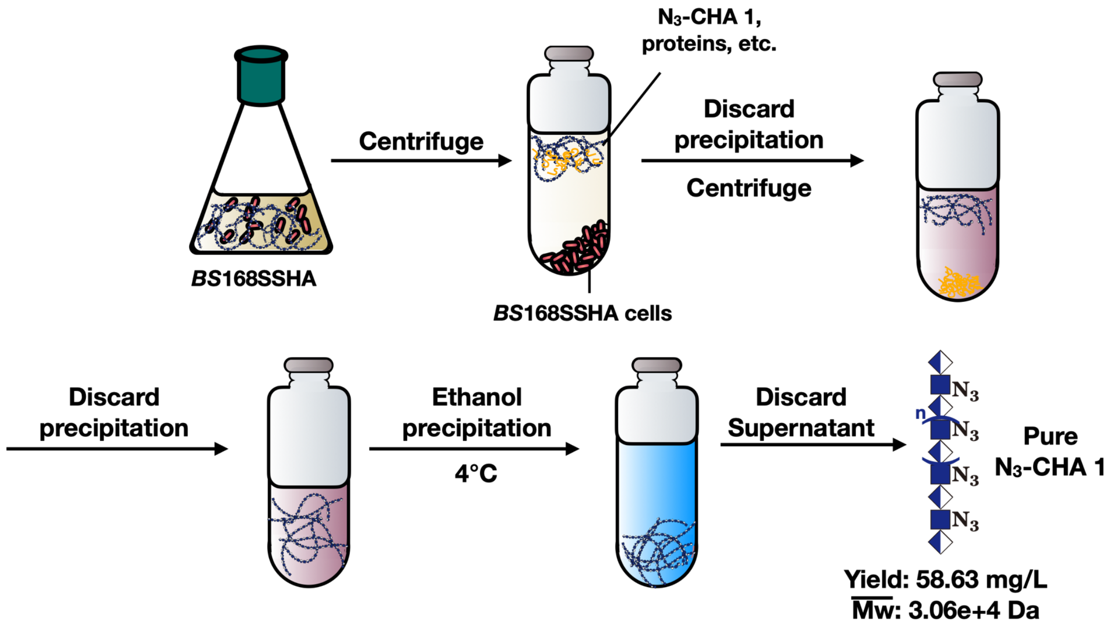

Figure S1.

Purification of bioengineered azido-HA from supernatants of fermentation cultures. To produce azido-HA polysaccharides, *B. subtilis* 168SSHA was grown in extracellular GlcNAc-supplemented medium; cells reaching a certain growth phase were then harvested, rinsed, and further grown in medium containing GlcNAz and simultaneously induced for szHasA expression. The polysaccharides were purified from the supernatant of the culture medium after fermentation. The yield and average molecular weight (MW) of the bioengineered azido-HA, as determined by HPLC-MS and gel permeation chromatography with multi angle light scattering (GPC-MALS), respectively, were ~58.63 mg/L and 30.6 kDa.

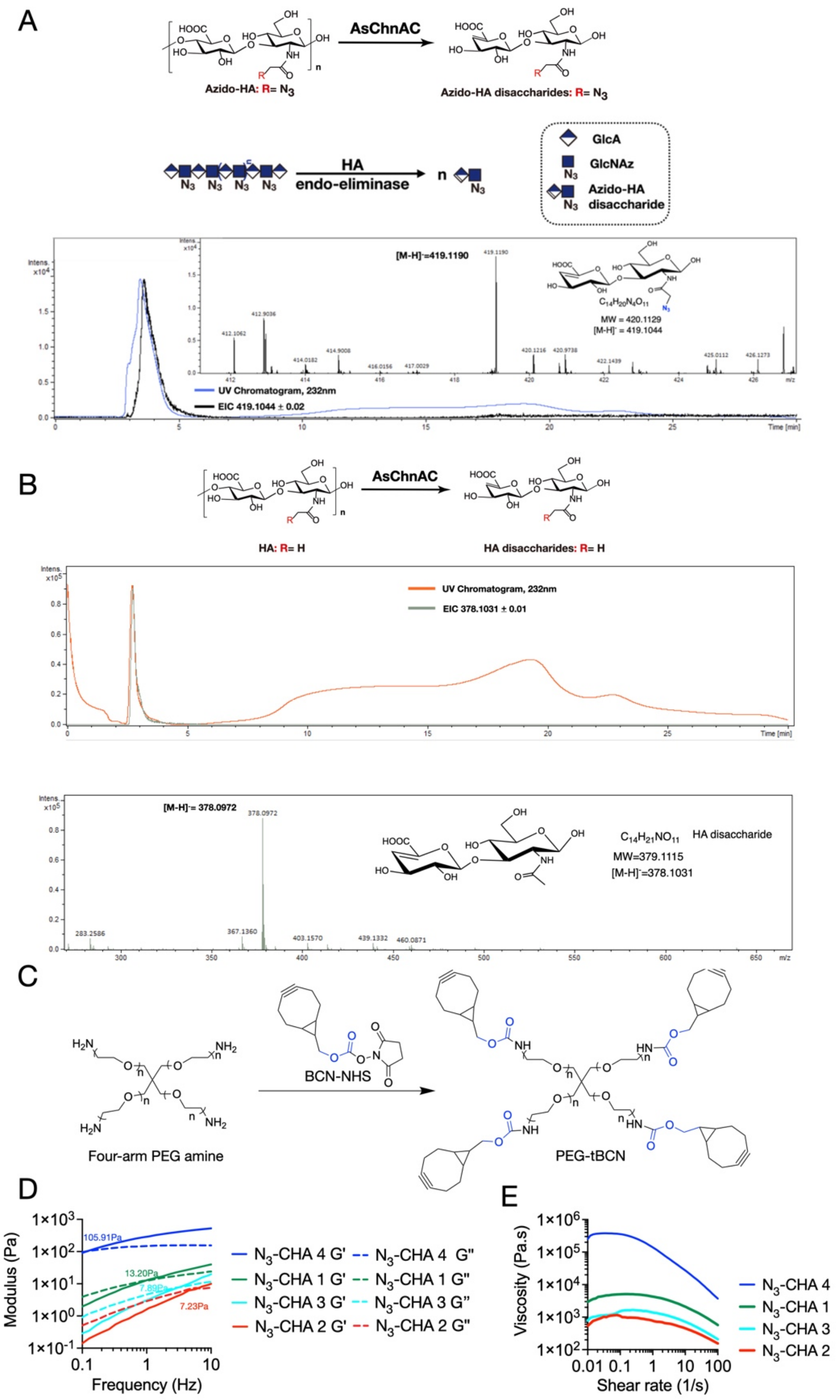

Figure S2.

Analysis and crosslinking of azido-HA. (**A and B**) To determine the production of azido-HA, the purified polysaccharides were digested with chondroitinase AC exolyase (AsChAC)[1], an endo-β-eliminase that tolerates azido-HA as a substrate. The degraded

products were analyzed by high-performance liquid chromatography combined with mass spectrometry (HPLC-MS). As expected, the Δ4-unsaturated disaccharide ΔUA-GlcNAz was observed, demonstrating that azido-HA had been synthesized by *B. subtilis* 168SSHA and secreted into the medium. Our data also suggested that UDP-GlcNAz is a suitable donor substrate for the β1-3 *N*-acetylglucosaminyltransferase activity of szHasA. Briefly, HPLC-MS analysis of disaccharides from azido-HA (**A**) and HA (**B**) after enzymatic hydrolysis of the polysaccharide at 232 nm, showing incorporation of azido groups into the disaccharide repeat units of bioengineered azido-HA (**A**) and incorporation of hydrogen groups into the disaccharide repeat units of bioengineered HA (**B**). After degradation by AsChAC, azido-disaccharides and HA disaccharides were identified by HPLC-MS. Experiments were performed twice, with two biological replicates each time. (**C to E**) To improve the retention ability of our bioengineered azido-HA, we used crosslinker to create an injectable hydrogel by PEG-tBCN (four-arm-Polyethylene Glycol amine-tetrad-Bicyclononyne)[2] (Figure 1E). Combining favorable viscosity and injectability with the goal of maximizing azide group retention led to an optimal azido-HA: crosslinker ratio of 10:1 (see for more, Figure S1 and S2, Tables S3 and S4). (**C**) Crosslinking agent PEG-tBCN synthesized by four-arm PEG amine and BCN-NHS. BCN-NHS is a compact, hydrophilic clickable molecule, which was reacted with a four-arm polyethylene glycol (PEG) amine in one step to produce PEG-tBCN, a tetramer crosslinker with four cyclooctyne groups. Azido-HA was crosslinked with PEG-tBCN via copper-free click chemistry (Figure 1F). (**D** and **E**) To meet the viscosity and injection requirements for a hydrogel while maximizing azide group retention on HA, we explored different ratios of azido-HA:PEG-tBCN. (**D**) As the frequency increased, the

storage modulus (G') exceeded the loss modulus (G"), indicating hydrogel formation (Table S3). Rheological traces of the different hydrogel formulations. The values shown represent the modulus value of the intersection point. Four ratios were designed to obtain the optimum crosslinking conditions for hydrogel performance: N_3-CHA 1 (PEG-tBCN = 1, azido-HA = 10); N_3-CHA 2 (PEG-tBCN = 1, azido-HA = 15); N_3-CHA 3 (PEG-tBCN = 1, azido-HA = 12); and N_3-CHA 4 (PEG-tBCN = 1, azido-HA = 5). (**E**) Changes in shear viscosity of the different hydrogel formulations at the increased shear rate. When the shear rate was increased from 0.01 s^{-1} to 100 s^{-1}, the viscosity decreased significantly, giving the hydrogel shear-thinning properties indicative of good injectability.

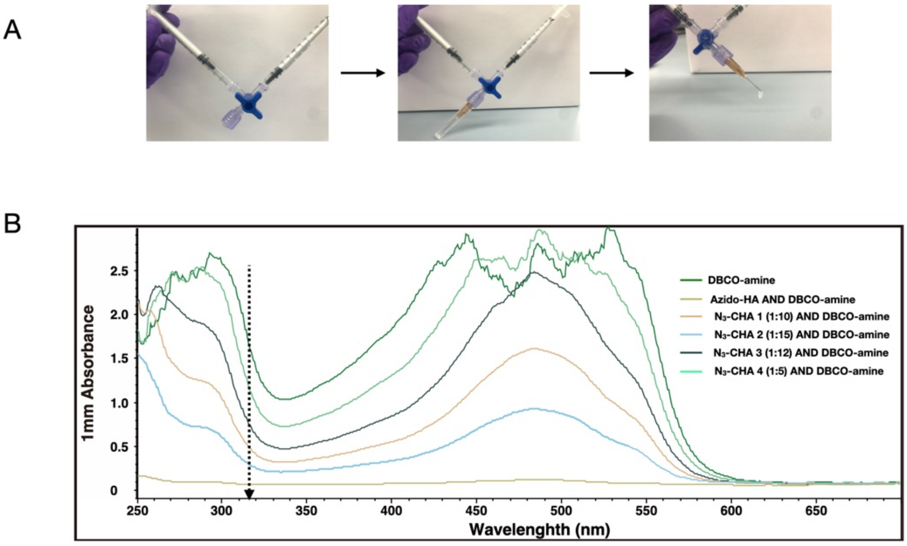

Hydrogel	Azido-HA	Cross-linked Azido-HA(1:15)	Cross-linked Azido-HA(1:12)	Cross-linked Azido-HA(1:10)	Cross-linked Azido-HA(1:5)
Azide (DS)	593	468	124	182	62

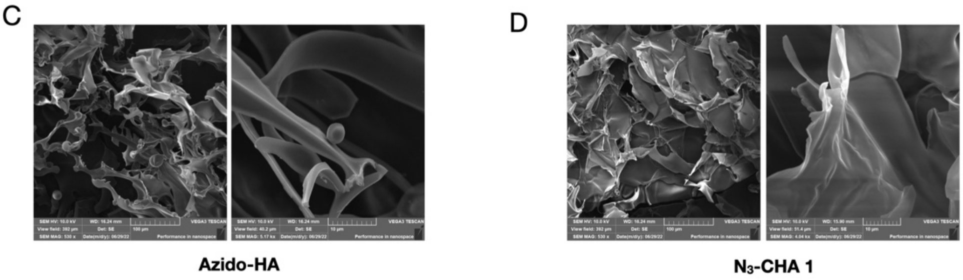

Figure S3.

(**A**) Diagram of crosslinking of azido-HA and injectable analysis of its hydrogels. (**B**) Decrease in the azide groups of azido-HA before and after crosslinking, based on the decrease in DBCO absorption at 308 nm following incubation of azido-HA with excess DBCO; n = 3 biologically independent samples. For detailed steps, see Methods. (**C** and **D**) Characterization of the novel azido-HA and N_3-CHA 1. Scanning electron microscopy images of azido-HA hydrogels before (**C**) and after (**D**) crosslinking. Scale bars: 100 µm (left) and 10 µm [3].

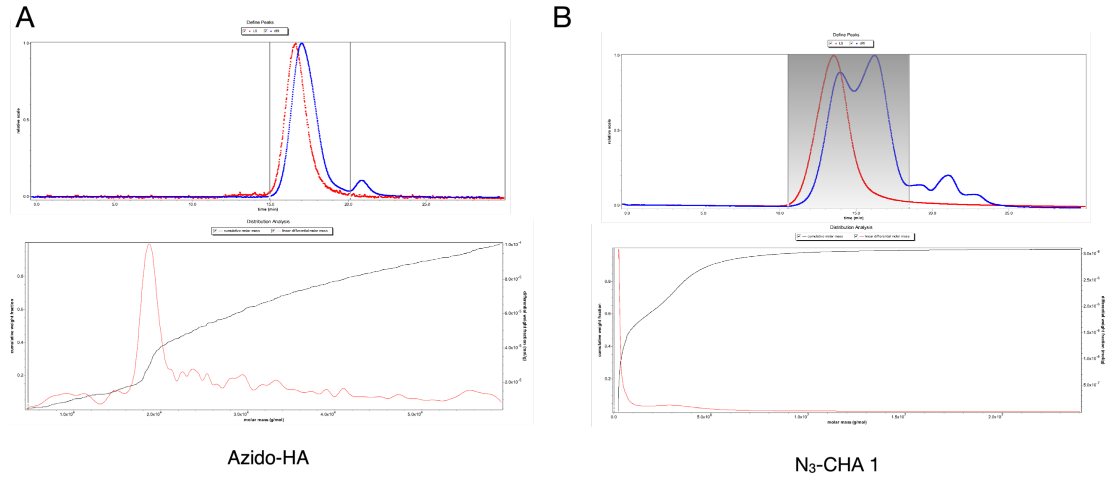

Figure S4.

Determination of weight average molecular mass (Mw) of azido-HA hydrogels before (**A**) and after cross-linking (**B**) by gel permeation chromatography with multi-angle laser light scattering combined with size exclusion chromatography (GPC-MALS-SEC) were 30.6 kDa and 223.3 kDa; n = 3 biologically independent samples.

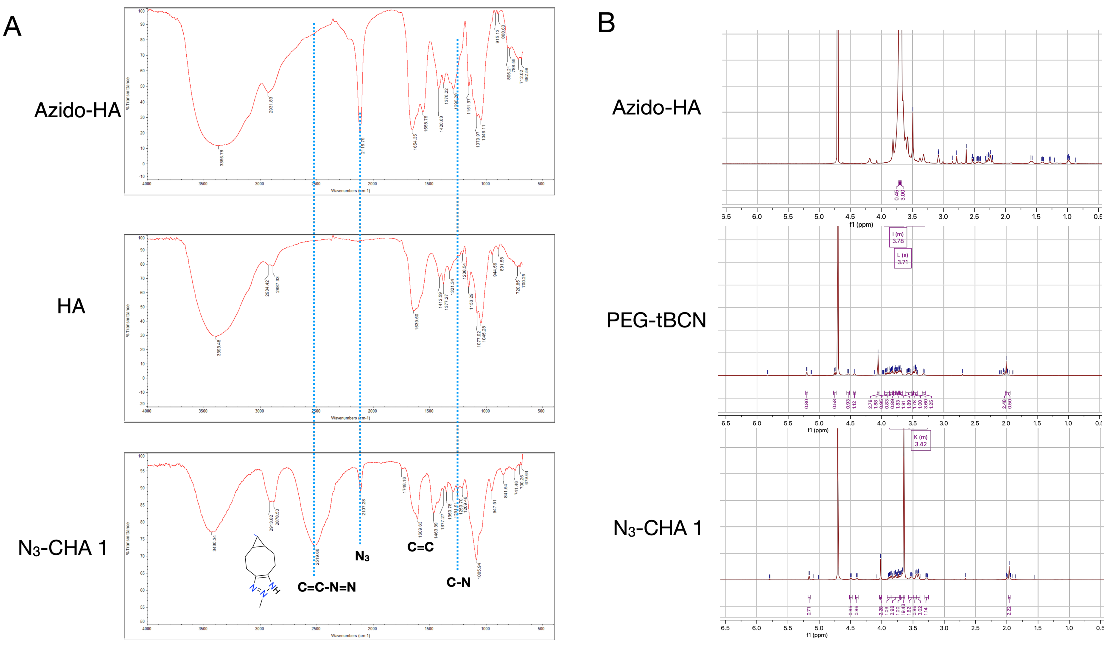

Figure S5.

Structural analysis proved that azido-HA crosslinked successfully. (**A**) Fourier transform-infrared spectra of azido-HA, HA, and N_3-CHA 1. As shown in the Fourier transform-infrared spectrum analysis, the peak stretching value of the azido-group at 2100 cm^{-1} to 2120 cm^{-1} was lower in N_3-CHA 1 compared with that in non-crosslinked azido-HA, signifying a decrease in the azide group retention rate after crosslinking. (**B**) ^{1}H-NMR spectra of azido-HA, PEG-tBCN, and N_3-CHA 1. The results of nuclear magnetic resonance (NMR) also demonstrated the successful crosslinking. Data represent 3 biologically independent samples.

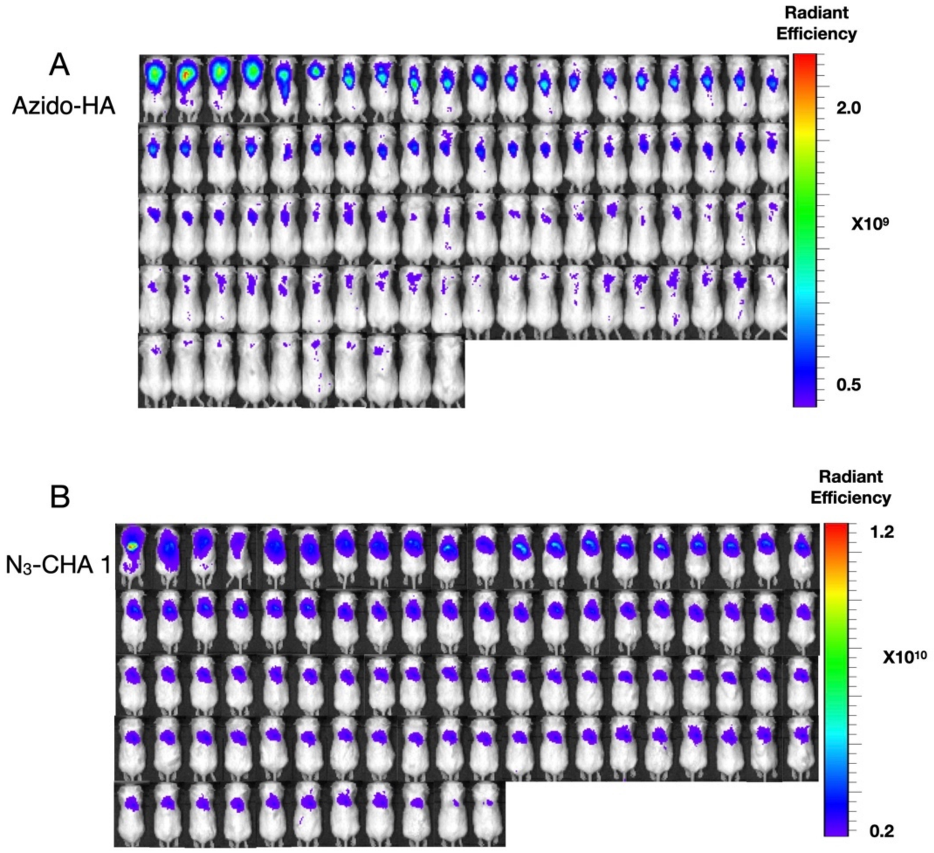

Figure S6.

IVIS imaging of mice treated with a 100-μL shoulder S.C. injection of uncrosslinked azido-HA (1 mg/mL in PBS; **A**) or crosslinked N_3-CHA1 (1 mg/mL in PBS; **B**) hydrogel modified with the fluorophore DBCO-Cy5 to allow for in vivo detection. Hydrogel residency was assessed from in vivo changes in hydrogels from the 1st day to the 120th day using the IVIS. Signals were quantified as total radiant efficiency at the injection site in Figure 1H. The percentage of biopolymer remaining was determined by fluorescence radiant efficiency at the injection site. A fluorescent signal remained evident at the biopolymer injection site for approximately 4 months; n = 3 biologically independent mice.

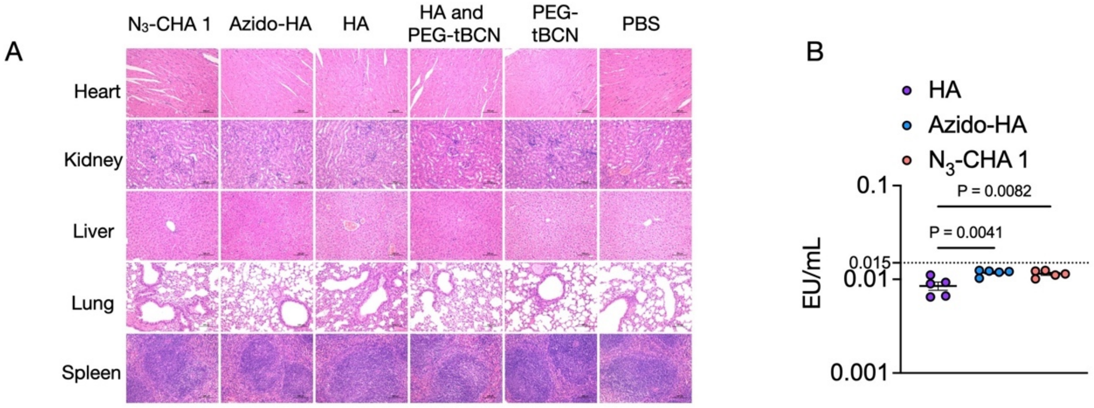

Figure S7.

(**A**) Representative images of histological assessment of inflammation in mice treated with a 100-μL S.C. injection of azido-HA (1 mg/mL in PBS), HA (1 mg/mL in PBS) or N_3-CHA1 (1 mg/mL in PBS) hydrogel. Mice were sacrificed after day 140, major organs (heart, liver, kidneys, lung, and spleen) were removed, embedded in paraffin, sectioned, and stained with H&E; N_3-CHA 1 did not induce an inflammatory response. Scale bar: 100 μm; n = 3 biologically independent mice. (**B**) Limulus amebocyte lysate assay of bacterial endotoxin of HA, azido-HA, and N_3-CHA 1. It showed that N_3-CHA 1 had no difference in bacterial endotoxin compared with standard HA. Data expressed as mean ± standard deviation; n = 5 biologically independent experiments. Statistical significance determined by one-way ANOVA with Dunnett's post-hoc test (**B**).

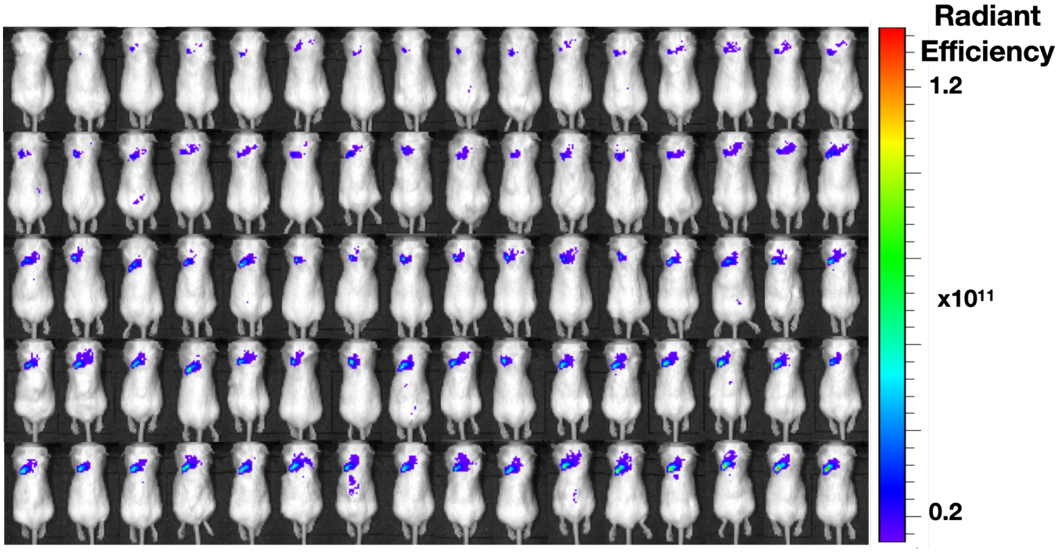

Figure S8.

Repeated replenishment of the N_3-CHA1 hydrogel reservoir by DBCO-Cy5. Female mice received a 100-µL left shoulder S.C. injection of N_3-CHA 1 hydrogel (1 mg/mL in PBS) and a 100-µL right shoulder S.C. injection of HA (1 mg/mL in PBS). Each mouse was given DBCO-Cy5 (10 µL in 5 mM DMSO) intravenously every 2 days. Imaging was performed 24 h after each injection. The entire replenishment process was repeated 80 times. Fluorescence imaging was conducted using 646-nm Ex and 662-nm Em filters to visualize Cy5, and the signals were quantified as total radiant efficiency of the biopolymer injection site in Figure 1J; n = 3 biologically independent mice.

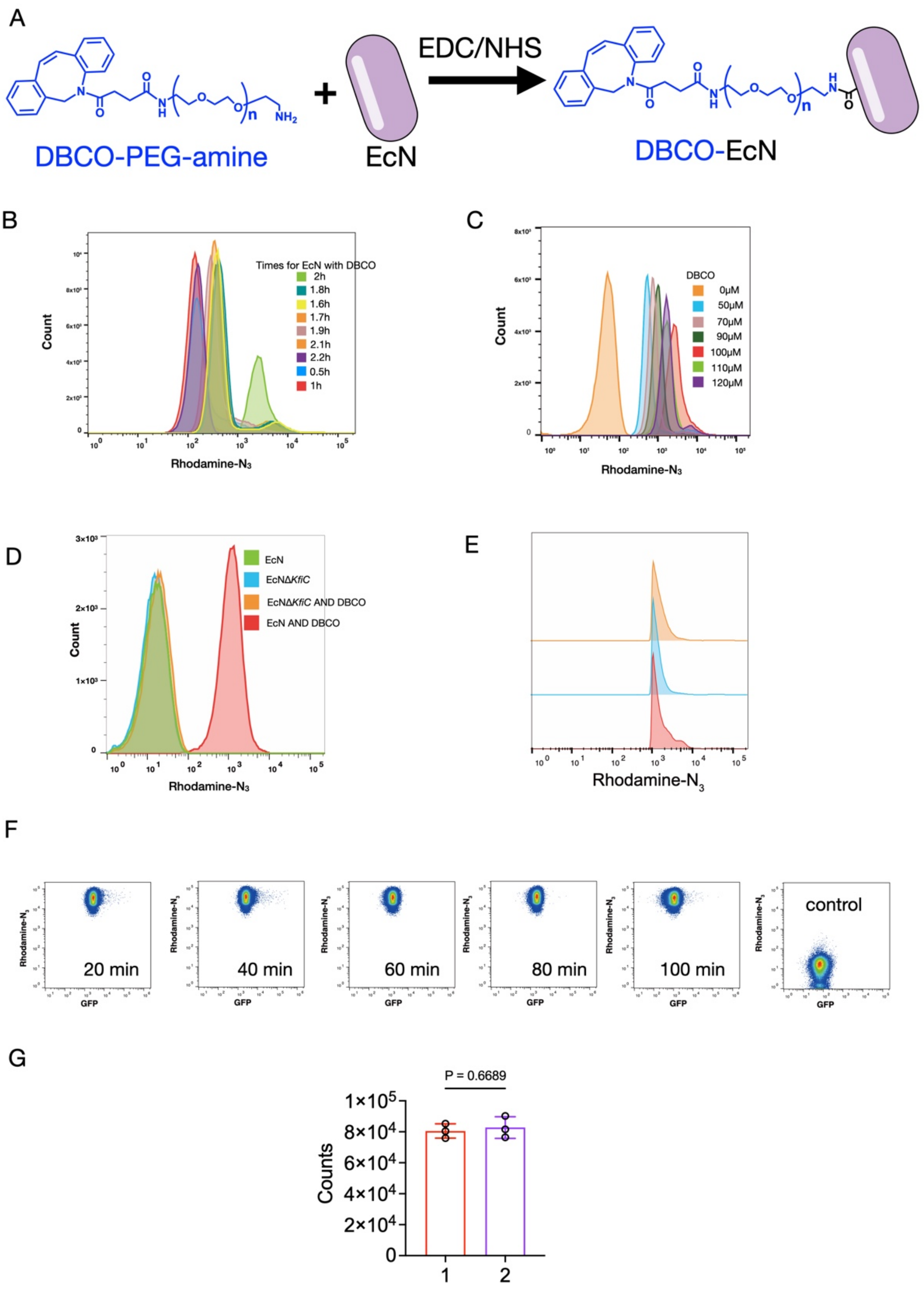

Figure S9.

(**A**) Schematic diagram of DBCO labeling of EcN. (**B** and **C**) Representative flow cytometry histograms and quantitative analysis of DBCO-labelled in different times (**B**) and DBCO-labelled in different concentrations (**C**) with EcN cells. Evaluations of labeling were

performed at different time points; n = 3 biologically independent samples. (**D**) Representative flow cytometry histogram of data analyzed in Figure 2B; n = 3 biologically independent samples. (**E**) EcN activation to flow cytometry was performed once a day for three consecutive days. Flow cytometry results showed that DBCO labeling of three batches of EcN was consistent, and the labeling efficiency was 100 %. (**F**) In order to determine the effective DBCO labeling maintenance time, unlabeled EcN were divided into 6 groups, with 1 group as control and the other 5 groups as DBCO labeling. One group was selected every 20 minutes for fluorescein Rhodamine-N_3 reaction, and then flow cytometry analysis was performed. The results showed that the DBCO labeling of EcN remained effective for at least 100 minutes. (**G**) Equal amounts of EcN-GFP and EcNΔ*KfiC*-GFP are mixed together for DBCO labeling in Figure 2C. After the labeled cells react with fluorescein Rhodamine-N_3, the number of cells in clusters (1 and 2) are counted by flow cytometry analysis. Data in **G** expressed as the mean ± SEM. P values determined by unpaired two-tailed Student's t-test (**G**).

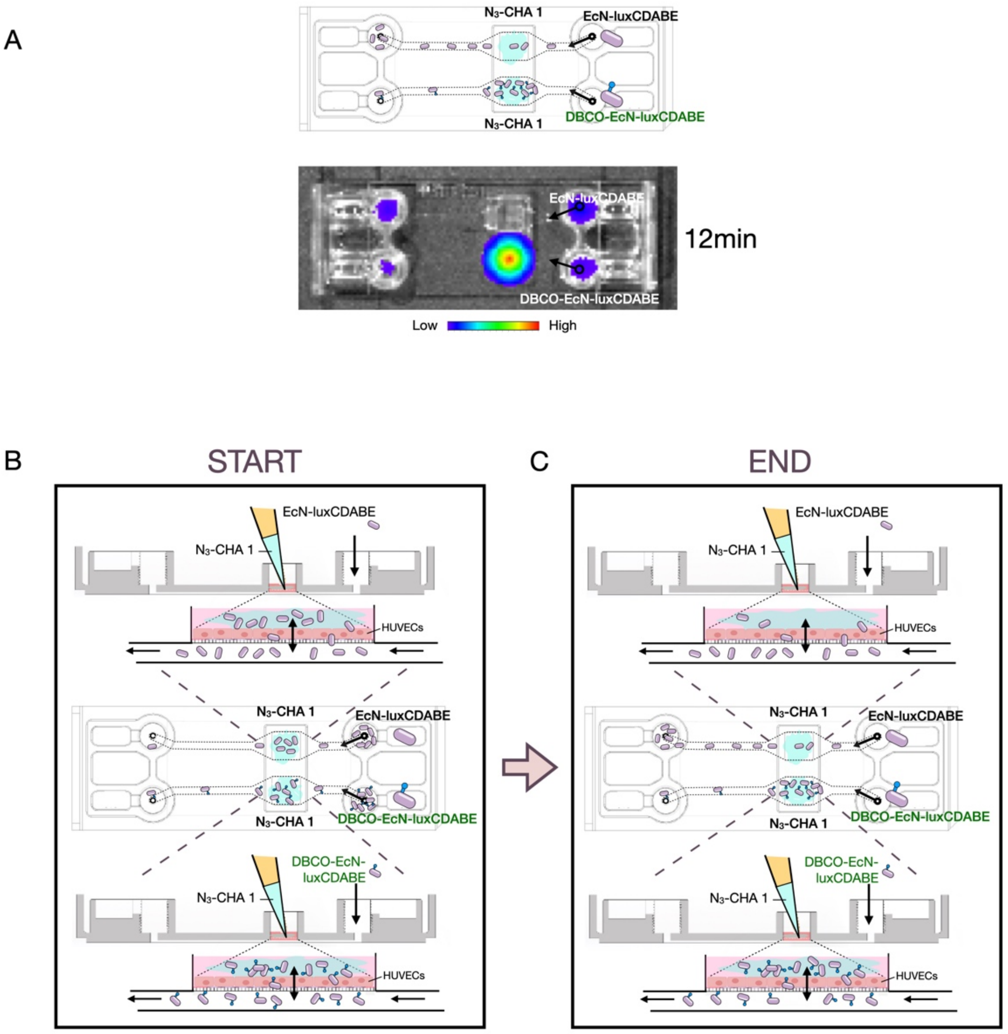

Figure S10.

(**A**) Schematic diagram of the microfluidic chip experiment. (**B**) Schematic diagram at the beginning of the microfluidic experiment. (**C**) Schematic diagram at the end of the microfluidic experiment.

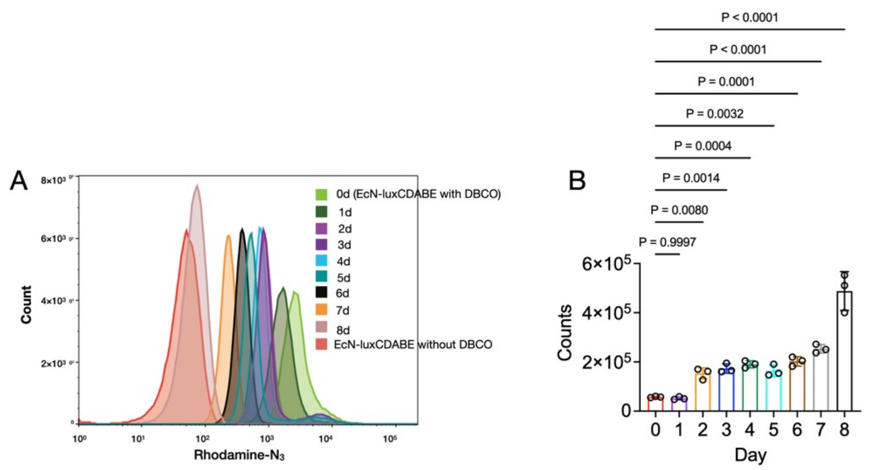

Figure S11.

(**A**) Flow cytometry measurement of the number of DBCO-EcN cells in tumors of fibrosarcoma model mice established by S.C. injection of WEHI164 cells, intratumor injection of azido-HA, and 1 h later intravenous injection of DBCO-EcN-GFP. Unlabelled EcN was used as a control. Day 0 refers to DBCO-labelled EcN before intravenous injection. After DBCO-EcN-GFP treatment, in order to evaluate the DBCO retention of bacteria in each group of tumors at a specific time point on day 1-8, the tumors were dissected and the bacteria in the suspension were labeled with Rhodamine-N_3. DBCO-EcN-GFP that colonized the tumor site was quantified. Although the fluorescence intensity gradually decreased, the number of clickable bacteria increased. (**B**) The number of DBCO labelled EcN-GFP in tumor was quantitatively analyzed. Flow cytometry results showed that some DBCO modifications remained on the surface of progeny EcN cells for 8 days *in vivo*. n = 3 biologically independent mice. Data in **B** expressed as the mean ± SEM. P values determined by one-way ANOVA with Tukey's post-hoc test (**B**).

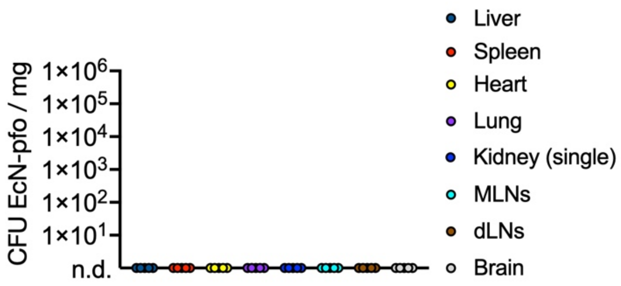

Figure S12.

We monitored the distribution of bacteria in non-target organs (spleen, heart, lung, kidney, draining lymph node, mesenteric lymph nodes and brain) within 40 days after administration, and the results showed that there was no off-target effect in organs during the 40-day detection. dLNs, draining lymph node; MLNs, mesenteric lymph nodes. n = 5 biologically independent mice.

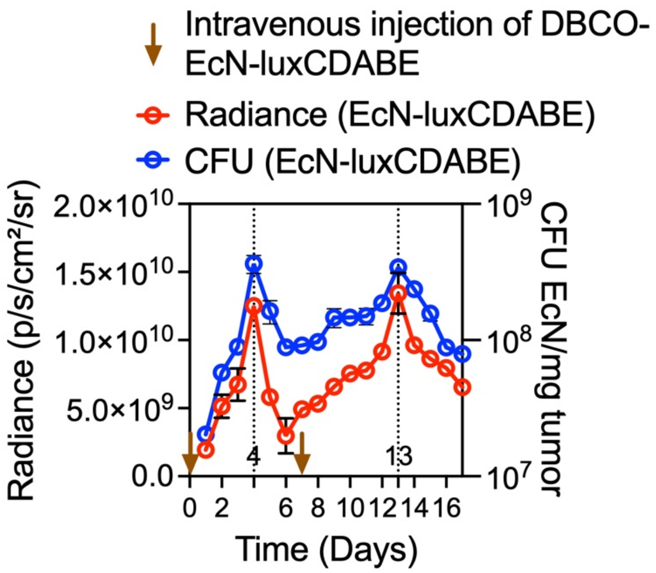

Figure S13.

Colonization of EcN-luxCDABE after spatiotemporal localization in a unilateral tumor model in mice *in vivo*. Bioluminescence changes in the tumor load and CFU of EcN-luxCDABE, which peaked on the 4th day after the first EcN injection; Then, the second intravenous injection was performed on the 7th day, and the amount peaked again on the 13th day (on the 6th day after the second bacterial injection). This determines the moment of NIR in tumor therapy. n = 3 biologically independent mice.

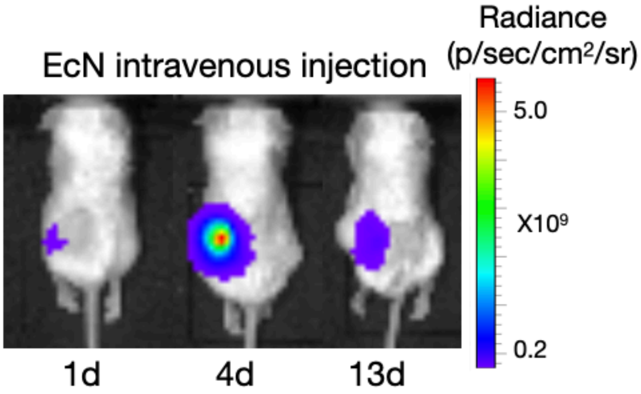

Figure S14.

Comparison between direct intratumor (i.t.) (**Figure 2M**) and intravenous (i.v.) (**Figure S14**) injection of DBCO-EcN-luxCDABE in a unilateral tumor model. Bioluminescence imaging showing the distribution of EcN-luxCDABE (i.v.).

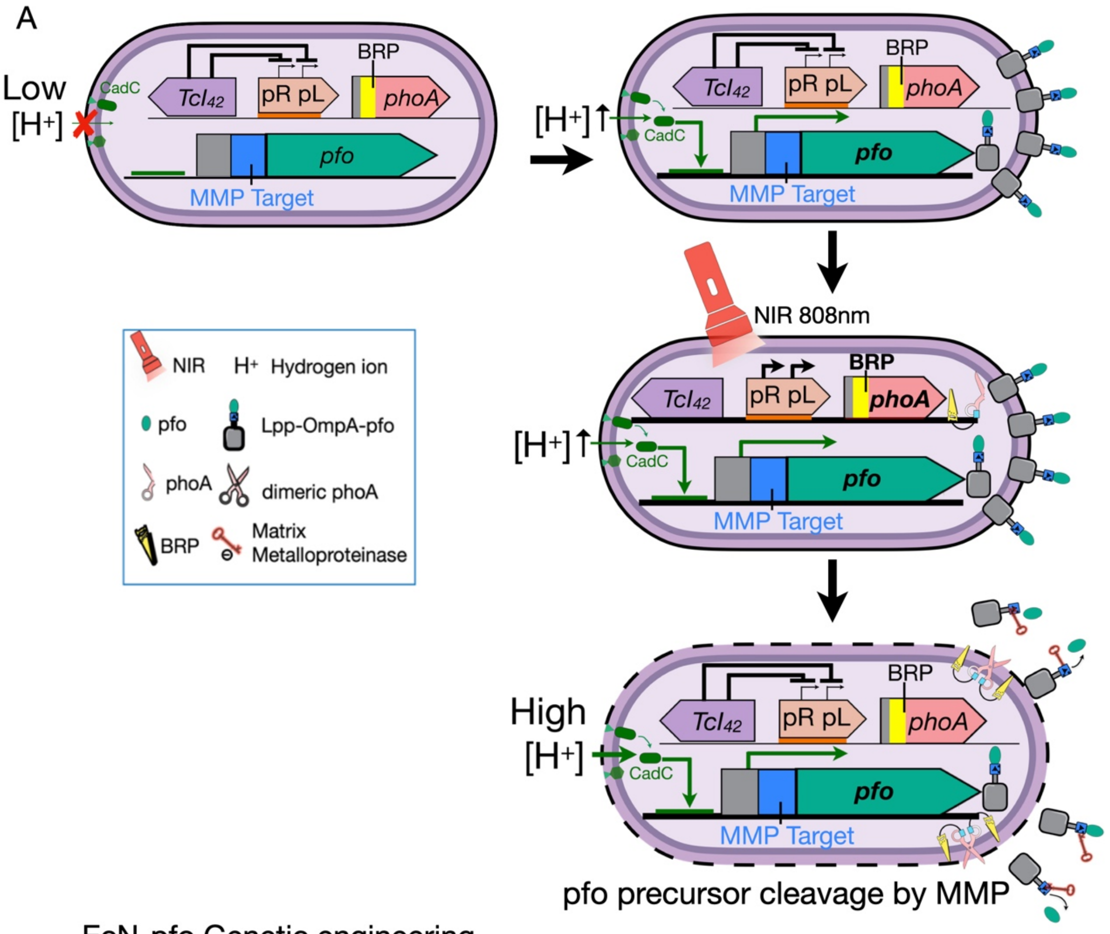

EcN-pfo Genetic engineering

B

For_DBCO-EcN-pfo
Command 1: IF Environment = N_3-CHA 1 = Cancer_Cells THEN
EXCUTE Click_Reaction
EXCUTE Colonization_in_tumor
Command 2: IF Environment = Cancer_Cells = H^+_concentration_increases THEN
EXCUTE Produce_precursor_pfo
Command 3: IF Environment = Cancer_Cells_with_NIR =
Temperature_of_tumor_is_42_°C THEN
EXCUTE Produce_BRP_and_phoA
EXCUTE BRP_and_phoA enhance the permeability of bacterial outer membrane
EXCUTE Release_precursor_pfo
EXCUTE Matrix_Metalloproteinase_cleave_precursor_pfo
EXCUTE Release_pfo
EXCUTE Command_3_three_times
EXCUTE H^+_concentration_increases in_EcN-pfo
EXCUTE Command_2

Figure S15.

Design of the STEPT strategy for EcN. **(A)** Based on the spatiotemporal targeting system, we designed bacterial vectors expressing the cytotoxic molecule pfo (Perfringolysin O). Pfo is the toxin from *Clostridium perfringens*[4]. The expression of cytotoxic genes can

be controlled more precisely by using inducible promoters to avoid toxicity to normal tissues. The EcN-pfo strain was engineered to carry pfo precursor proteins under the control of promoter pCadC, which is regulated by a membrane-tethered activator protein (CadC) that exhibits increased activity in medium with an acidic pH compared with a neutral pH. EcN-pfo can induce the expression of pfo precursor proteins with matrix metalloproteinase (MMP) cleavage sites under acidic conditions (pH = 6.8) N_3-CHA 1 helps DBCO-EcN-pfo to colonize in solid tumors and activates the expression of pfo precursor proteins with MMP cutting sites in the acidic environment of tumors (pH = 6.8), so that pfo precursors are distributed in the periplasmic space and outer membrane (OM) of bacteria. pL and pR are λ-derived promoters of phage, and the promoters pL and pR are inhibited by temperature-sensitive Tcl_{42} inhibitors expressed from the same vector, while temperature transfer to 42 °C rapidly results in the inactivation of Tcl_{42} inhibitors[5]. Thus, phosphatase (phoA) and bactericin-releasing protein (BRP)[6] downstream of pL and pR promoters is activated under 42 °C. On the 4th day, NIR was applied to the tumor, raising the local tumor temperature to 42 ° C, triggering phoA and BRP expression. The insertion of BRP, a lipoprotein with a stable signal sequence, into the outer membrane OM disrupts the local symmetry of OM, resulting in increased permeability of the lipid bilayer. By hydrolyzing the bacterial outer membrane with phoA, the disturbance of phoA and BRP-mediated OM is sufficient to cause the release of payloads. So, as proteins associated with the release of bacteriocins[7,8], phoA and BRP increase the permeability of the bacterial outer membrane and jointly promote the release of pfo precursors into the tumor microenvironment. The pfo precursors are cut by matrix metalloproteinases (MMP) to release pfo. Free pfo binds to cholesterol on tumor cell membrane, and pfo monomers aggregate into polymers to form a perforating effect, which directly leads to tumor cell death and potent tumor inhibition together with shrinkage and crusting. What's BRP increased the disturbance of the EcN outer membrane, upregulating the concentration of bacterial intracellular H ion and producing more pfo precursors. Gene illustrations are approximations and are not drawn to scale. (**B**) The method of gene regulation was summarized into programming strategy.

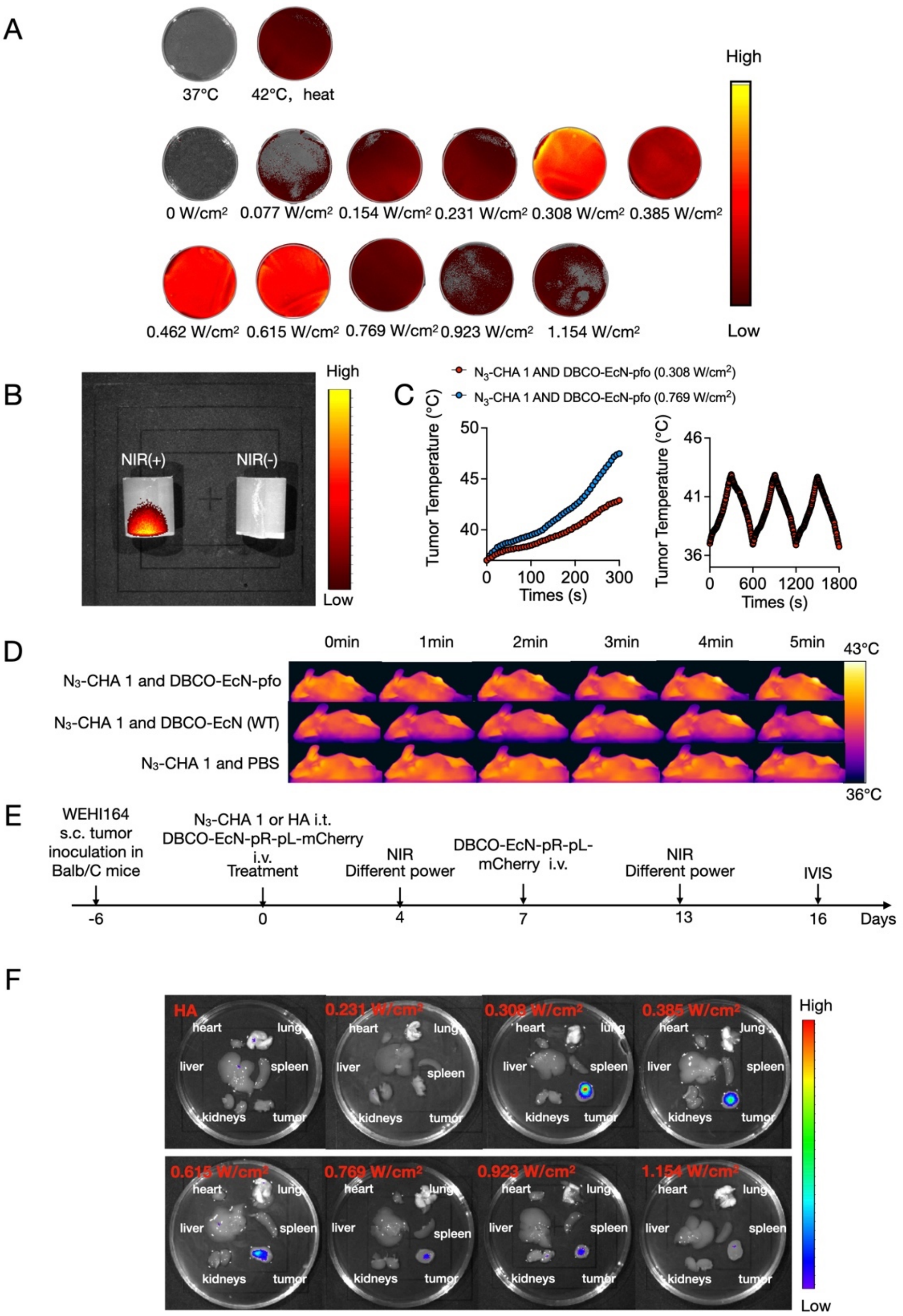

Figure S16.

Optimization of NIR parameters using the pBV220-pR-pL-mCherry plasmid construct in EcN. To explore the NIR conditions, we constructed the EcN-pR-pL-mCherry strain, which carries the mCherry as reporter under the temperature-sensitive promoter pR-pL. (**A**)

Fluorescence imaging of mCherry protein expression in EcN at 37 °C and 42 °C, and under a range of NIR power densities. The following conditions led to optimized in vivo expression of the EcN temperature-sensitive mCherry gene: three rounds of periodic irradiation with 0.308 W/cm^2 at 5 cm for 5 min ON and 5 min OFF. (**B**) Fluorescence imaging of EcN-pR-pL-mCherry in the center of the gel phantom under NIR. (**C**) The left panel shows the temperature change curves in tumors under NIR irradiation at 808 nm with different power densities (0.308 W/cm^2 or 0.769 W/cm^2). The right panel shows the photothermal stability of tumors under 808 nm laser irradiation with 0.308 W/cm^2. Experiments were independently performed three times, with similar results. (**D**) Representative infrared thermal images of tumors irradiated by NIR of 0.308 W/cm^2 at 5 cm for 5 min ON and 5 min OFF in different groups. Fibrosarcoma model Balb/C mice established by S.C. injection of WEHI164 cells. Tumors that reached approximately 100 mm^3 received intratumor injection of N_3-CHA 1, 1 hour later, intravenous injection of DBCO-EcN-pfo (5 × 10^7 CFUs), DBCO-EcN (WT), or PBS. On day 4 post treatment, NIR (0.308 W/cm^2) was administered. (**E**) Timeline of STEPT treatment, NIR, and fluorescence imaging of fibrosarcoma model mice established by S.C. injection of WEHI164 cells. Tumors that reached approximately 100 mm^3 received intratumor injection of azido-HA or HA, intravenous injection of DBCO-EcN-pR-pL-mCherry (5 × 10^7 CFUs). On day 4 post treatment, NIR (0.231 W/cm^2, 0.308 W/cm^2, 0.385 W/cm^2, 0.615 W/cm^2, 0.769 W/cm^2, 0.923 W/cm^2, or 1.154 W/cm^2) was administered. On day 7, intravenous injection of DBCO-EcN-pR-pL-mCherry (5 × 10^7 CFU) was repeated. On day 13, NIR was administered at the same power density as day 4, and on day 13, tumors and

organs were dissected for fluorescence imaging. The fluorescence intensity of mCherry was considered to indicate the expression strength in EcN under different NIR conditions. (**F**) Fluorescence imaging of mice injected with 5×10^7 CFUs of DBCO-EcN-pR-pL-mCherry under tumor-targeted NIR. All images in this figure are representative of three experiments.

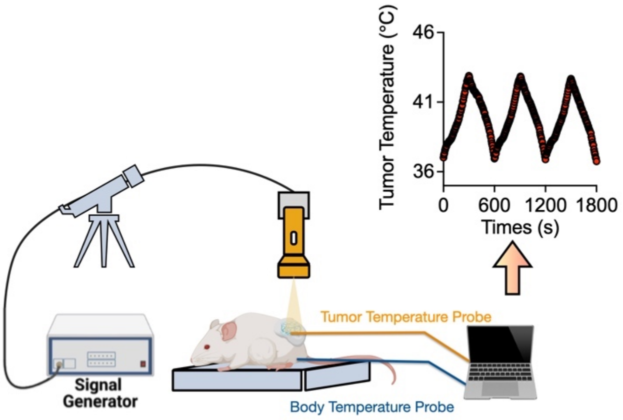

Figure S17.

The use of temperature transducers and near-infrared lasers can accurately measure and raise the temperature within tumor tissue[5]. In this study, a temperature conduction device was used to adjust the power density of NIR to explore the temperature changes of tumors. The NIR probe was 5 cm away from the tumor edge of the mice. The NIR probe was directed to the intra injection site of the azido-HA, and to measure the internal tumor temperature during heating, we temporarily implanted a thin fiber optic temperature probe (Neoptix, T1-02-B05) into the tumor. The custom probe has a sensing tip with a diameter of 400 μm and a length of less than 2 mm. To insert the probe into the tumor, we shaved the hair from the tumor site of the mice in the treatment group, inserted a 25-gauge needle into the tumor to guide the temperature sensing probe, inserted a fiber optic probe into the path created by the needle, and secured the probe with duct tape, running a MATLAB closed-loop thermal control script on the computer to measure the temperature signal. In order to increase tumor temperature precisely, the conditions of NIR were fixed except for different power, that is, 808 nm wavelength, fixed irradiation distance, 5 cm, irradiation time, 5 min ON, 5 min OFF for each cycle, three cycles, a total of 30 min.

Effective thermal control is achieved by modifying the power density of the NIR to achieve the desired temperature in the target tissue.

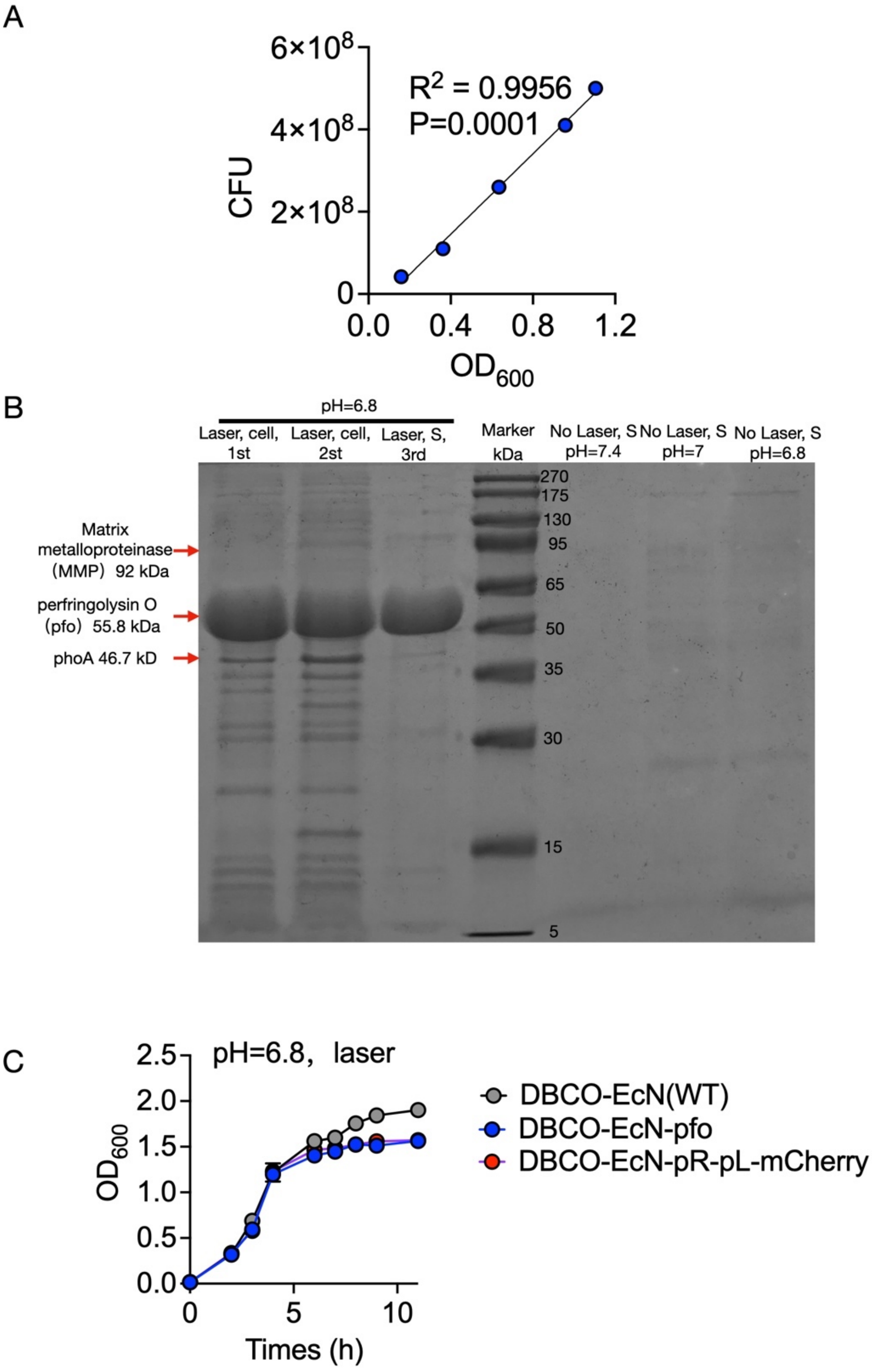

Figure S18.

(**A**) Correlation between OD_{600} and CFUs of EcN. Simply, EcN-pfo performed 12-hour growth curve measurements, while CFU values were measured at 2, 4, 6, 8 hours. (**B**) SDS-PAGE analysis of bacterial extractive whole-cell (cell) proteins and secreted proteins (S)

from DBCO-EcN-pfo with Matrix metalloproteinase (MMP) at different pH levels, with or without NIR treatment: Lane 1, pH = 6.8, NIR irradiation once, whole-cell proteins; Lane 2, pH = 6.8, NIR irradiation twice, whole-cell proteins; Lane 3, pH = 6.8, NIR irradiation thrice, secreted proteins; Lane 4, marker; Lane 5, pH = 7.4, no NIR, secreted proteins; Lane 6, pH = 7, no NIR, secreted proteins; Lane 7, pH = 6.8, no NIR, secreted proteins. (**C**) Minimal difference between the effects of plasmid (pBV220-BRP-*phoA*-pCadC-Lpp-ompA-S_{MMP}-*pfo*-Axe-Txe) loads on DBCO-EcN-pfo and plasmid (pBV220pR-pL-*mCherry*) loads on DBCO-EcN-pR-pL-mCherry bacterial growth under conditions of pH = 6.8 and NIR irradiation, demonstrating that the bioengineered EcN of the STEPT system did not have an excessive growth load. Data represent 3 biologically independent samples.

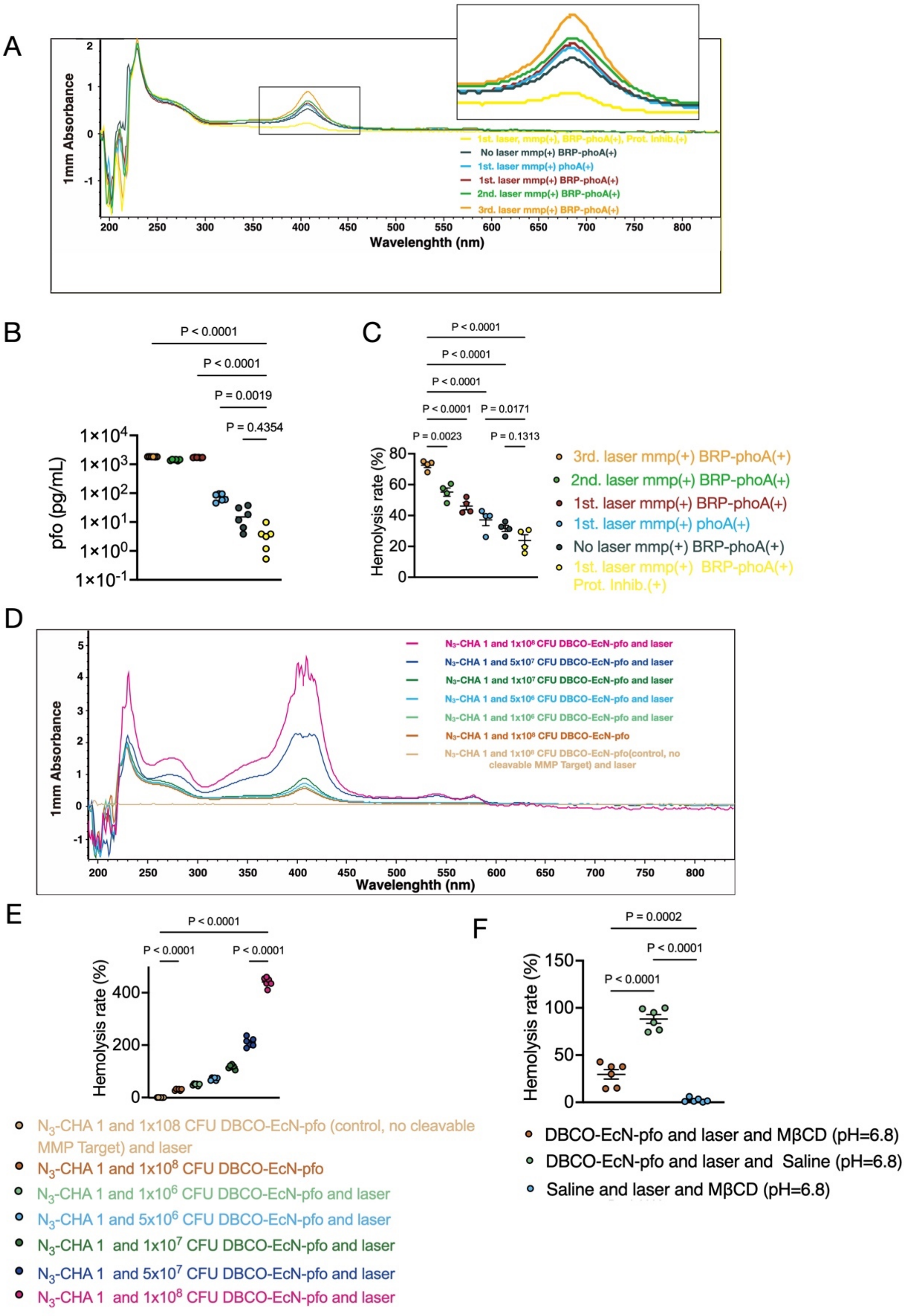

Figure S19.

Hemolysis of EcN-pfo in tumor sphere models with N_3-CHA 1. (**A to C**) Hemolysis in supernatants of tumor sphere cultures under different NIR treatment conditions, with or

without protein inhibition (Prot. Inhib.) by the MMPs inhibitor (astragaloside IV) following bacterial colonization of EcN-pfo with and without BRP; n = 10 biologically independent samples. (**A**) After full wavelength scanning, the level of hemoglobin released by the erythrocyte was estimated as a hemolytic indicator based on absorption measurements at 406 nm. (**B**) pfo quantification in supernatants of tumor sphere cultures after bacterial colonization under different treatment conditions in **A**; n = 6 biologically independent samples. (**C**) Hemolysis rate in supernatants of tumor sphere cultures after bacterial colonization under different treatment conditions in **A**; n = 4 biologically independent samples. (**D**) Hemolytic assay of supernatants of tumor sphere cultures treated with different CFUs of DBCO-EcN-pfo and DBCO-EcN-pfo (control, no cleavable MMPs target, expressing pfo precursors without MMP targeting) after bacterial colonization. (**E**) Hemolysis rate in supernatants of tumor sphere cultures after bacterial colonization with a range of CFUs and different treatment conditions; n = 6 biologically independent samples. (**F**) Decreased hemolysis rate in supernatants of STEPT in tumor sphere cultures under treatment with beta-methylcyclodextrin (MβCD), which eliminates cholesterol from cell membranes; n = 6 biologically independent samples. Data in **B**, **C**, **E**, and **F** expressed as the mean ± SEM. P values determined by one-way ANOVA with Tukey's post-hoc test (**B**, **C**, **E**, **F**).

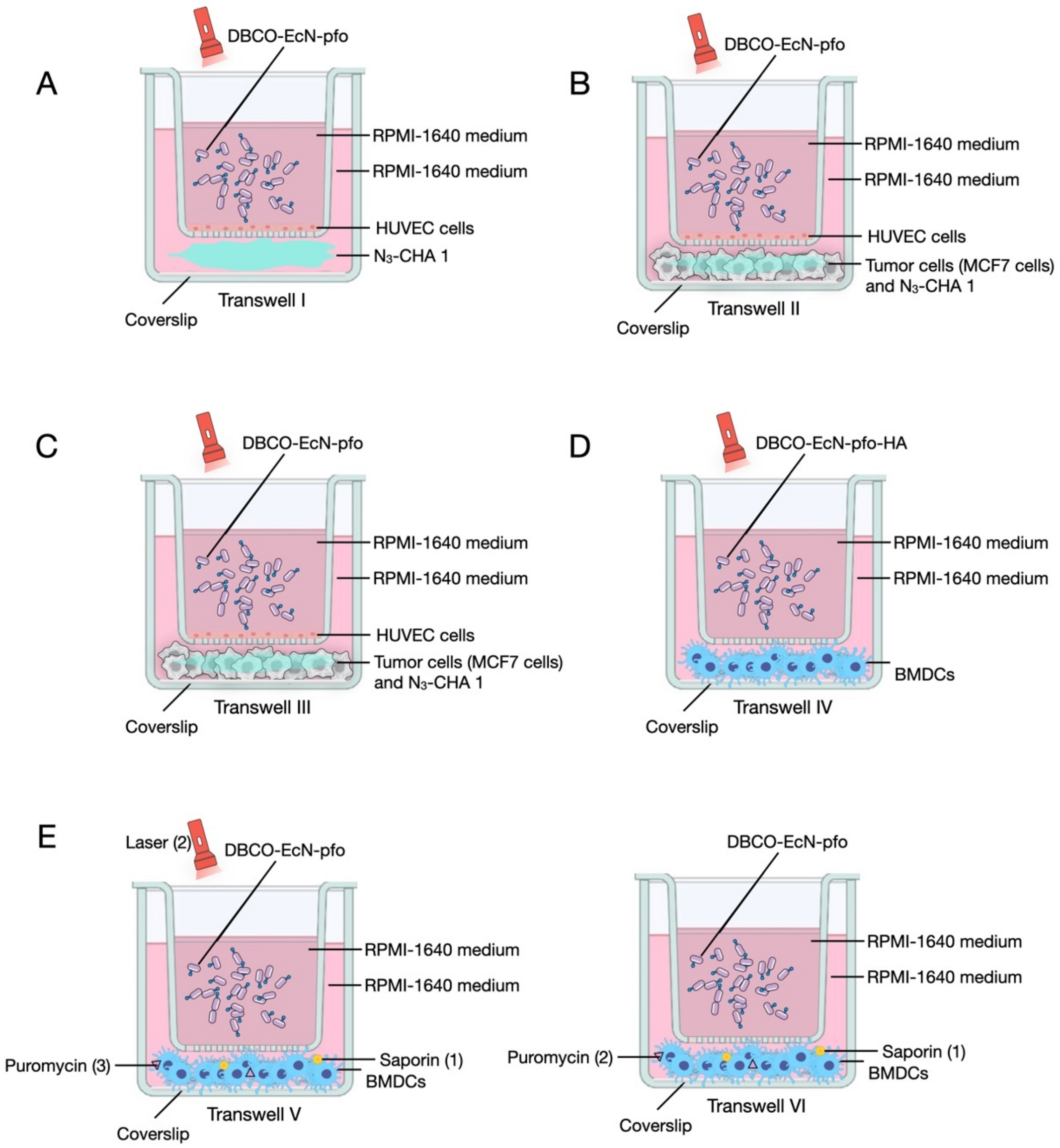

Figure S20.

Schematic illustrations of the transwell systems (I–VI) featured in the following Figures: (**A**) Transwell I, Figure 3E; (**B**) Transwell II, Figure 4A; The control transwell treated with no laser. (**C**) Transwell III, Figure 4C. (**D**) Transwell IV, Figure 6B. Pfo and lysosome are co-localized in BMDCs by using a transwell system. A polylysine-treated coverslip was placed in the lower chamber, which was cultured with BMDCs. 200-μL samples of logarithmic growth cultures of EcN-pfo-HA dispersed in DPBS at a density of 1×10^5 CFU were each added to the top chamber under the following conditions: 0.308 W/cm^2 and 5 cm for 5 min ON; 5 min OFF, and the assembled device was incubated at 37 °C for 30 min. Three rounds of periodic NIR were applied Then EcN-pfo-HA was labeled with anti-

HA–tetramethylrhodamine (TRITC) monoclonal antibody, pHrodo BioParticles and Hoechst 33342. And SIM imaging was performed. (**E**) Transwell V and Transwell VI, Figure 6C. Saporin assay was performed as previously reported[9]. To monitor saporin-mediated activation, BMDCs (3×10^5/well) were seeded in 96-well treated tissue culture U-bottom plates. Cells were pulsed for 30 min at 37 °C with saporin, washed once in PBS, spun at 380 ×g for 3 min, resuspended in transwell VI lower chamber with DC media containing 0.01 mg/mL puromycin. EcN-pfo (NIR treatment) was added to the upper chamber and incubated for 30 min at 37 °C. The control transwell VI treated with no laser. Incorporation was determined by staining with an αPuromycin-AF488 antibody in Wash buffer for 45 min on ice and analyzing the cells by flow cytometry.

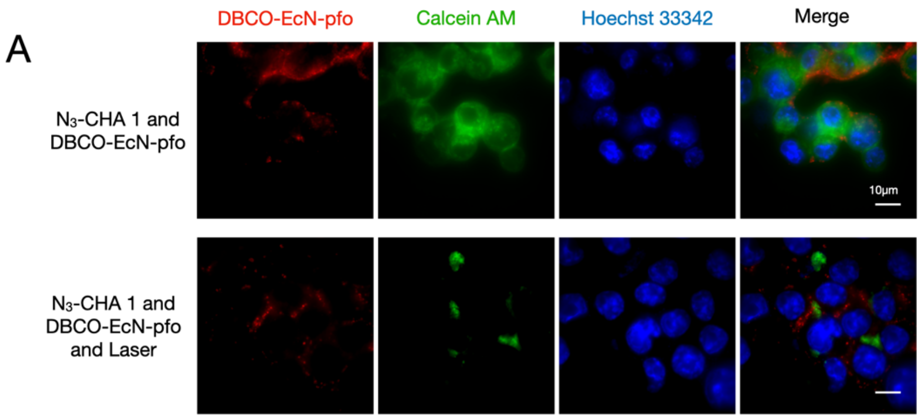

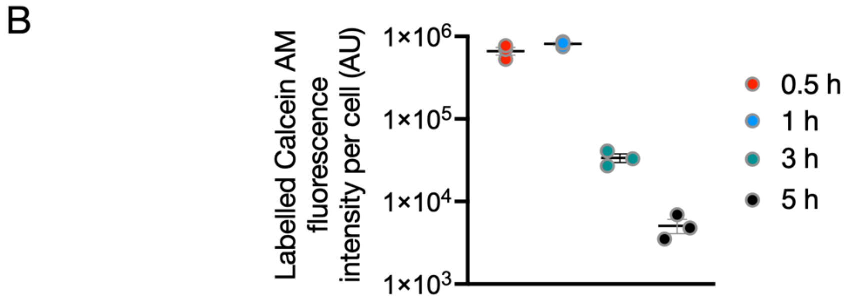

Figure S21.

(A) Tumor killing effect of pfo demonstrated at the cellular level using the transwell device (see Figure S20B). Anti-HA-tetramethylrhodamine (TRITC) antibody labelled EcN-pfo (with HA tag). Calcein AM labeling of MCF7 tumor cells showed weak fluorescence intensity after NIR (laser), and high fluorescence intensity in the absence of NIR. Scale bar, 10 μm. n = 30 biologically independent fields of view. (B) Tumor killing effect of pfo demonstrated at the cellular level in 5 hours. Anti-HA tag-tetramethylrhodamine (TRITC) antibody-labeled EcN-pfo (with HA tag). Calcein AM labeling of MCF7 tumor cells showed weak fluorescence intensity after laser (NIR); AU, arbitrary units. n = 3 biologically independent fields of view.

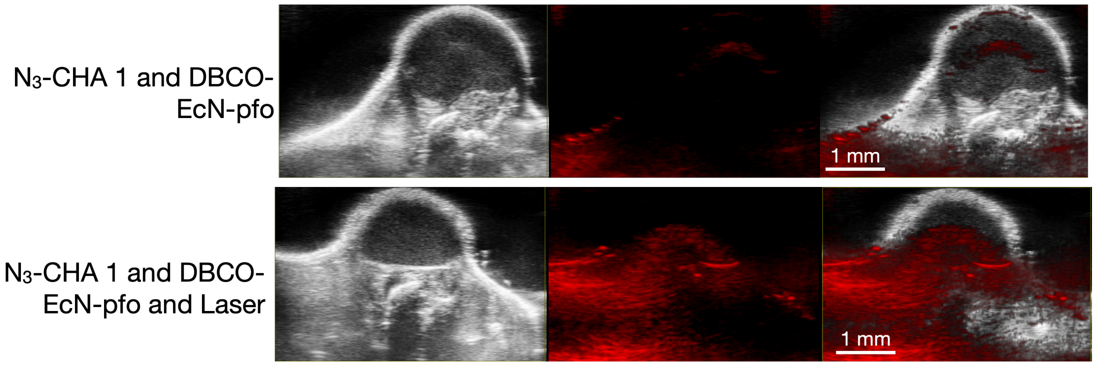

Figure S22.

PA imaging of the tumor from Figure 4D, performed on the 5th day post NIR irradiation. The control group did not receive NIR irradiation; Scale bar, 1 mm. n = 3 biologically independent mice.

A

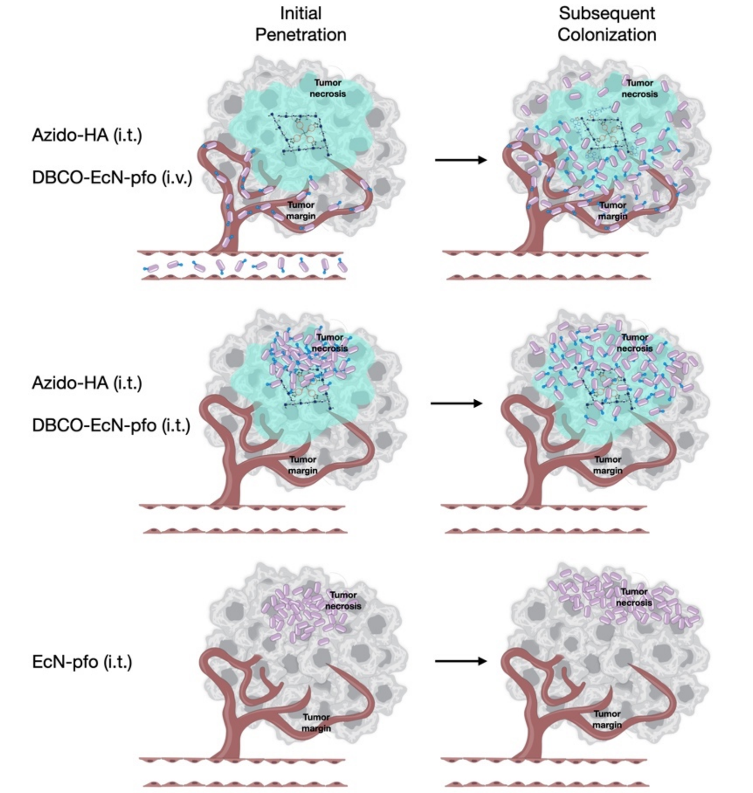

B

G8	G9	G10
N_3-CHA 1 (i.t.) and DBCO-EcN-*pfo* (I.t.) and NIR	PBS (i.t.) and DBCO-EcN-*pfo* (I.t.) and NIR	DBCO-EcN-*pfo* (I.t.) and NIR

C

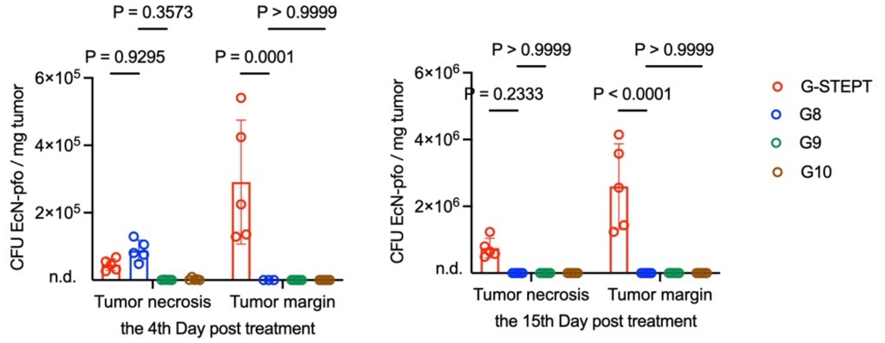

Figure S23.

In order to compare intravenously (i.v.) and intratumorally (i.t.) injected bacteria for intratumor distribution of bacteria, we designed G8, G9 and G10. (**A** and **B**) Distribution diagram of engineered bacteria in the inner margin and necrotic area of the tumor with intratumoral injection of N_3-CHA 1, intratumoral injection of DBCO-EcN-pfo, and NIR

irradiation of tumor (G8). Distribution diagram of engineered bacteria in the inner margin and necrotic area of the tumor with intratumor injection of PBS buffer, intratumoral injection of DBCO-EcN-pfo, and NIR irradiation of tumor (G9).

Distribution diagram of engineered bacteria in the inner margin and necrotic area of the tumor with intratumoral injection of DBCO-EcN-pfo, and NIR irradiation of tumor (G10). (**B**) Grouping of G8, G9 and G10 (**C**) The number of engineered bacteria in G-STEPT, G8, G9 and G10 tumors at tumor margins and necrotic areas was counted on the 4th and 15th days, respectively. n = 5 biologically independent mice. n.d., not detected. Data in **C** expressed as the mean ± SEM. P values determined by one-way ANOVA with Tukey's post-hoc test (**C**). With i.t. injection, the bacteria were mainly distributed in the necrotic area of the distal tumor blood vessels. In contrast, i.v. injected bacteria were distributed in both proximal and distal tumor blood vessels. Bacteria mainly accumulated near blood vessels in tumors, with bacteria-secreted pfo quickly killing tumors and inducing the destruction of tumor blood vessels, leading to tumor thrombosis.

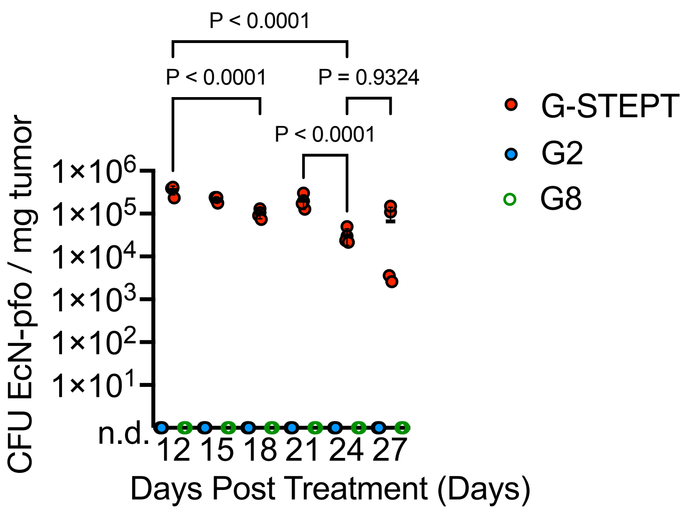

Figure S24.

During the treatment period, the number of bacterial CFU in the tumor was counted. G2 was injected with hyaluronic acid, injected with engineering bacteria intravenously, and irradiated with NIR. G8 was used for intratumoral injection of N_3-CHA 1, intratumoral injection of engineered bacteria, and NIR irradiation of tumors. n = 4 biologically independent mice. n.d., not detected. P values determined by one-way ANOVA with Tukey's post-hoc test.

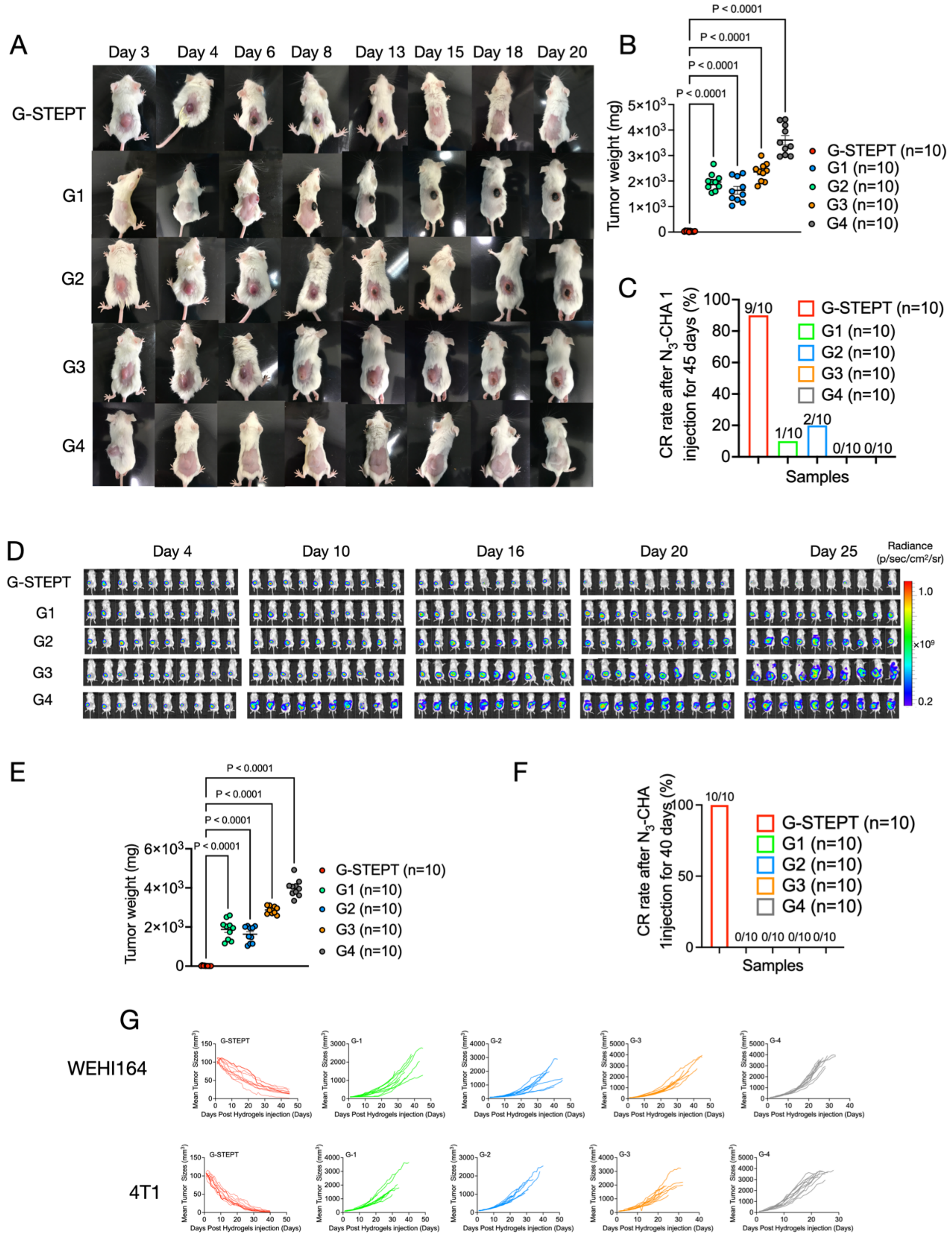

Figure S25.

(**A to C**) Effect of STEPT on S.C. fibrosarcoma in mice. See more for timeline of treatments in Figure 5A and B. (**A**) Time course of the effects of the indicated STEPT treatments on solid tumors as shown by photographs of fibrosarcoma model mice. (**B**)

Tumor weight measurements, and (**C**) CR (complete response) rates of tumor-bearing mice after the indicated treatments, the G-STEPT also achieved a complete response in 100 % of tumors. n= 10 biologically independent mice. (**D** to **F**) Effect of STEPT on orthogonal breast cancer with high rates of metastasis in mice. See Figure 5E for more details. Representative images and quantification of bioluminescence intensity of 4T1-luc tumor-bearing mice with primary and distal metastatic tumors. IVIS images of orthotopic breast cancer model mice (**D**). Tumor weight measurements (**E**), and CR rates (**F**) of tumor-bearing mice after the indicated treatments; n= 10 biologically independent mice. (**G**)Curves of tumor volume changes in different treatment groups of mice: Fibrosarcoma (top), Triple-negative breast cancer (bottom). Data in **B** and **E** expressed as the mean ± SEM. P values determined by one-way ANOVA with Tukey's post-hoc test (**B** and **E**).

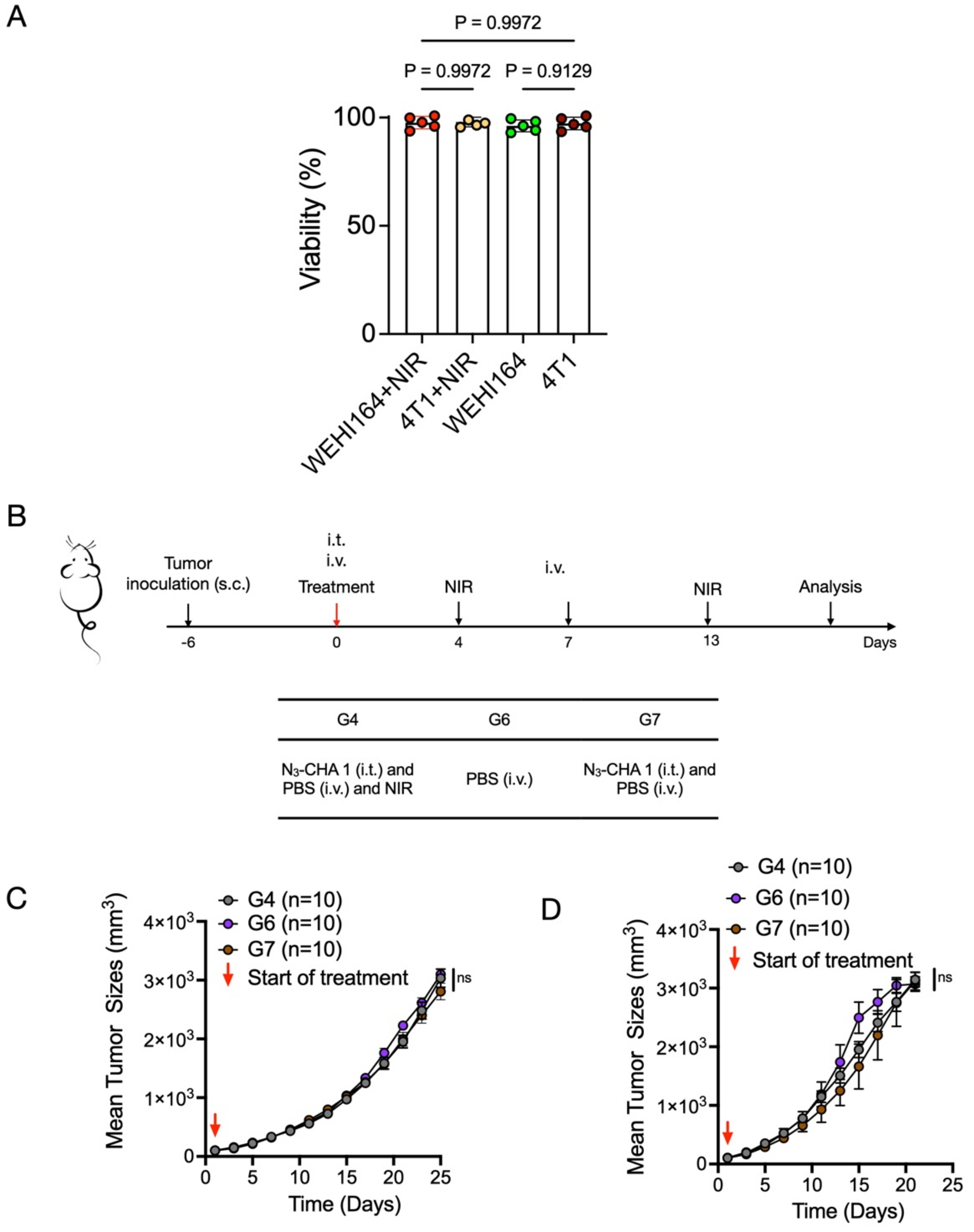

Figure S26.

(**A**) Tumor cytotoxicity *in vitro*. Study on the effect of NIR on survival rate of tumor cells. n= 5 biologically independent samples. (**B**) Experimental group of tumor treatment *in vivo*: G4 was intratumoral injection of N_3-CHA 1, intravenous injection of PBS, and NIR irradiation of tumor; G6 was intravenously injected with PBS; G7 was intratumoral injection of N_3-CHA 1, and intravenous injection of PBS. n= 10 biologically independent

mice. (**C**) In fibrosarcoma mouse tumor model, tumor volume changes in G4, G6 and G7 groups after treatment. n= 10 biologically independent mice. (**D**) In orthotopic mouse tumor models of breast cancer, tumor volume changes in G4, G6 and G7 groups after treatment. n= 10 biologically independent mice. Data in **A**, **C** and **D** expressed as the mean ± SEM. P values determined by two-way ANOVA with Bonferroni post-hoc test (**C** and **D**) and one-way ANOVA with Tukey's post-hoc test (**A**).

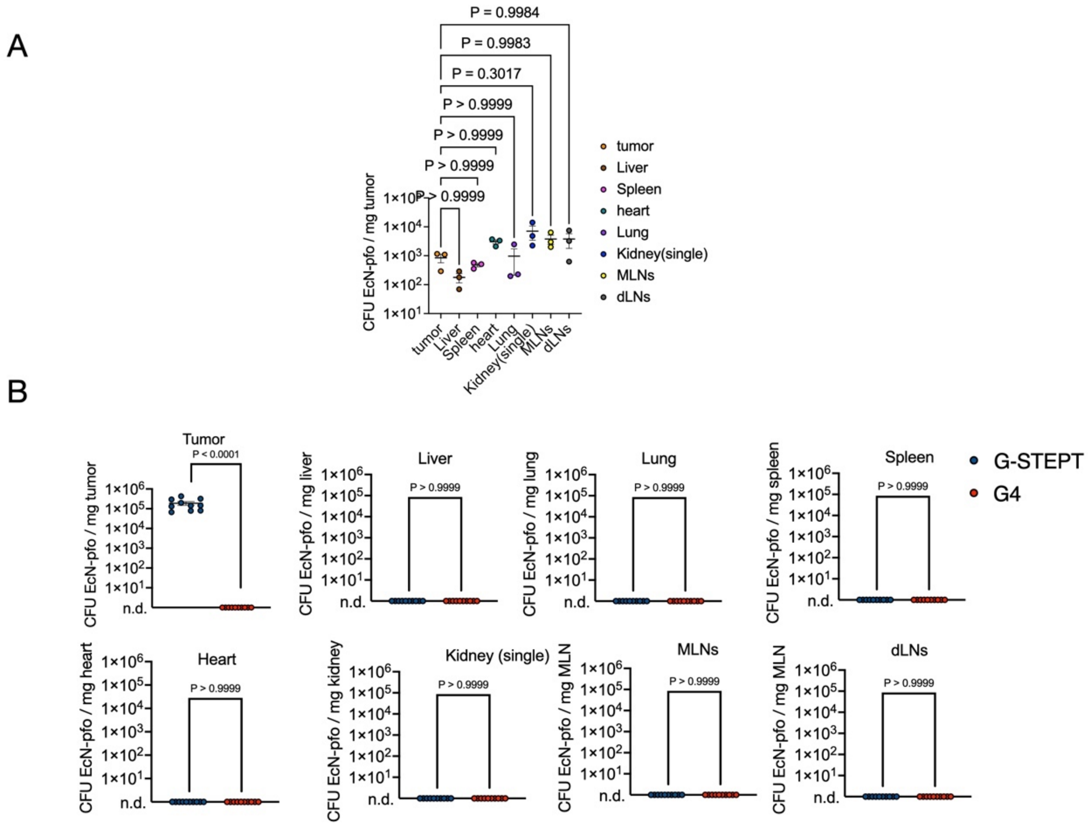

Figure S27.

Safety assessment of STEPT therapy. (**A**) CFUs of EcN cultured in tumors and the indicated organs harvested from the healthy mice to test whether the observed differences in bacterial growth were dependent on in-situ environmental characteristics; n = 3 biologically independent mice. All bacteria grew normally and at the same rate. (**B**) On the 20th day after the indicated STEPT treatment, mouse tumors, lymph nodes and organs were dissected and weighed, homogenized under sterile conditions, and CFUs of EcN-pfo in each group were calculated; n = 10 biologically independent mice; n.d., not detected. Data in **A** and **B** expressed as the mean ± SEM. P values determined by unpaired two-tailed Student's t-test (**B**), one-way ANOVA with Tukey's post-hoc test (**A**).

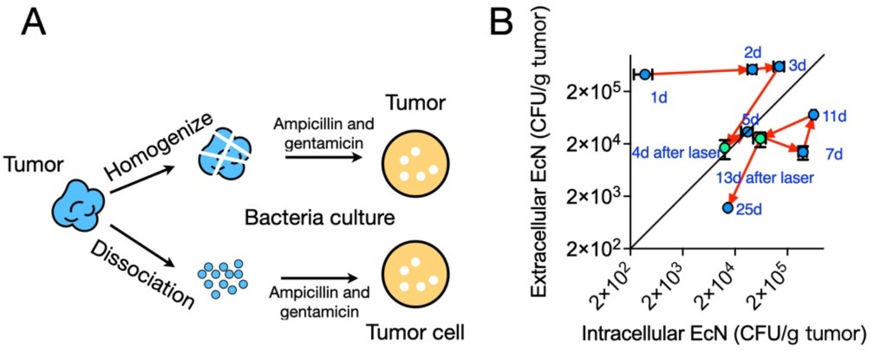

Figure S28.

(**A** and **B**) Statistical analysis of intracellular and extracellular EcN CFUs in tumors during STEPT treatment[10]; n = 5 biologically independent mice.

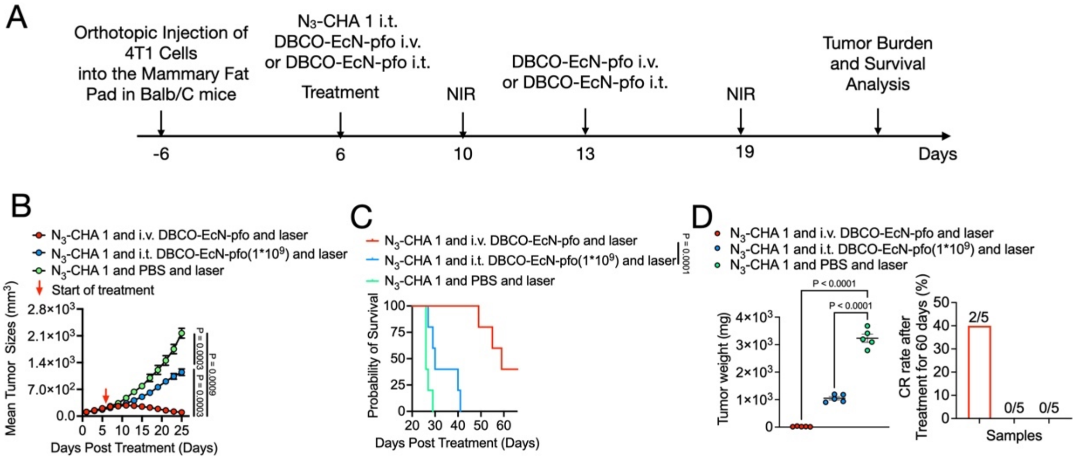

Figure S29.

Treatment of larger tumors with STEPT. Treatment of larger tumors with immunotherapies is reportedly challenging, in part because of the establishment of a more immunosuppressive environment that further inhibits T-cell penetration and function. Therefore, based on the observed strong therapeutic response to our STEPT system, we tested it in mice with larger (approximately 200–260 mm^3) orthotopic 4T1-luc breast tumors. Compared with a high dose of DBCO-EcN-pfo intratumor injection, G-STEPT treatment inhibited the growth of large tumors and extended survival times to a certain extent. These studies supported the potential of STEPT as a technology to enhance the response to cancer vaccines, adoptive T-cell transfer, and chimeric antigen receptor (CAR) T-cell therapy. (**A**) Illustration of experimental protocols for STEPT therapy in 4T1-luc model mice with large initial tumors. (**B** to **D**) Tumor growth curves (**B**), survival curves (**C**), and tumor weight measurements and CR rates (**D**) after the indicated treatment in large 4T1-luc tumor-bearing mice; n = 5 biologically independent mice. Data in **B**, **C**, and **D** expressed as the mean ± SEM. P values determined by unpaired two-tailed Student's t-test (**D**), two-way ANOVA with Bonferroni post-hoc test (**B**), or log-rank (Mantel–Cox) test for survival curves (**C**).

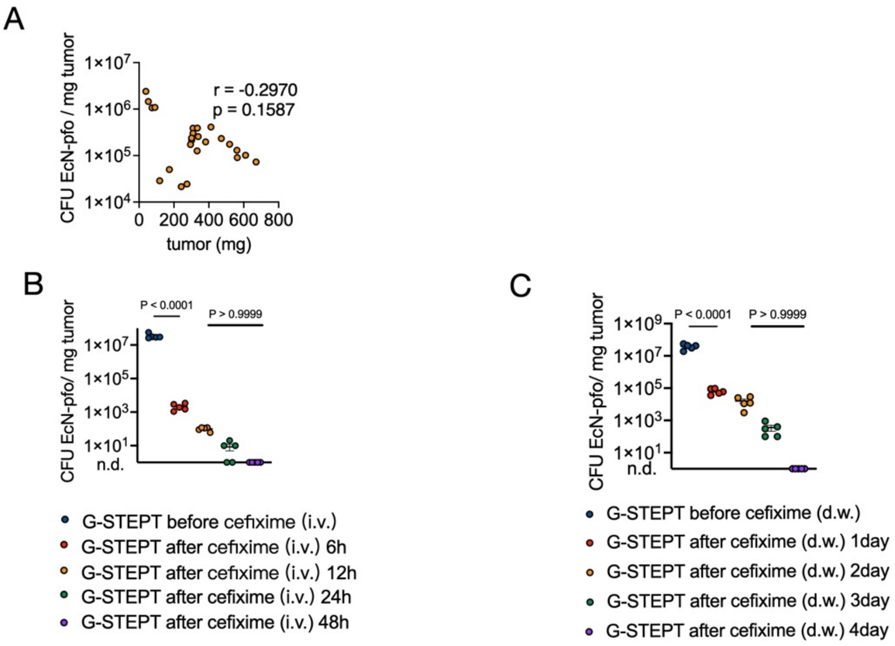

Figure S30.

(**A**) Correlation between tumor weight and EcN-pfo CFUs within tumors. (**B**) Changes in CFUs of EcN-pfo under intravenous administration of 100 mg/kg cefixime, showing complete EcN-pfo clearance within 48 h; n = 5 biologically independent mice. (**C**) Changes in CFUs of EcN-pfo under oral administration of cefixime (10 mg/kg in water), showing complete EcN-pfo clearance within 4 days; n = 5 biologically independent mice. Data in **A** to **C** expressed as the mean ± SEM. P values determined by one-way ANOVA with Tukey's post-hoc test (**B** and **C**).

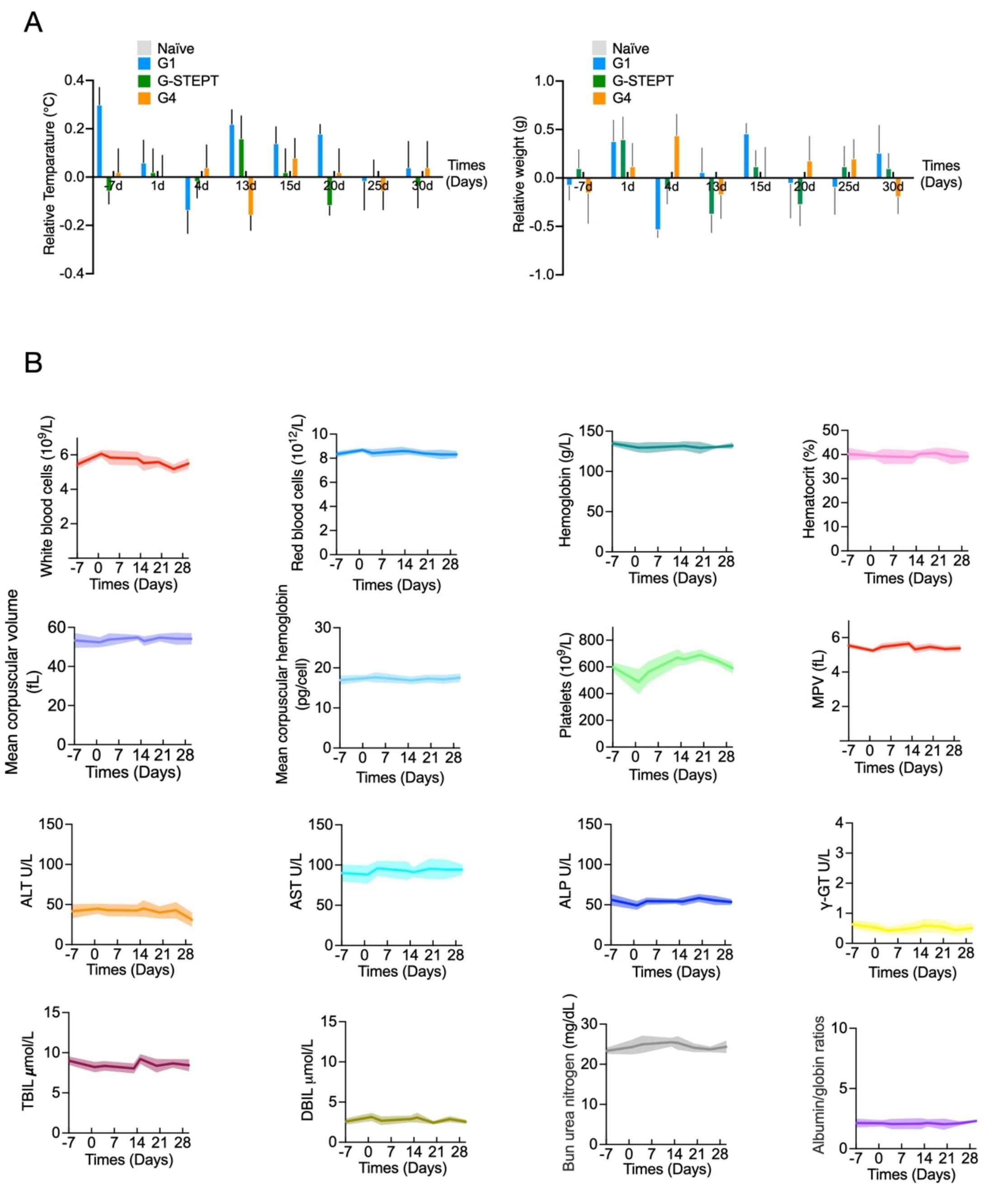

Figure S31.

(**A**) Relative changes in temperature and weight in STEPT-treated 4T1 breast tumor model mice from the beginning of tumor implantation (day -7) to the end of treatment (day 30); n = 5 biologically independent mice. (**B**) Biochemical, liver function, and renal function indicators in STEPT-treated 4T1 breast tumor model mice from the beginning of tumor implantation (day -7) to the end of treatment (day 30). DBIL/TBIL, direct/total bilirubin;

ALT, alanine aminotransferase; ALP, alkaline phosphatase; AST, asparate aminotransferase; Bun, blood urea nitrogen; γ-GT, glutamyl transpeptidase; MPV, mean platelet volume.

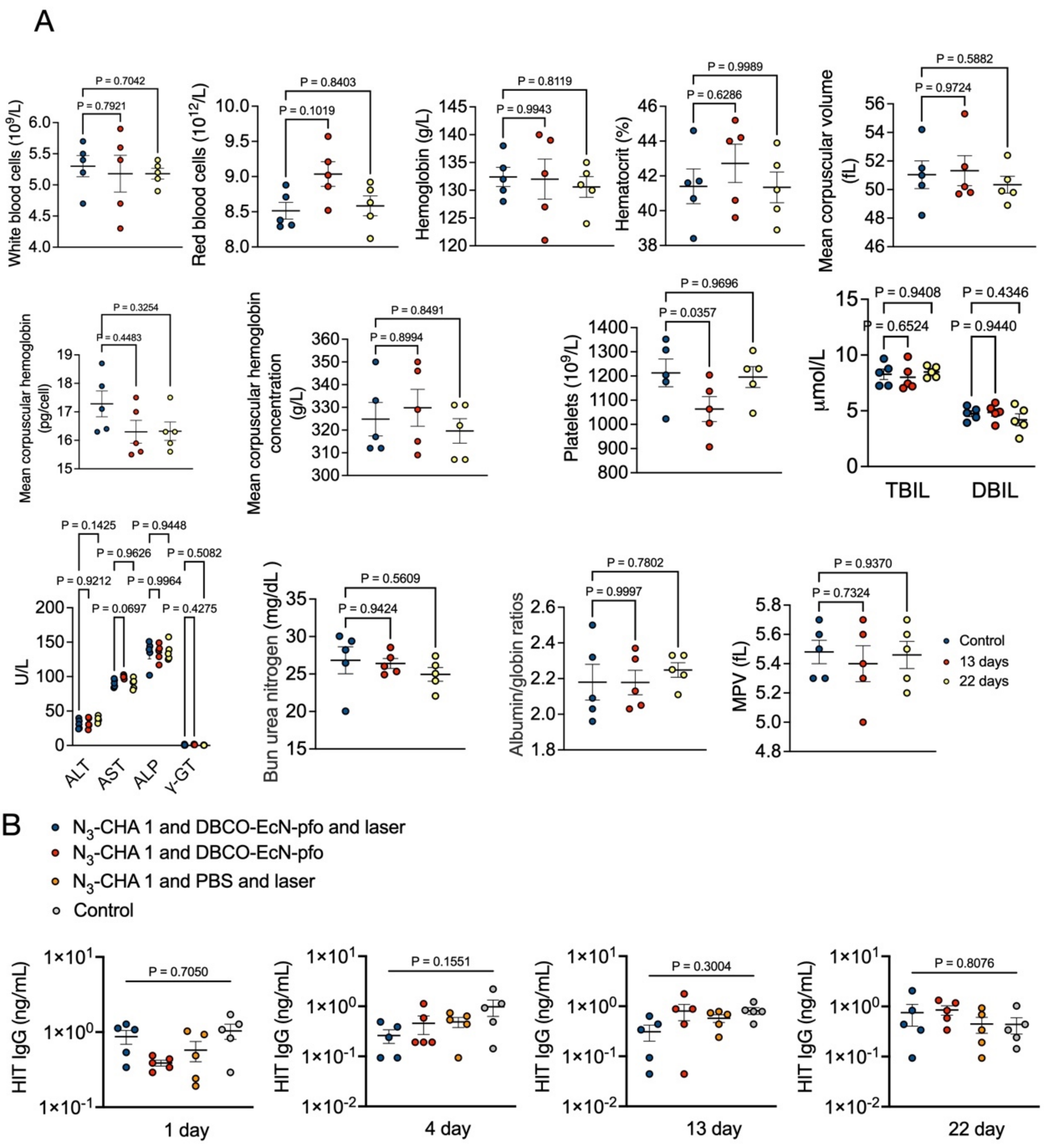

Figure S32.

(**A**) Biochemical, liver function, and renal function indicators examined at days 13 and 22 in 4T1 breast tumor model mice treated with STEPT; n = 5 biologically independent mice. (**B**) To rule out heparin-induced thrombocytopenia as the cause of these changes, we surveyed the levels of serum heparin-platelet factor 4 complex immunoglobulin G antibodies (HIT IgG) at days 1, 4, 13, and 22 in treated 4T1 model mice, and found that they were not significantly different from healthy controls; n = 5 biologically independent

mice. Data in **A** and **B** expressed as the mean ± SEM. P values determined by one-way ANOVA with Tukey's post-hoc test.

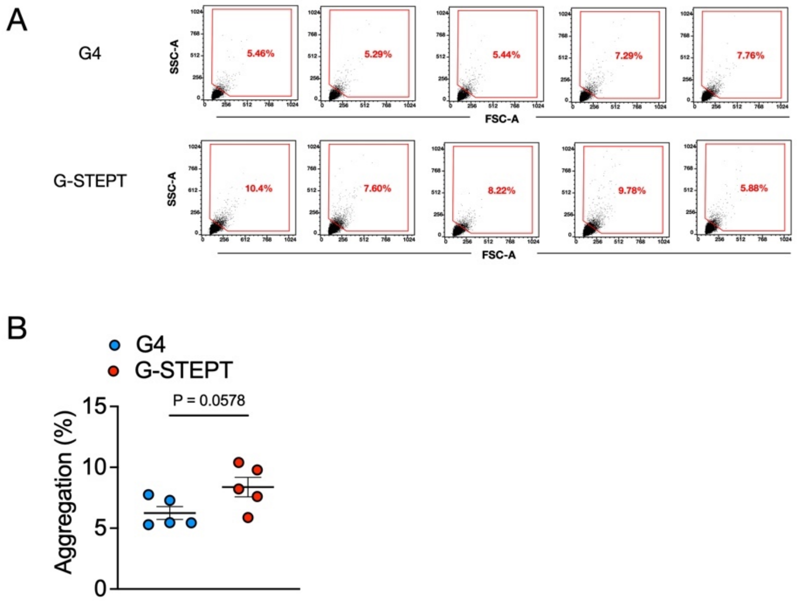

Figure S33.

(**A**) Flow cytometry analysis of platelet activation in the blood of mice receiving the indicated STEPT treatment or control (G4); n = 5 biologically independent mice. (**B**) Calculated results of blood coagulation testing; n = 5 biologically independent mice. P values determined by unpaired two-tailed Student's t-test.

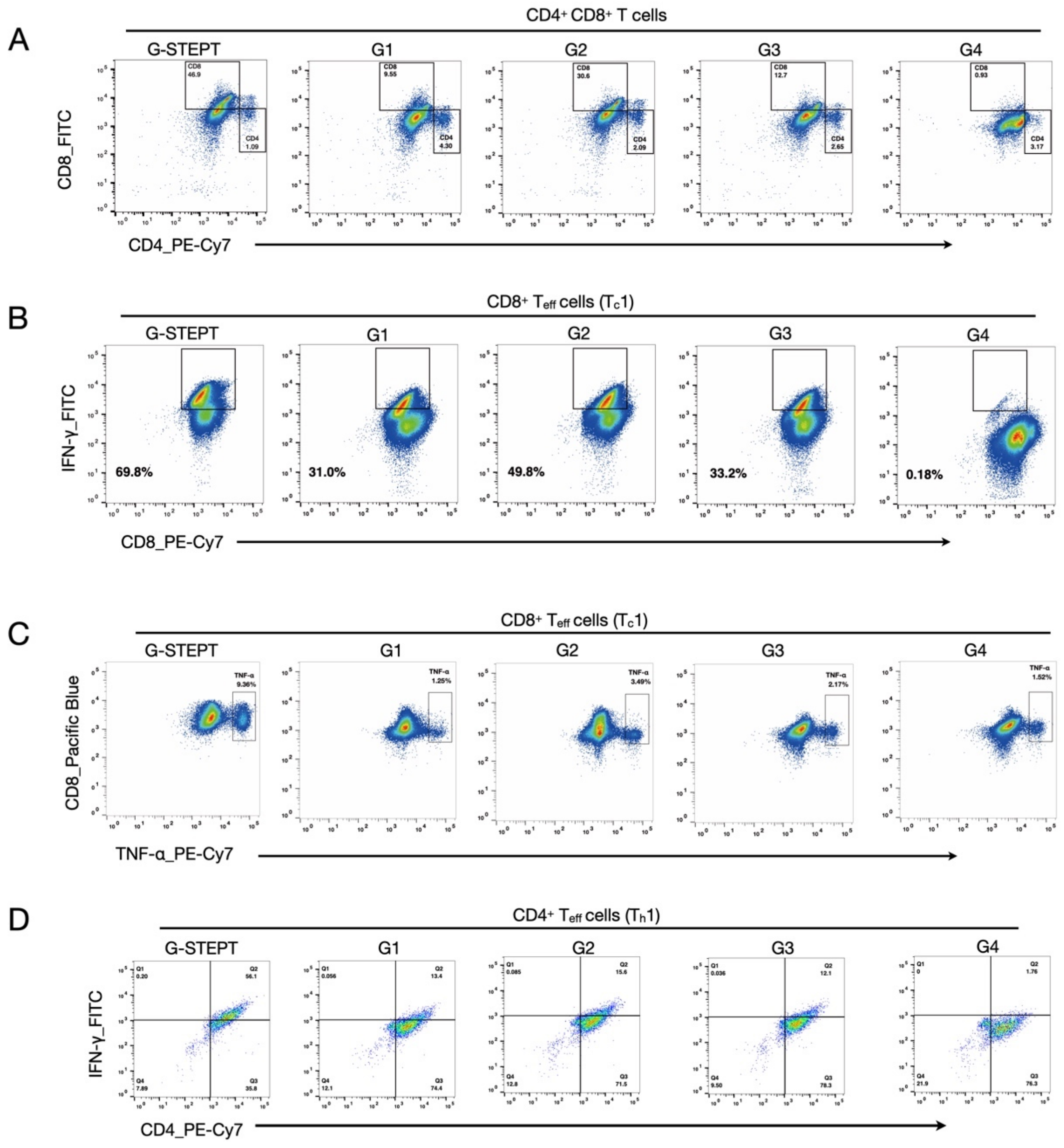

Figure S34.

Representative flow cytometry histograms illustrating changes in the following tumor-infiltrating cells in tumors at 14 days after the indicated treatments (see Figure 5B): (**A**) comparison of endogenous CD4$^+$ and CD8$^+$ T cells (gated on CD45$^+$ CD3$^+$ cells); (**B**) changes in intracellular IFN-γ$^+$ CD8$^+$ T cells (gated on CD45$^+$ CD3$^+$ CD8$^+$ cells); (**C**) changes in intracellular TNF-α$^+$ CD8$^+$ T cells (gated on CD45$^+$ CD3$^+$ CD8$^+$ cells); (**D**) changes in intracellular IFN-γ$^+$ CD4$^+$ T cells (gated on CD45$^+$ CD3$^+$ CD4$^+$ cells).

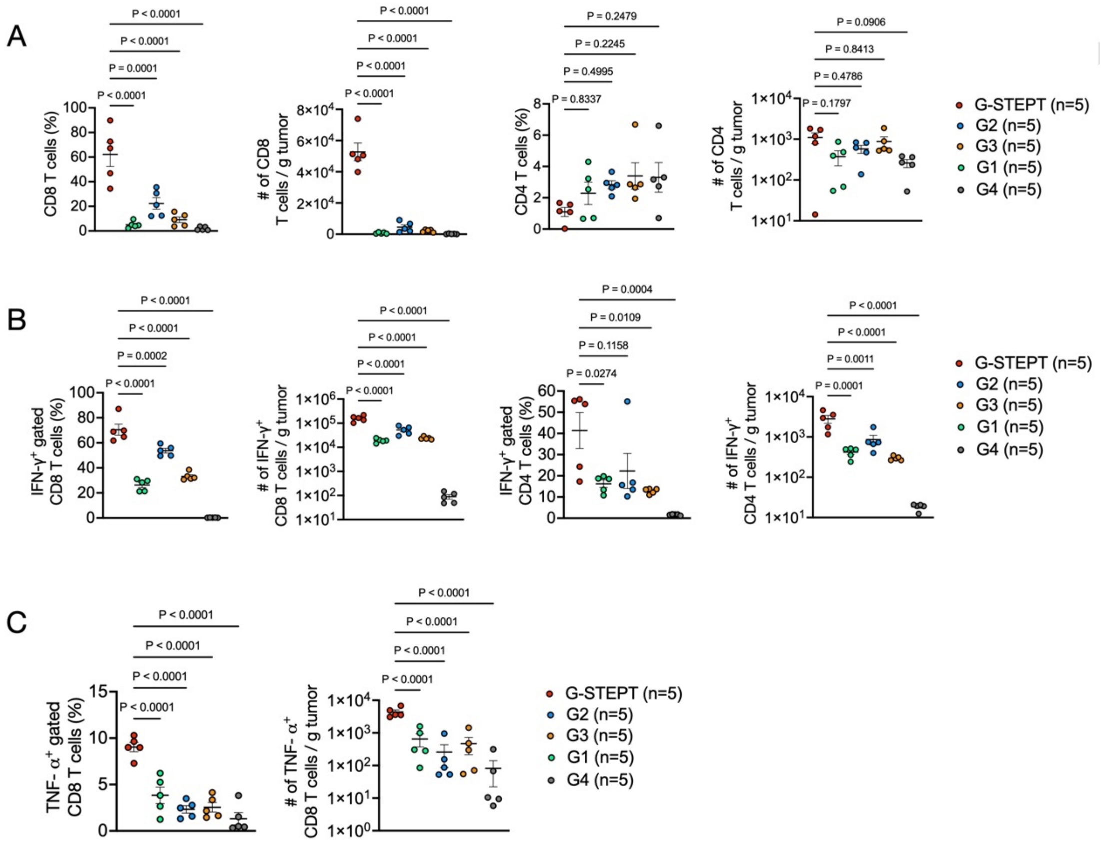

Figure S35.

Activation effect of STEPT therapy on CD8$^+$ T cells, resulting in mediation of antitumor immunity. (**A** to **C**) Activation effect of STEPT therapy on CD8$^+$ T cells. Tumor-bearing model mice established by orthotopic injection of 4T1-luc cells into the mammary fat pad in BALB/c mice. Representative flow cytometry quantitative analysis illustrating changes in the following tumor-infiltrating cells in tumors at 14 days after the indicated treatment: (**A**) comparison of endogenous CD4$^+$ and CD8$^+$ T cells (gated on CD45$^+$ CD3$^+$ cells); (**B**) changes in intracellular IFN-γ$^+$ CD8$^+$ T cells (gated on CD45$^+$ CD3$^+$ CD8$^+$ cells) and IFN-γ$^+$ CD4$^+$ T cells (gated on CD45$^+$ CD3$^+$ CD4$^+$ cells); (**C**) changes in intracellular TNF-α$^+$ CD8$^+$ T cells (gated on CD45$^+$ CD3$^+$ CD8$^+$ cells); n = 5 biologically

independent samples. Data in **A** to **C** expressed as the mean ± SEM. P values determined by: one-way ANOVA with Tukey's post-hoc test (**A** to **C**).

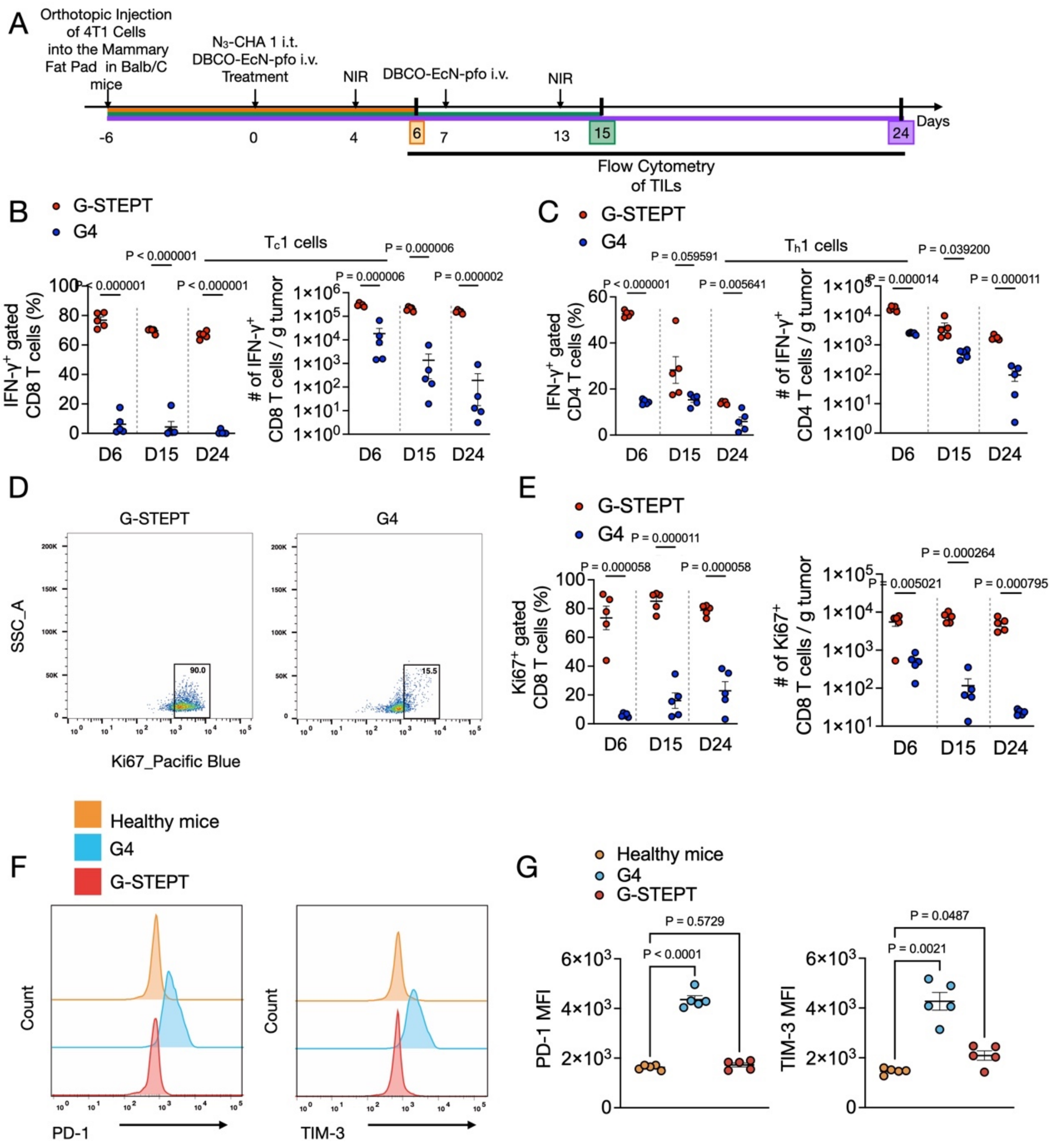

Figure S36.

(**A**) Experimental protocol for STEPT treatment and flow cytometry analysis of tumor-infiltrating lymphocytes (TILs) in orthotopic breast cancer model mice on day 6 (yellow line), day 15 (green line), and day 24 (purple line); n = 5 mice per group. (**B** and **C**) Percentage (left panel) and total number of cells normalized to grams of tumor tissue (right panel) of IFN-γ⁺ Tc1 cells (**B**) and IFN-γ⁺ Th1 cells (**C**). (**D**) Representative flow cytometry histograms of Ki67⁺ Tc1 cells. (**E**) The percentage (left panel) and total number of cells

normalized to grams of tumor tissue (right panel) of Ki67^{+} Tc1 cells. (**F** and **G**) Representative flow cytometry histograms illustrating changes in the following tumor-infiltrating cells in tumors at 14 days after the indicated treatments (see Figure 5E): comparison of endogenous PD-1^{+}CD8^{+} T cells (gated on CD45^{+} CD3^{+} CD8^{+} cells) and TIM-3^{+} CD8^{+} T cells (gated on CD45^{+} CD3^{+} CD8^{+} cells); n = 5 mice per group. Data in **B**, **C**, **E** and **G** expressed as the mean ± SEM. P values determined by unpaired two-tailed Student's t test (**B, C** and **E**), one-way ANOVA with Tukey's post-hoc test (**G**).

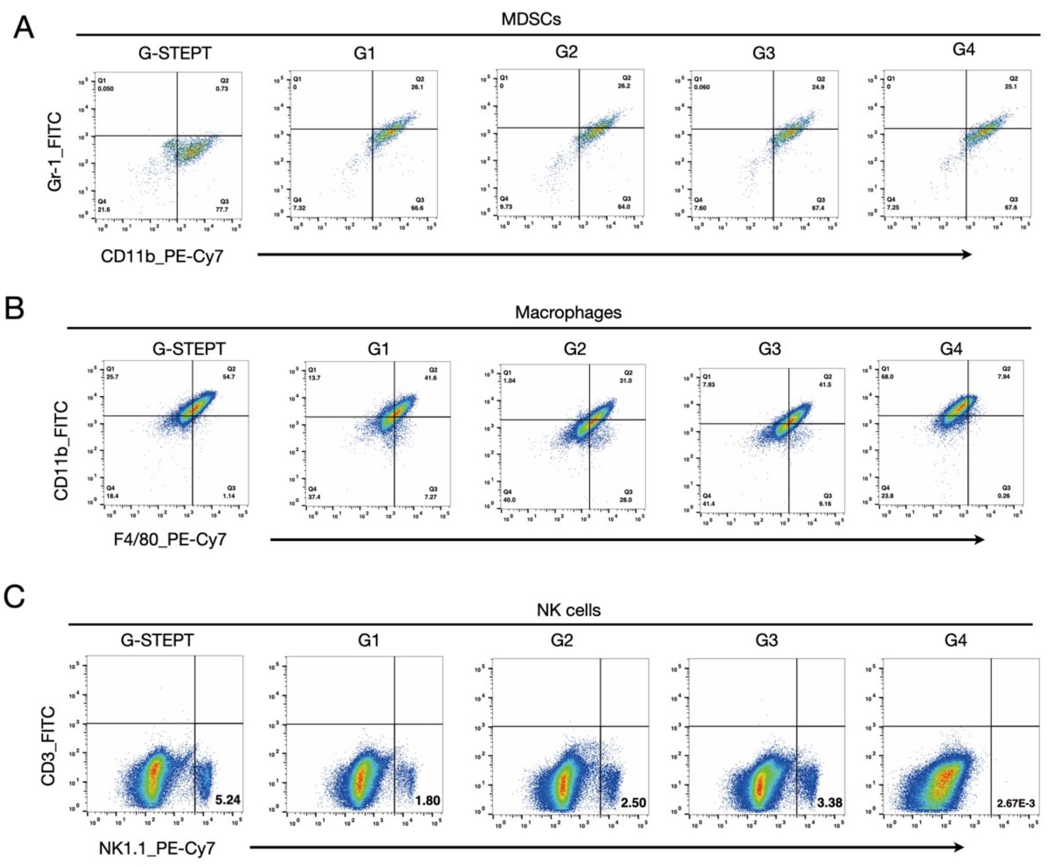

Figure S37.

Tumor-bearing model mice established by orthotopic injection of 4T1 cells into the mammary fat pad in Balb/C mice. (**A** to **C**) Representative flow cytometry histograms and quantitative analysis illustrating changes in the following tumor-infiltrating cells in tumors at 14 days after the indicated treatment: MDSCs (**A**), macrophages (**B**), and NK cells (**C**).

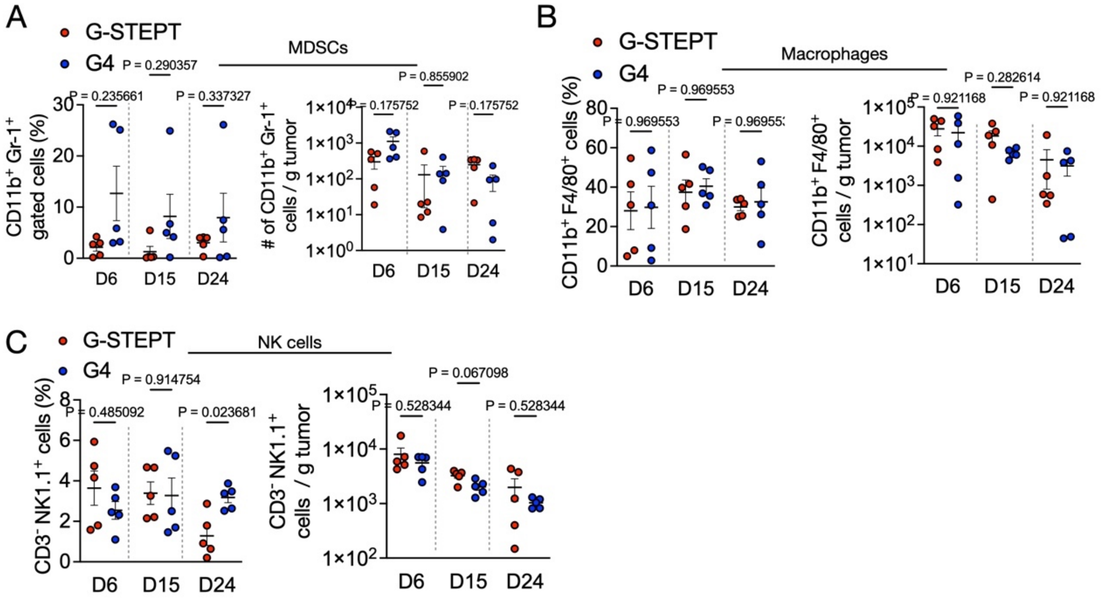

Figure S38.

(**A** to **C**) The percentage (left panel) and number of cells normalized to grams of tumor tissue (right panel) of myeloid-derived suppressor cells (MDSCs, **A**), macrophages (**B**), or natural killer cells (NK cells, **C**); n = 5 mice per group. Data in **A, B** and **C** expressed as the mean ± SEM. P values determined by unpaired two-tailed Student's t test (**A, B** and **C**).

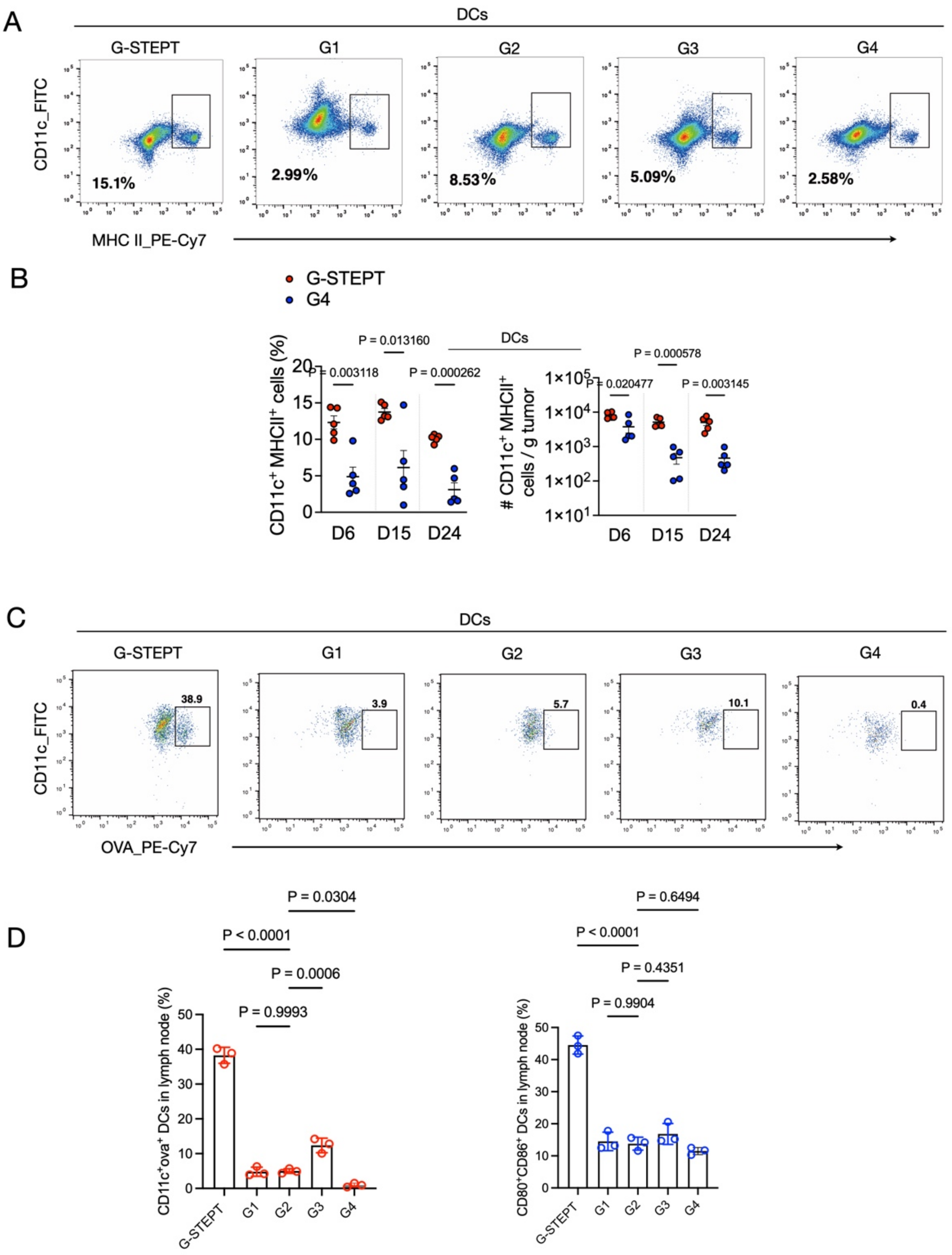

Figure S39.

Representative flow cytometry histograms illustrating changes in the following tumor-infiltrating cells in tumors at 14 days after the indicated treatments (see Figure 5B): (**A**) changes in intracellular CD11c$^+$ MHC II$^+$ DCs (gated on CD45$^+$ CD3$^-$ cells). (**B**) Quantitative analysis of endogenous CD11c$^+$ MHC II$^+$ DCs (gated on CD45$^+$ CD3$^-$ cells) in tumors at days 6, 15, and 24 after the indicated treatment Activation of CD8$^+$ T cells by DC-mediated,

STEPT-induced antitumor immunity. n = 5 biologically independent samples. (**C** and **D**) B16-OVA melanin tumor models were established in C57BL/6J mice and treated in groups according to Figure 5B. After the end of treatment, on day 15, the inguinal lymph nodes surrounding the tumor were extracted to assess the percentage of $OVA^{+}CD11c^{+}$ cells. The results showed that STEPT system effectively increased the percentage of OVA^{+}DCs cells compared to other treatments, demonstrating that the antigen was efficiently delivered to the lymph nodes. STEPT system also significantly activated more DCs maturation ($CD11c^{+}CD80^{+}CD86^{+}$) in the inguinal lymph nodes, conducive to initiating anti-tumor T cell responses. n = 3 biologically independent samples. Data in **B** and **D** expressed as the mean ± SEM. P values determined by unpaired two-tailed Student's t-test (**B** and **D**).

A

G4	G6	G7
N_3-CHA 1 (i.t.) and PBS (i.v.) and NIR	PBS (i.v.)	N_3-CHA 1 (i.t.) and PBS (i.v.)

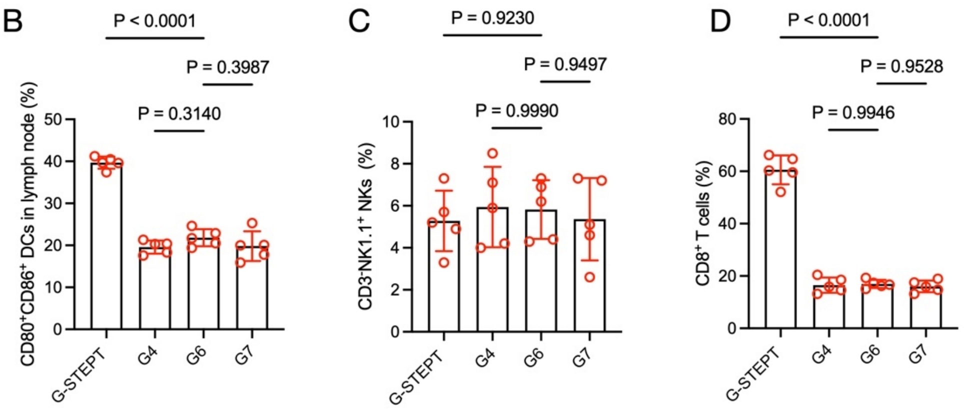

Figure S40.

(A) Experimental group of tumor treatment *in vivo*: G4 was intratumoral injection of azido-HA, intravenous injection of PBS, and NIR of tumor; G6 was intravenously injected with PBS; G7 was intratumoral injection of azido-HA and intravenous injection of PBS. n= 10 biologically independent mice. (**B** to **D**) Study on the effect of NIR on immune cells in this experiment. (**B**) Quantitative analysis of endogenous $CD11c^+$ MHC II^+ $CD80^+$ $CD86^+$ DCs (gated on $CD45^+$ $CD3^-$ cells) in lymph node at day 6. n= 5 biologically independent samples. (**C**) Quantitative analysis of endogenous $NK1.1^+$ NK cells (gated on $CD45^+$ $CD3^-$ cells) in tumor at day 6. n= 5 biologically independent samples. (**D**) Quantitative analysis of endogenous $CD8^+$ T cells (gated on $CD45^+$ $CD3^+$ cells) in tumor at day 6. n= 5 biologically independent samples. Data in **B**, **C** and **D** expressed as the mean ± SEM. P values determined by one-way ANOVA with Tukey's post-hoc test (**B**, **C** and **D**).

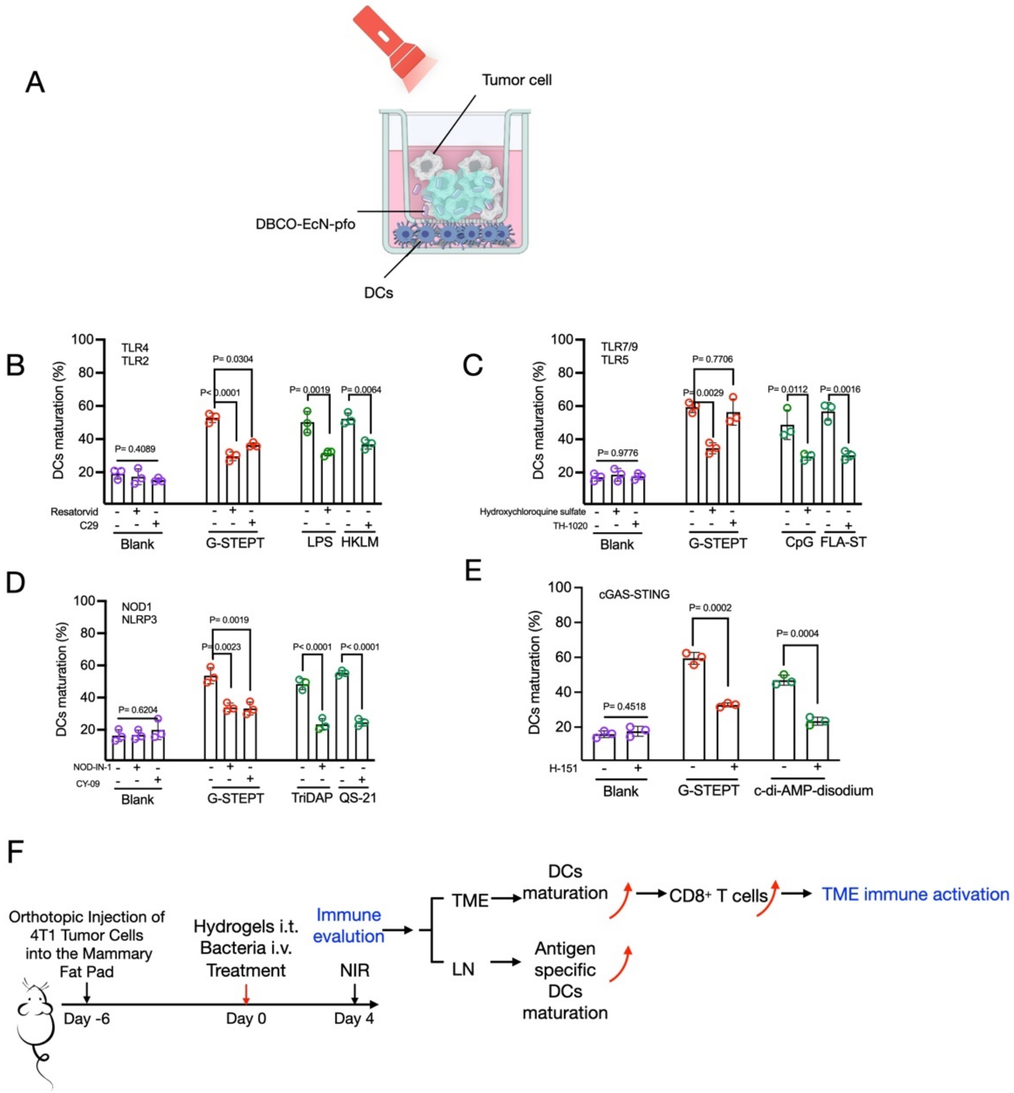

Figure S41.

The signal pathway of DCs maturation induced by STEPT system was analyzed. (**A**) Schematic diagram of the transwell experiment analyzing the maturation of DCs under different conditions. (**B** to **E**) Pathway inhibitors were used to analyze TLR2, TLR4, TLR5, TLR7/9, NOD1, NLRP3 and cGAS-STING pathways *in vitro*. (**B**) The effect of TLR4 inhibitor Resatorvid on DCs cell maturity ($CD11c^+$ MHC II^+ $CD80^+$ $CD86^+$ DCs gated on $CD45^+$ $CD3^-$ cells); The effect of TLR2 inhibitor C29 on DCs cell maturity; DCs cells were treated with LPS and HKLM as activators of TLR4 and TLR2 pathways, respectively, as positive controls. (**C**) The effect of TLR7/9 inhibitor Hydroxychloroquine sulfate on DCs

cell maturity; The effect of TLR5 inhibitor TH-1020 on DCs cell maturity; DCs cells were treated with CpG and FLA-ST as activators of TLR7/9 and TLR5 pathways, respectively, as positive controls. (**D**) The effect of NOD 1 inhibitor NOD-IN-1 on DCs cell maturity; The effect of NLRP3 inhibitor CY-09 on DCs cell maturity; DCs cells were treated with TriDAP and QS-21 as activators of NOD1 and NLRP3 pathways, respectively, as positive controls. (**E**) The effect of cGAS-STING inhibitor H-151 on DCs cell maturity； DCs cells were treated with c-di-AMP-disodium as the activator of cGAS-STING pathway, as positive controls. (**F**) The experimental design to evaluate the in vivo immune responses triggered by STEPT. LN, lymph node. Data in **B, C D** and **E** expressed as the mean ± SEM. P values determined by unpaired two-tailed Student's t-test (**B, C D** and **E**).

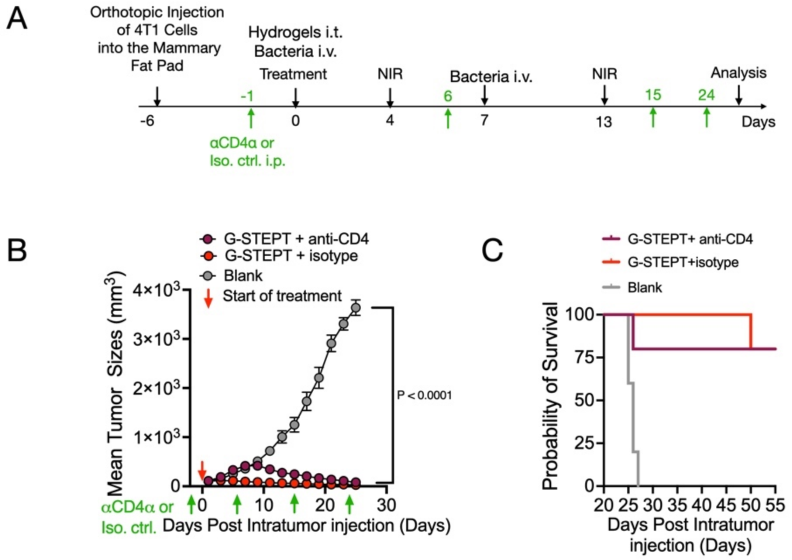

Figure S42.

CD4⁺ T-cell depletion. (**A**) Illustration and timeline of experimental protocols for STEPT therapy and CD4⁺ T-cell depletion in 4T1-luc tumor-bearing mice. (**B**) 4T1-luc tumor growth curves of 4T1-luc tumor-bearing mice after the indicated treatments in **A**; n = 5 biologically independent mice. CD4⁺ T-cell depletion in 4T1-luc tumor-bearing mice. The survival curves (**C**) of 4T1-luc tumor-bearing mice after the indicated treatments; n = 5 biologically independent mice. Data in B and C expressed as the mean ± SEM. P values determined by two-way ANOVA with Bonferroni post-hoc test (**B**), log-rank (Mantel–Cox) test for survival curves (**C**).

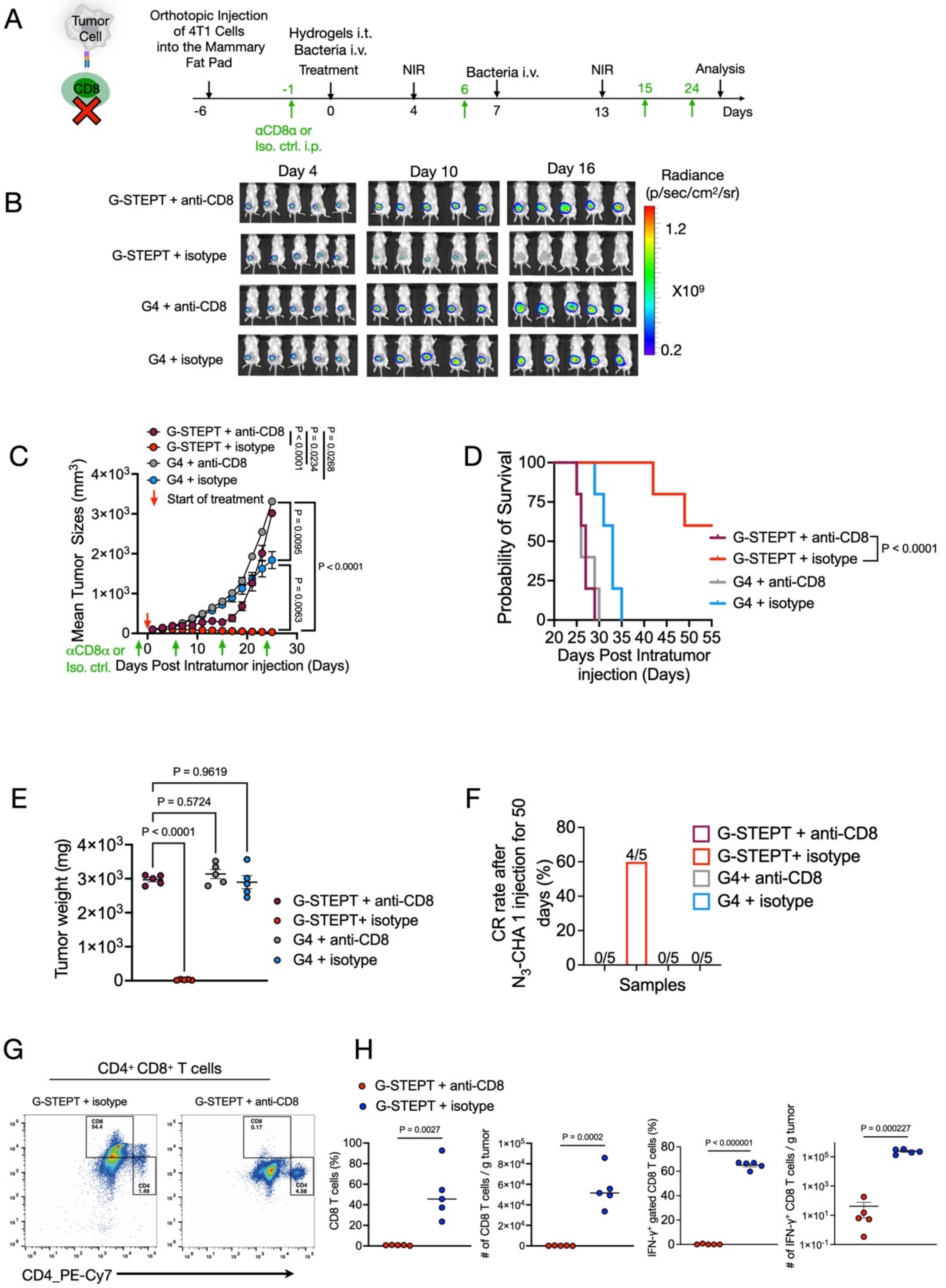

Figure S43.

CD8⁺ T-cell depletion. (**A**) Illustration and timeline of experimental protocols for STEPT therapy and CD8⁺ T-cell depletion in 4T1-luc tumor-bearing mice. (**B**) Representative bioluminescence images of 4T1-luc tumor-bearing mice; n = 5 biologically independent

mice. (**C**) 4T1-luc tumor growth curves of 4T1-luc tumor-bearing mice after the indicated treatments in **A**; n = 5 biologically independent mice. $CD8^+$ T-cell depletion in 4T1-luc tumor-bearing mice. The survival curves (**D**), tumor weight measurements (**E**) and CR rates (**F**) of 4T1-luc tumor-bearing mice after the indicated treatments; n = 5 biologically independent mice. (**G**) Representative flow cytometry histograms and quantitative analysis of endogenous $CD4^+$ and $CD8^+$ T cells (gated on $CD45^+$ $CD3^+$ cells) in tumors at 25 days after the indicated treatment; (**H**) Representative flow cytometry quantitative analysis of endogenous $CD8^+$ T cells (gated on $CD45^+$ $CD3^+$ cells) (left panel; n = 5 biologically independent samples) and intracellular IFN-γ^+ $CD8^+$ T cells (gated on $CD45^+$ $CD3^+$ $CD8^+$ cells) (right panel; n = 5 biologically independent samples) in tumors at 25 days after the indicated treatment. n = 5 biologically independent samples. Data in **C**, **D**, **E** and **H** expressed as the mean ± SEM. P values determined by two-way ANOVA with Bonferroni post-hoc test (**C**); log-rank (Mantel–Cox) test for survival curves (**D**); one-way ANOVA with Tukey's post-hoc test (**E**); and unpaired two-tailed Student's t-test (**H**).

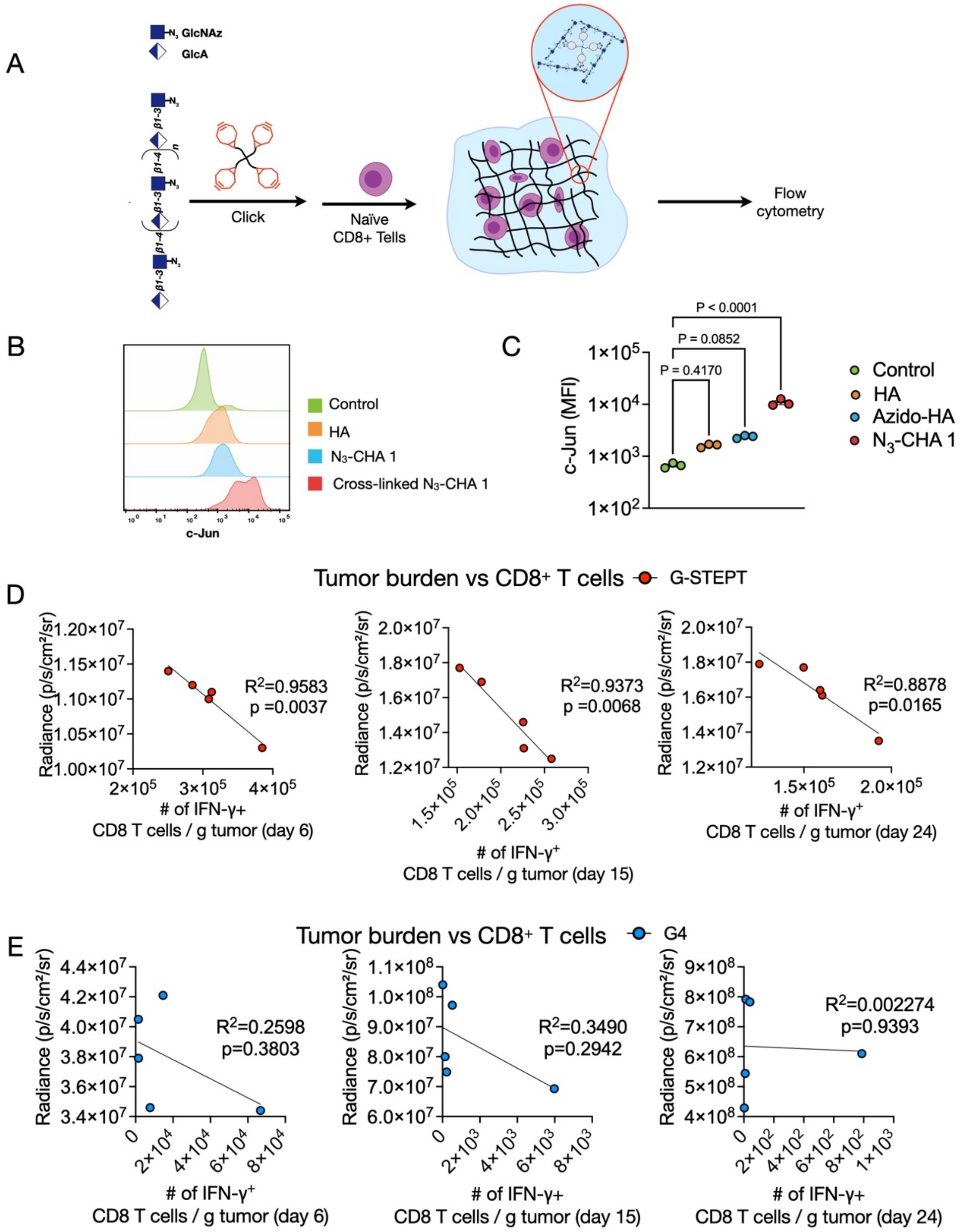

Figure S44.

(**A**) Illustration of the simultaneous addition of azido-HA with crosslinking agent PEG-tBCN to T-cell cultures and incubation at room temperature for 20 min; the solution was removed, and the cells were incubated at 37 °C for 1 h then subjected to flow cytometry analysis. (**B** and **C**) Flow cytometry analysis of c-jun⁺ CD8⁺ T cells; n = 3 biologically

independent samples. (**D** and **E**) Frequency of IFN-γ$^+$ CD8$^+$ T cells versus tumor radiance under STEPT treatment (**D**) or control treatment (**E**). Data in **C, D** and **E** expressed as the mean ± SEM. P values determined by one-way ANOVA with Tukey's post-hoc test (**C**) and simple linear regression (**D** and **E**).

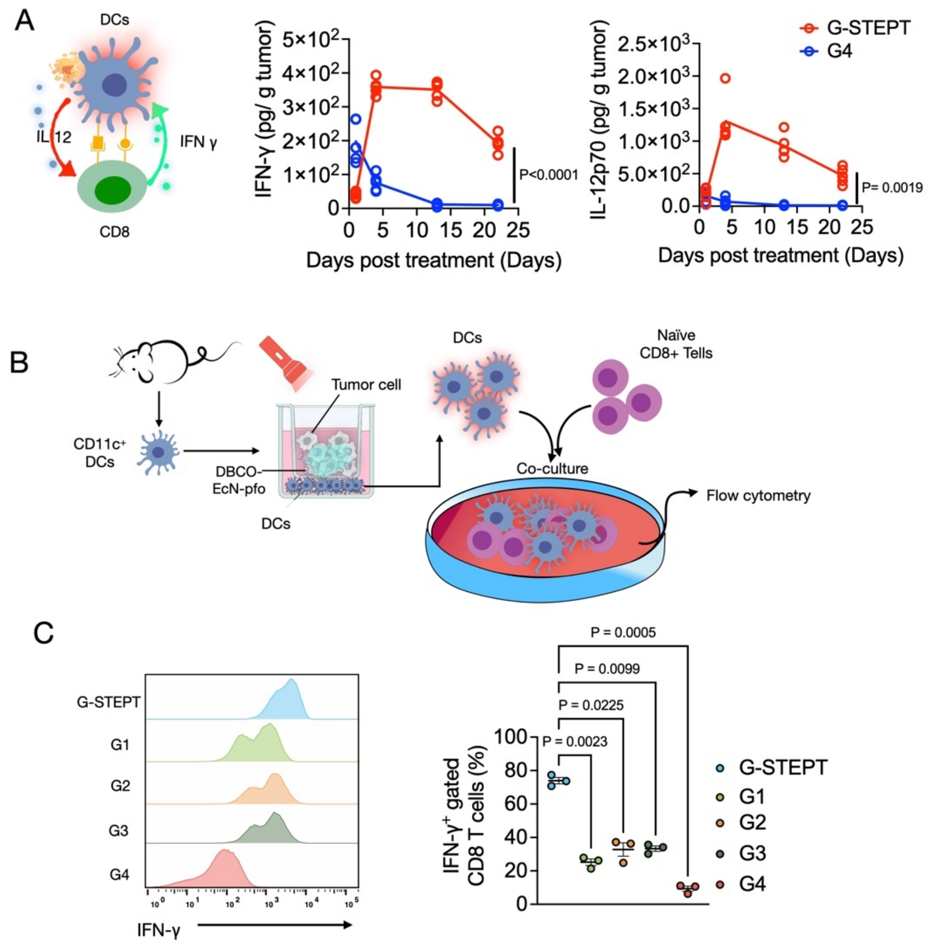

Figure S45.

(**A**) Illustration of the positive feedback signal of IL-12, which is secreted by DCs to activate T cells that then produce IFN-γ to trigger further production of IL-12 by DCs, thereby enhancing T-cell-derived IFN-γ expression and cytotoxic activity during STEPT treatment (left panel); Intratumor cytokine levels of IFN-γ and IL-12p70 at day 25 in the breast tumor-bearing model mice (right panel); n = 5 biologically independent samples. (**B** and **C**) Schematic diagram of STEPT therapy-mediated activation of T cells through DC antigen presentation *in vitro*. Briefly, BMDCs were collected from mice, from which CD11c$^+$ cells and T cells were each enriched. Cultured DCs were cultured in the lower transwell chamber, and a mixture of azido-HA hydrogel, 4T1 cells, and logarithmically growing DBCO-EcN-

pfo were added to the upper chamber, which was irradiated by NIR. DCs from the lower chamber were removed and incubated with naive $CD8^+$ T cells, then stained with antibodies ($CD45^+$, $CD3^+$, $CD8^+$, IFN-γ^+) and analyzed by flow cytometry. (**C**) Quantitative analysis of intracellular IFN-γ^+ $CD8^+$ T cells (gated on $CD45^+$ $CD3^+$ $CD8^+$ cells) *in vitro*; n = 3 biologically independent samples. Data in **A** and **C** expressed as the mean ± SEM. P values determined by two-way ANOVA with Bonferroni post-hoc test (**A**) and one-way ANOVA with Tukey's post-hoc test (**C**).

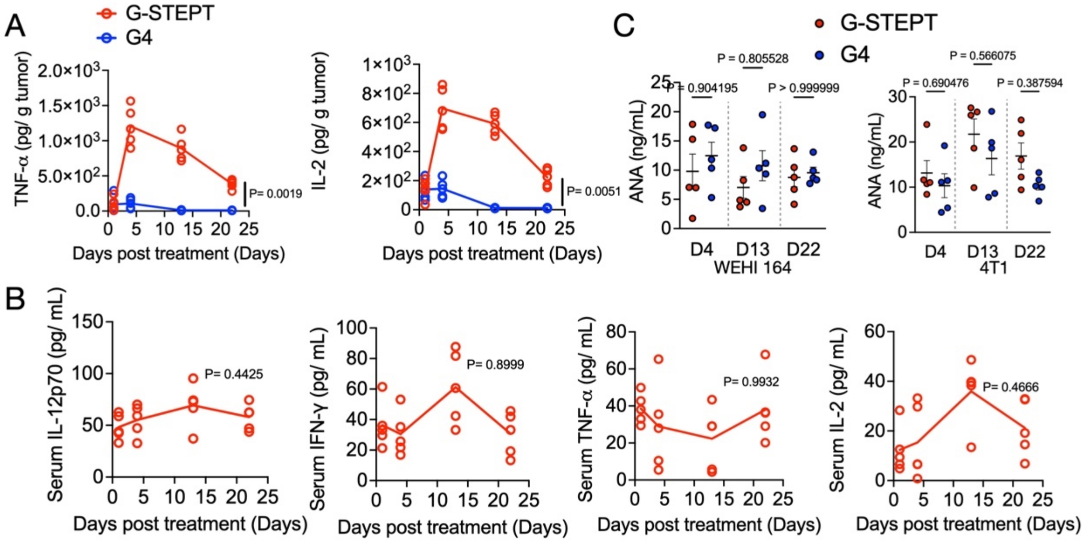

Figure S46.

(**A**) Time curve of concentrations of cytokines TNF-α and IL-2 secreted by infiltrating immune cells at the tumor site after STEPT and G4 treatment; n = 5 biologically independent mice. (**B**) Time course of serum cytokine levels after STEPT treatment in the 4T1 tumor model; n = 5 biologically independent mice. (**C**) Serum ANA levels in fibrosarcoma (left panel) and breast tumor (right panel) models at days 4, 13, and 22 post STEPT treatment; n = 5 biologically independent mice. Data in **A** to **C** expressed as the mean ± SEM. P values determined using two-way ANOVA with Bonferroni post-hoc test (**A**), or unpaired two-tailed Student's t-test (**C**).

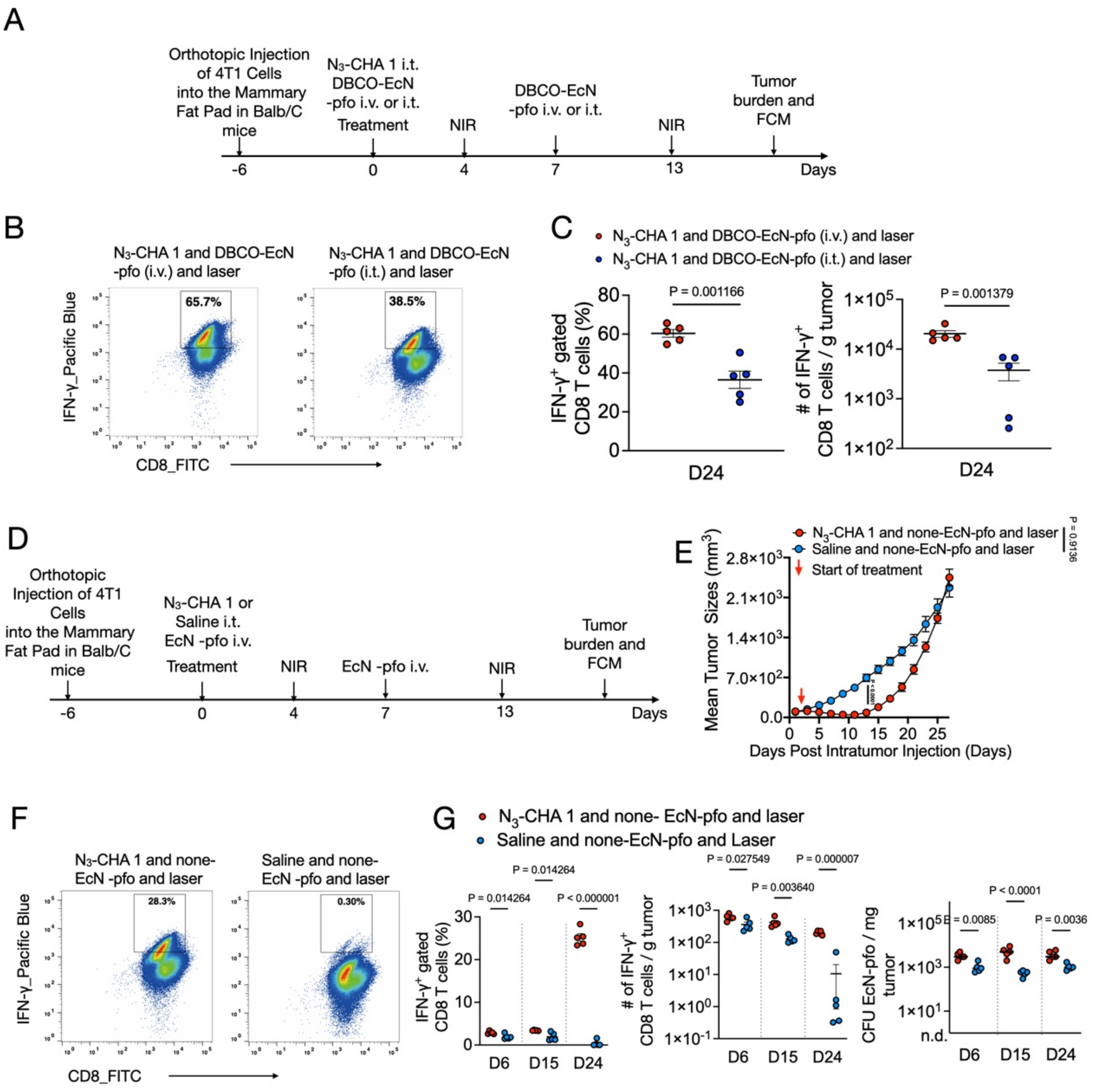

Figure S47.

Necessity of both DBCO-EcN-pfo and azido-HA for effective STEPT therapy. We tested intratumor injection of DBCO-EcN-pfo and found that it did not produce the same effect. (**A**) Illustration of the experimental protocols for intravenous or intratumor bacterial therapy in 4T1-luc tumor-bearing mice. (**B**) Flow cytometry histograms at day 24. The level of tumor infiltration of IFN-γ$^+$ CD8$^+$ T cells was much lower in the intratumor injection group than in the intravenous administration group at day 24. Therefore, we speculated that EcN was better mobilized by the immune system after entering the bloodstream. The prolonged half-life of intravenously injected EcN made it easier to locate N_3-CHA 1 in tumors, improving the targeting, and the introduction of EcN into the blood enhanced aerobic proliferation to some extent. (**C**) The quantitative analysis of intracellular IFN-

γ^{+}CD8^{+} T cells (gated on CD45^{+} CD3^{+} CD8^{+} cells) in the tumors at day 24 after the indicated treatment of 4T1-luc tumor-bearing mice. n = 5 biologically independent mice. (**D**) Illustration of the experimental protocols for STEPT therapy with and without azido-HA in 4T1-luc tumor-bearing mice. (**E**) 4T1-luc tumor growth curves after the indicated treatment; n = 5 biologically independent mice. (**F** and **G**) Flow cytometry histograms of intracellular IFN-γ^{+} CD8^{+} T cells (gated on CD45^{+} CD3^{+} CD8^{+} cells) in tumors at day 24 and quantitative analysis of intracellular IFN-γ^{+} CD8^{+} T cells (gated on CD45^{+} CD3^{+} CD8^{+} cells) in tumors at days 6, 15, and 24 after the indicated treatment. There was also an expansion of infiltrating IFN-γ^{+} CD8^{+} T cells within the tumor in the N_3-CHA 1 group, which was consistent with the enhancement of T-cell activity by N_3-CHA 1 hydrogel *in vitro*. n = 5 biologically independent samples. Calculation of EcN CFUs in tumors at different time points. Data in **C**, **E**, and **G** expressed as the mean ± SEM. P values determined using two-way ANOVA with Bonferroni post-hoc test (**E**), or unpaired two-tailed Student's t-test (**C** and **G**). FCM, Flow cytometry.

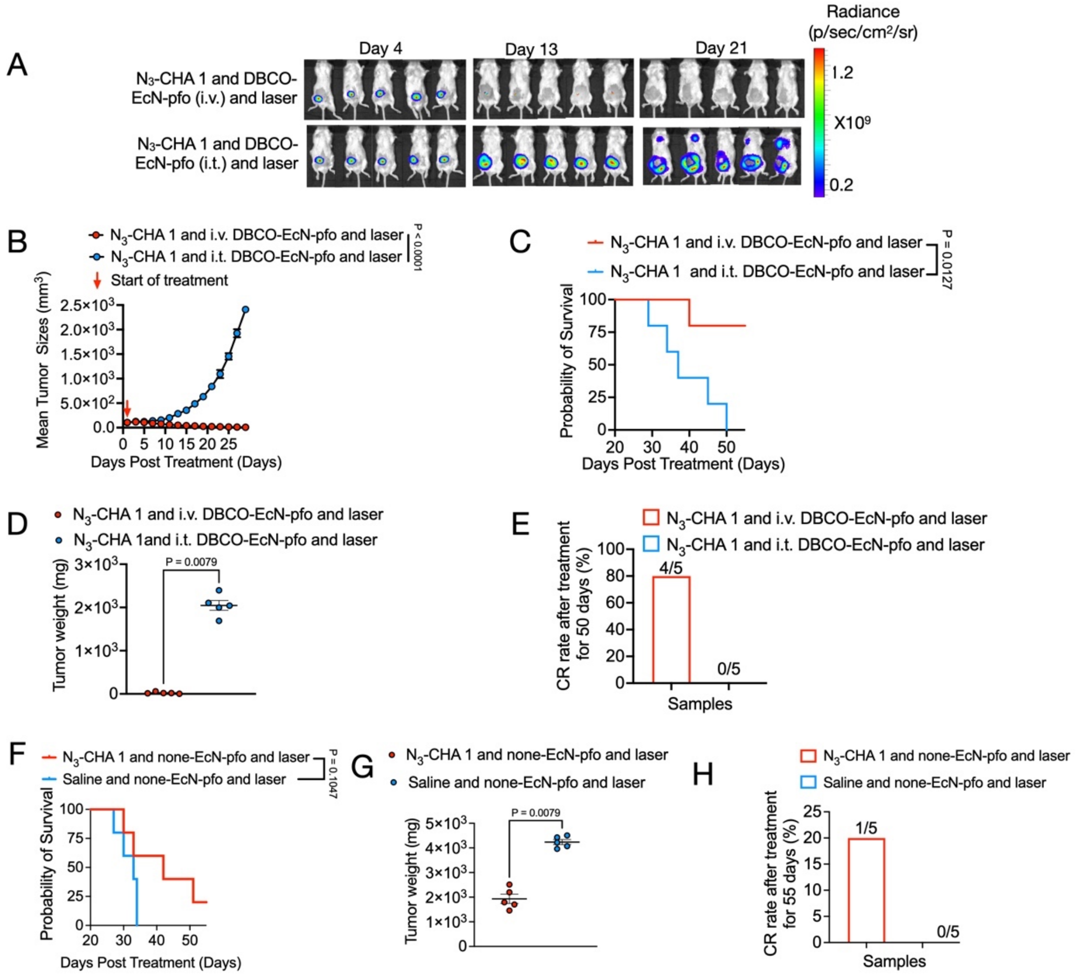

Figure S48.

(**A**) Representative IVIS bioluminescence images of 4T1-luc tumor-bearing mice. See Figure S46A for treatment details. (**B**) Tumor growth curves of 4T1-luc tumor-bearing mice after the indicated treatments. (**C**) Survival curves, (**D**) tumor weight measurements, (**E**) completed reaction rates; n = 5 biologically independent mice in each experiment. (**F** to **H**) STEPT therapy with and without azido-HA in 4T1-luc tumor-bearing mice, see Figure S46D for treatment details. (**F**) Survival curves, (**G**) tumor weight measurements, (**H**) completed reaction rates; n = 5 biologically independent mice in each experiment. Data in **B**, **C**, **D**, **F**, and **G** expressed as the mean ± SEM. P values determined by unpaired two-

tailed Student's t-test (**D**, **G**), two-way ANOVA with Bonferroni post-hoc test (**B**), or log-rank (Mantel–Cox) test for survival curves (**C**, **F**).

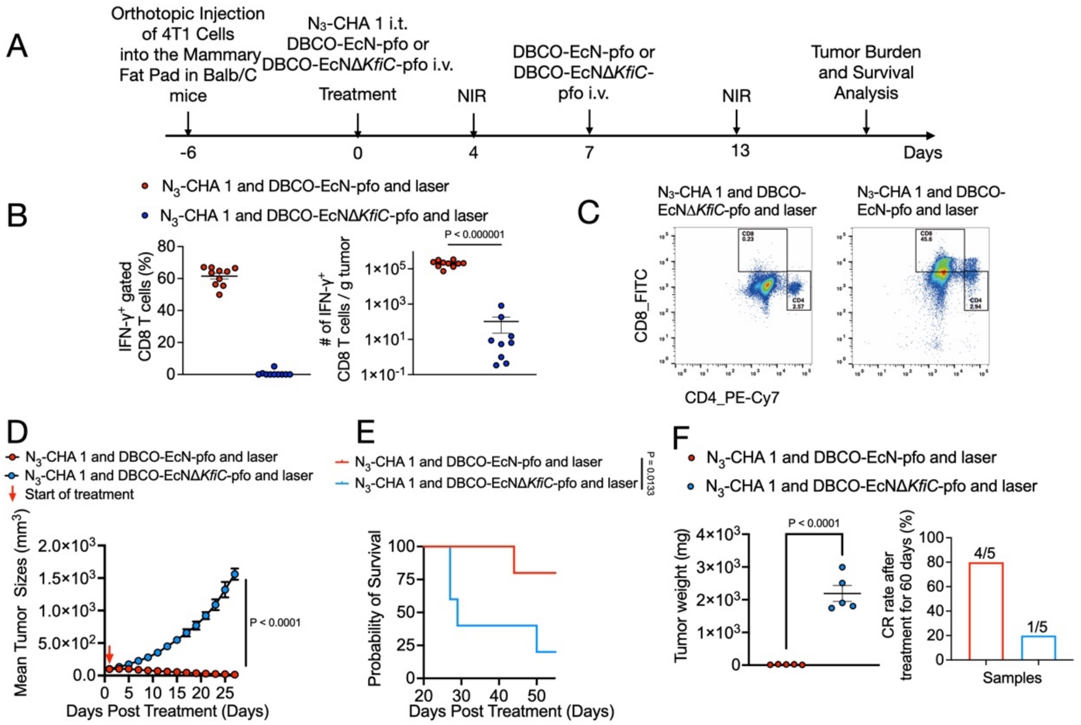

Figure S49.

(**A**) Illustration of experimental protocols for STEPT therapy with DBCO-EcN-pfo or DBCO-EcN Δ *KfiC*-pfo in 4T1-luc tumor-bearing mice. (**B**) Quantitative analysis of intracellular IFN-γ+ CD8+ T cells (gated on CD45+ CD3+ CD8+ cells) and (**C**) endogenous CD4+ and CD8+ T cells (gated on CD45+ CD3+ cells) in tumors at 14 days after the indicated treatment; n = 10 biologically independent samples. (**D**) Tumor growth curves, (**E**) survival curves, and (**F**) tumor weight measurements and CR rates after the indicated treatment in 4T1-luc tumor-bearing mice; n = 5 biologically independent mice. Data in **B**, **D**, **E** and **F** expressed as the mean ± SEM. P values determined by unpaired two-tailed Student's t-test (**B**, **F**), two-way ANOVA with Bonferroni post-hoc test (**D**), and log-rank (Mantel–Cox) test for survival curves (**E**).

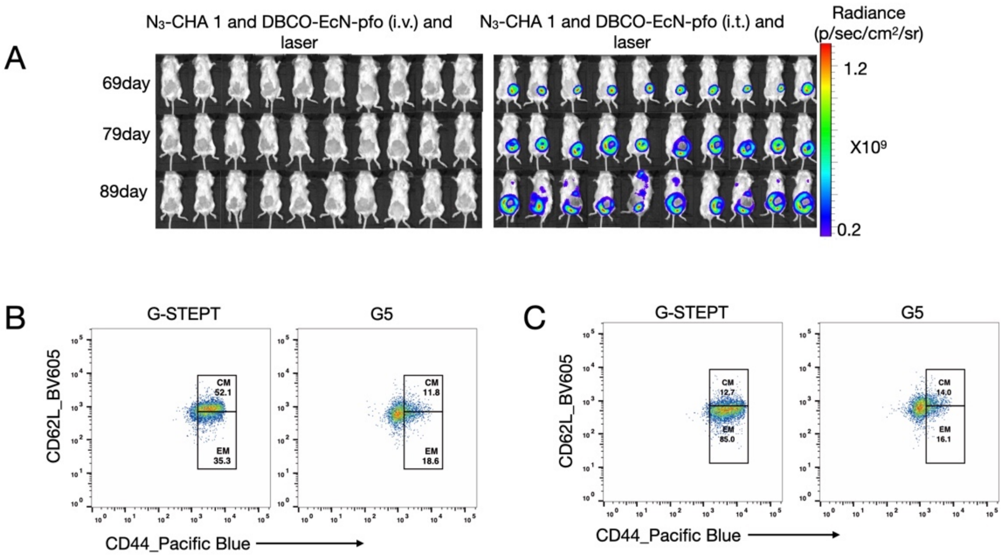

Figure S50.

(**A**) Whole Bioluminescence images of Figure 7D. (**B**) Illustration and representative flow cytometry histograms of memory $CD8^+$ T cells (gated on $CD45^+$ $CD3^+$ $CD8^+$ cells) at day 93. Effector memory (EM) T cells ($CD3^+$ $CD8^+$ $CD44^+$ $CD62L^-$) and central memory (CM) T cells ($CD3^+$ $CD8^+$ $CD44^+$ $CD62L^+$) of Figure 7G. (**C**) Representative flow cytometry histograms of memory $CD8^+$T cells (gated on $CD45^+$ $CD3^+$ $CD8^+$ cells) of Figure 7N at day 20. Effector memory (EM) T cells ($CD3^+$ $CD8^+$ $CD44^+$ $CD62L^-$) and central memory (CM) T cells ($CD3^+$ $CD8^+$ $CD44^+$ $CD62L^+$).

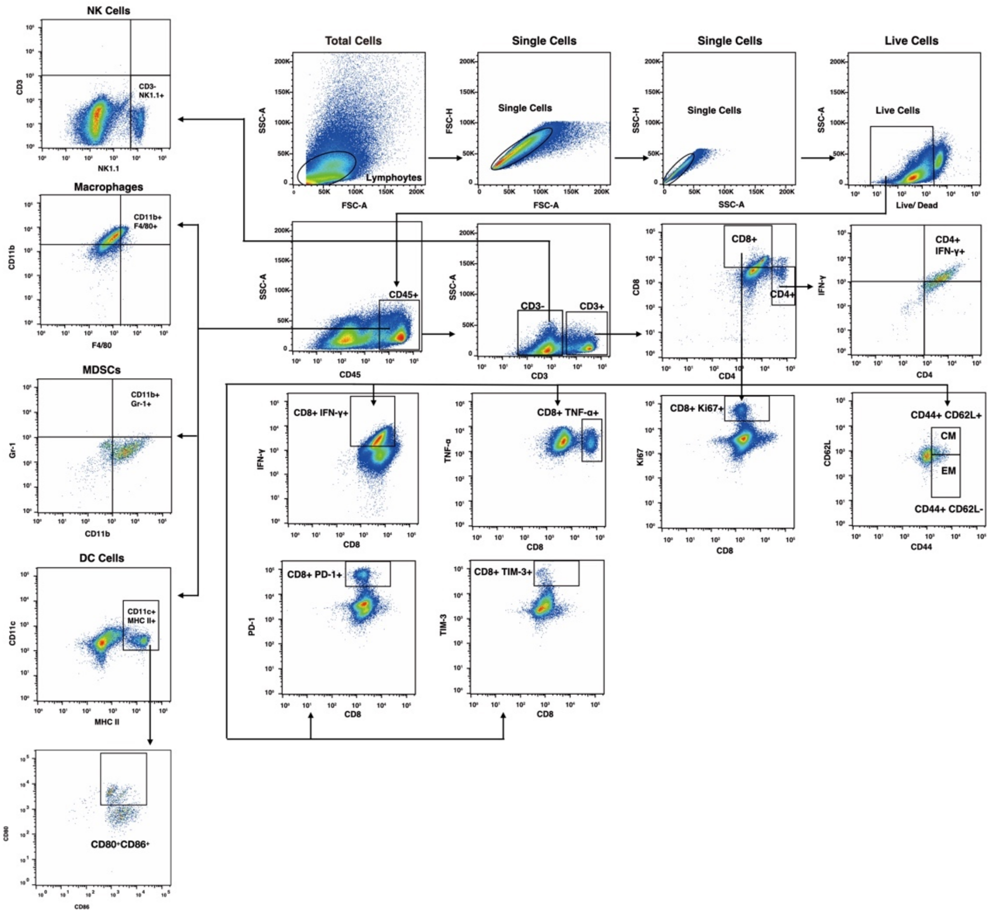

Figure S51.

Gating strategies for flow cytometry analyses.

T-cell gating strategies were as follows: lymphocytes (forward scatter [FSC]-height [H] vs. side scatter [SSC]-H); single cells (FSC-H vs. FSC-area [A]); live CD45$^+$ cells (Zombie yellow$^-$ and CD45$^+$); T cells (CD3$^+$); CD8 T cells (CD8α$^+$, CD4$^-$gated on CD3$^+$ cells); CD4$^+$ T cells (CD8α$^-$, CD4$^+$ gated on CD3$^+$ cells); Th1 cells (IFN-γ $^+$ gated on CD4$^+$ T cells); Tc1 cells (IFN-γ$^+$ gated on CD8$^+$ T cells); proliferating Tc1 cells (Ki67$^+$ gated on Tc1 cells); live CD45$^+$ cells (Zombie yellow$^-$ and CD45$^+$); DCs (CD80$^+$, CD86$^+$, MHC-II$^+$, CD11c$^+$ in living CD45$^+$ cells); macrophages (CD11b$^+$, F4/80$^+$ on living CD45$^+$ cells; myeloid-derived suppressor cells (CD11b$^+$, Gr-1$^+$ in living CD45$^+$ cells); and natural killer cells (NK1.1$^+$, CD3$^-$ on living CD45$^+$, CD8α$^-$ and CD4$^-$ cells). Innate immune cell gating strategies: lymphocytes (FSC-H vs. SSC-H), single cells (FSC-H vs. FSC-A).

Table S1. Bacterial strains and plasmids

Strain or plasmid	Characteristics	Source
***Escherichia coli* strains**		
DH5α		Thermo Fisher
BL21 (DE3)		Novagen
Nissle 1917 (EcN)	*E. coli* DSM 6601, gentamicin resistance	DSMZ Braunschweig
EcNΔ*KfiC*	WT derivate, *KfiC* deleted	This work
EcNΔ*KfiC*-GFP	WT derivate, *KfiC* deleted, ampicillin resistance and carrying pGEN-*GFP*	This work
EcN-GFP	WT derivate, ampicillin resistance and carrying pGEN-*GFP*	This work
EcN-P_{consis}-mCherry	WT derivate, ampicillin resistance and carrying pGEN-*mCherry* and pBV220-BRP-*phoA*-pCadC-Lpp-ompA-S_{MMP}-*pfo*-HA-Axe-Txe	This work
EcN-luxCDABE	WT derivate, ampicillin resistance and carrying pGEN-*luxCDABE*	This work
EcN-mCherry	WT derivate, ampicillin resistance and carrying pUC57-pCadC-*mcherry*	This work
EcN-pfo	WT derivate, ampicillin resistance and carrying pBV220-BRP-*phoA*-pCadC-Lpp-ompA-S_{MMP}-*pfo*-Axe-Txe	This work
EcN-pfo-HA	WT derivate, ampicillin resistance and carrying pBV220-BRP-*phoA*-pCadC-Lpp-ompA-S_{MMP}-*pfo*-HA-Axe-Txe	This work
EcNΔ*KfiC*-pfo	WT derivate, *KfiC* deleted, ampicillin resistance and carrying pBV220-BRP-*phoA*-pCadC-Lpp-ompA-S_{MMP}-*pfo*-HA-Axe-Txe	This work

EcN-pfo (control, no cleavable MMPs target)	WT derivate, ampicillin resistance and carrying pBV220-BRP-*phoA*-pCadC-Lpp-ompA-*pfo*-Axe-Txe	This work
EcN-pfo (no BRP)	WT derivate, ampicillin resistance and carrying pBV220-*phoA*-pCadC-Lpp-ompA-S_{MMP}-*pfo*-Axe-Txe	This work
EcN-pR-pL-mCherry	WT derivate, ampicillin resistance and carrying pBV220-pR-pL-*mCherry*	This work
EcN-pfo-mCherry	WT derivate, ampicillin resistance and carrying pGEN-*mCherry* and pET28a-POXB20-Lpp-ompA-S_{MMP}-*pfo*-HA	This work
***Bacillus subtilis* strains**		
*BS*168	*Bacillus subtilis* (Ehrenberg) Cohn (ATCC 23857，*Bacillus subtilis 168*)	
*BS*168S (*glmS*Δ-168)	WT derivate, *glmS* deleted，neomycin resistance	This work
*BS*168SS (*glmS*Δ-*NahK*-*AGX1*-168)	WT derivate, *glmS* deleted, kanamycin resistance, genome carrying *NahK* and *AGX1*	This work
*BS*168SSHA (*glmS*Δ-*NahK*-*AGX1*-*szHasA*-168)	WT derivate, *glmS* deleted，kanamycin resistance and chloramphenicol resistance, genome carrying *NahK* and *AGX1*, carrying pHT43-PxylA- *szHasA*	This work
**Plasmids**		
pET28a-Ptrc-*HepIII*	Ptrc promoter, kanamycin resistance, expressing HepIII	This work
pET28a-Ptrc-*AsChnAC*	Ptrc promoter, kanamycin resistance, expressing *AsChnAC*	This work

pHT43	*E. coli*– *Bacillus subtilis* shuttle expression vector, chloramphenicol resistance in *Bacillus subtilis*, ampicillin resistance in *E. coli*	This work
pHT43-*KfiA*	*E. coli*– *Bacillus subtilis* shuttle expression vector, Pgrac promoter, chloramphenicol resistance in *Bacillus subtilis*, ampicillin resistance in *E. coli*	This work
pUC57-*neo*	The plasmid contains the homologous recombination fragment of the gene *glmS*, ampicillin resistance in *E. coli*	This work
pUC57-Pveg-*AGX1*-Pveg-*NahK*	The plasmid contains the homologous recombination fragment of the neo resistance gene, Pveg promoter, two multiple cloning sites, carrying N-acetylhexosamine 1-kinase gene of *bifidobacterium longum*, *NahK* and UDP-N-acetylhexamine pyrophosphate enzyme gene of human, *AGX1*, ampicillin resistance in *E. coli*	This work
pHT43-PxylA-*szHasA*	pHT43 plasmid expressing the HA synthase type I HASs from *Streptococcus zooepidemicus*, szHasA	This work
pGEN-*luxCDABE*	The plasmid contains the bioluminescence gene, *luxCDABE,* ampicillin resistance in *E. coli*	This work
pGEN-*mCherry*	The plasmid contains the red fluorescence protein gene, *mCherry,* ampicillin resistance in *E. coli*	This work
pGEN-*GFP*	The plasmid contains the green fluorescence protein gene, *GFP,* ampicillin resistance in *E. coli*	This work

pBV220-*mcherry*	The plasmid contains the red fluorescence protein gene, *mCherry,* pR-pL, temperature sensitive promoter, ampicillin resistance in *E. coli*	This work
pUC57-pCadC-*mcherry*	The plasmid contains the red fluorescence protein gene, *mCherry,* pCadC, acid sensitive promoter, ampicillin resistance in *E. coli*	This work
pET28a-POXB20-Lpp-ompA-S_{MMP}-*pfo*-HA	The plasmid contains POXB20 promoter, outer membrane protein perfringolysin O with metallomatrix proteinase cleavage site, Lpp-ompA-S_{MMP}-*pfo*, toxin and antitoxin system, Axe-Txe, kanamycin resistance in *E. coli*	This work
pBV220-BRP-*phoA*-pCadC-Lpp-ompA-S_{MMP}-*pfo*-Axe-Txe	The plasmid contains pR-pL, temperature sensitive promoter, the alkaline phosphatase gene, *phoA,* Bacterial release protein, BRP, pCadC, acid sensitive promoter, outer membrane protein perfringolysin O with metallomatrix proteinase cleavage site, Lpp-ompA-S_{MMP}-*pfo*, toxin and antitoxin system, Axe-Txe, ampicillin resistance in *E. coli*	This work
pBV220-BRP-*phoA*-pCadC-Lpp-ompA-S_{MMP}-*pfo*-HA-Axe-Txe	The plasmid contains pR-pL, temperature sensitive promoter, the alkaline phosphatase gene, *phoA,* Bacterial release protein, BRP, pCadC, acid sensitive promoter, outer membrane protein perfringolysin O with metallomatrix proteinase cleavage site and HA tag in C end, Lpp-ompA-S_{MMP}-*pfo*-HA, toxin and antitoxin system, Axe-Txe, ampicillin resistance in *E. coli*	This work

pBV220-*phoA*-pCadC-Lpp-ompA-S_{MMP}-*pfo*-Axe-Txe	The plasmid contains pR-pL, temperature sensitive promoter, the alkaline phosphatase gene, *phoA,* pCadC, acid sensitive promoter, gene of outer membrane protein perfringolysin O with metallomatrix proteinase cleavage site, Lpp-ompA-S_{MMP}-*pfo*, toxin and antitoxin system, Axe-Txe, ampicillin resistance in *E. coli*	This work
pBV220-BRP-*phoA*-pCadC-Lpp-ompA-*pfo*-Axe-Txe	The plasmid contains pR-pL, temperature sensitive promoter, the alkaline phosphatase gene, *phoA,* Bacterial release protein, BRP, pCadC, acid sensitive promoter, outer membrane protein perfringolysin O without metallomatrix proteinase cleavage site, Lpp-ompA- -*pfo*, toxin and antitoxin system, Axe-Txe, ampicillin resistance in *E. coli*	This work

Table S2. Primer sequences used in PCR

Primer	Sequence (5′ to 3′)
glmS_F (*Bacillus subtilis*)	CGATAACGACAACAGAGAAAC
glmS_R (*Bacillus subtilis*)	CTGGATACAGTCAGGTATTCACG
neo_F	TGAGAATAGTGAATGGACCAATAATAATG
neo_R	AACCTGATTGACCGATCTAATGAG
AGX1_F	TCACAGGGAAGCAACATCAAC
AGX1_R	TGGACGCCGTTTTCATCGAT
NahK_2F	GCATTGCTTCACATTTTGCCCTTG
NahK_2R	CAATTGCATGTGTTTCGCTCGC
tuaD_F	TGACTTGTATGAAACCGTGCA
tuaD_R	TCAATATGGGTTCGATTCCG
szHasA _F	ATGAGAACATTAAAAAACCTCATAACTGTTGTGGCC
szHasA _F R	TTATAATAATTTTTTACGTGTTCCCCAGTCAGCATTTC
phoA-F	AAACAGCTATGACCATGATTACGGATTCACT
phoA-R	CAAGCTTGGCTGCAGGTCGACTTATTTCAGC
CadC F	TGATCGAGTGTAGCCAGATCTTTGGTAACTCCGGGTTG
CadC R	ACCAGTTTAGTAGCTTTCATTGCTCTAAGCTCAATT
pfo F	GCTTAGAGCAATGAAAGCTACTAAACTGGTACTGG
pfo R	GTGTCGAGTGGATGGTAGGATCGATTAATTGTAAGTA

axetxe F	GAAGATCATCTTATTAATCAGATAAAATAGGGTTATTTCG ATGAAAGG
axetxe R	GAGTAAACTTGGTCTGACAGCCGCTAATTAACTAAAACT AGTACA
pLpR mCherry F	ATAACGAGGCGCAAAAAATGGTGAGCAAGGGC
pLpR mCherry R	CAAGCTTGGCTGCAGGTCGACCTACTTGTGTACAG
CadC-mCherry R	GTGTCGAGTGGATGGTAGGATCGACTACTTGTACAG
luxCDABE F	AATTCAGGCTTGGAGGATACGTATGACTAAAAAAATT
luxCDABE R	GCATGCCTGCAGGTCATCAACTATCAAACGCTTC
KfiC_sgRNA	TAGAGCTAGAAATAGCAAGTTAAAATAAGGCTAGTCCGT TATCAACTTGAAAAAGTGGCACCGAGTCGGTGC

Table S3. Rheological trace data of different hydrogel formulations

Frequency (Hz)	N_3-CHA 2 G'	N_3-CHA 2 G''	N_3-CHA 4 G'	N_3-CHA 4 G''	N_3-CHA 3 G'	N_3-CHA 3 G''	N_3-CHA 1 G'	N_3-CHA 1 G''
0.1	0.14175	0.50738	91.084	96.095	0.28804	0.91453	1.9788	3.9186
0.127	0.19995	0.62129	107.15	105.1	0.34576	1.187	2.4397	4.6083
0.161	0.25132	0.73668	124.36	111.65	0.49503	1.3916	3.0942	5.2905
0.204	0.31306	0.90521	142.89	118.51	0.66819	1.6527	3.7962	6.1169
0.259	0.38804	1.1387	161.99	125.22	0.82512	2.0174	4.68	6.9836
0.329	0.54758	1.3527	183.28	131.22	1.075	2.3528	5.668	7.9194
0.418	0.69254	1.6131	205.18	136.52	1.3982	2.7223	7.0256	8.8018
0.53	0.83269	1.8702	228.38	141.68	1.7623	3.2079	8.2541	9.6904
0.672	1.0896	2.1689	252.14	142.42	2.1864	3.6601	9.7244	10.859
0.853	1.3404	2.6072	274.17	145.94	2.7193	4.1957	11.546	12.226
1.08	1.7517	3.0272	295.21	149.9	3.2876	4.8319	13.515	12.777
1.37	2.0855	3.4646	322.77	152.89	4.0176	5.5056	15.257	13.949
1.74	2.5683	4.0044	347.46	154.44	4.9498	5.9261	16.912	15.196
2.21	3.1895	4.5645	371.72	155.55	5.9812	6.7444	19.512	16.346
2.81	3.9266	5.1193	396.15	155.97	7.1362	7.5725	22.321	17.347
3.56	4.7665	5.8216	420.91	156.72	8.4417	8.0398	24.863	18.596
4.52	6.2096	6.8055	445.63	156.87	10.081	8.7864	27.697	19.867
5.74	6.7549	7.4172	470.03	156.41	12.399	9.7086	30.985	20.752
7.28	8.26	7.0238	494.14	156.23	15.665	10.624	34.472	22.08
9.24	9.4076	7.4495	518.77	155.61	18.337	11.313	38.483	23.688
11.7	11.258	7.6308	542.73	154.7	23.185	11.561	42.367	24.532
14.9	13.71	10.094	567.22	154.34	29.578	13.066	47.608	26.241
18.9	18.303	9.3892	592.47	151.45	44.03	11.275	52.354	26.255
24	21.278	8.9565	616.2	147.13	59.57	2.8856	59.942	19.847
30.4	27.893	2.7233	644.48	141.53	81.667	6.4012	61.4	29.076

38.6	37.084	11.279	671.83	132.88	118.94	8.9194	75.627	18.682
48.9	38.778	39.341	684.2	103.27	163.34	41.449	83.32	14.293
62.1	51.831	45.516	710.26	43.983	227.46	104.64	83.678	6.5835
78.8	15.493	182.7	558.93	48.357	245.46	202.45	108.57	3.706
100	0.0116<br>34	232.68	1352.1	413.07	868.23	1135.2	137.08	13.802

Table S4. Shear viscosity data different hydrogel formulations at the increased shear rate

Shear rate (s^{-1})	N_3-CHA 2	N_3-CHA 1	N_3-CHA 3	N_3-CHA 4
0.00981	536.11	2944.9	773.85	248000
0.0119	632.93	3261.7	943.39	318000
0.0146	761.11	3592.6	1015.8	349000
0.0177	820.88	3781.5	1039.5	363000
0.0211	872.23	4206.2	1093.5	371000
0.0255	934.47	4311.1	1130.2	375000
0.0311	1023.3	4473.4	1136.7	378000
0.0371	998.72	4674.2	1166.3	378000
0.045	1079.4	4699.3	1164.8	377000
0.0542	1120	4834.3	1197.1	375000
0.0655	1148.3	4933	1260.9	371000
0.0791	1159.6	4996	1361	366000
0.0953	1111.4	5043.4	1487.2	359000
0.115	999	5074	1587.8	349000
0.139	993.44	5098.8	1630.8	337000
0.168	964.56	5096.9	1640.1	322000
0.202	968.27	5081.2	1640.6	305000
0.244	965.98	5057.8	1663.8	287000
0.295	953.55	5012.7	1631.7	267000

0.356	941.58	4947.1	1615.9	246000
0.429	902.56	4866.7	1602.4	226000
0.518	881.63	4770.5	1538.3	206000
0.625	853.02	4646.4	1540.7	186000
0.754	834.11	4499.7	1477.4	166000
0.91	798.38	4349	1419.8	148000
1.1	750.66	4201.1	1353.7	132000
1.33	724.22	4054.7	1340.5	117000
1.6	713.81	3919.9	1279.8	102000
1.93	702.32	3757.9	1225.5	89659
2.33	658.95	3560.7	1181.7	78051
2.81	643.73	3384.7	1124.4	68144
3.39	627.38	3189.8	1059.3	59435
4.09	598.05	2990.4	1000.6	51997
4.94	567.33	2792.9	943.17	45524
5.96	533.78	2611	885.71	39753
7.2	505.5	2421.1	823.17	34613
8.69	475.91	2227.3	764.88	30289
10.5	444.92	2049.1	707.62	26247
12.6	416.14	1870.7	650.58	22775
15.3	386.86	1708.6	596.76	19610
18.4	358.62	1558.1	545.15	16921

22.2	331.34	1420.5	495.75	14518
26.8	304.58	1292.4	449.79	12397
32.4	279.07	1163.3	406.81	10513
39.1	254.89	1037.9	366.65	8871
47.1	232.15	931.46	329.47	7459.1
56.9	210.77	830.79	295.21	6295
68.7	190.81	731.65	263.84	5248.1
82.9	172.26	651.5	235.27	4403.2
100	155.15	573.65	209.31	3717.6

Table S5. Antibodies used in the study

Laser	Antibody	Fluorochrome	Supplier	Catalogue n°	Working concentration
n.a.	CD- 16/CD32	n.a	Thermo Fisher	14-0161-82	1:100
	c-jun	n.a	Invitrogen	MA5-15881	1:100
	CD8α	n.a	BioXCell	BP0004-1	1:100
	CD4α	n.a	BioXCell	BP0003-1	1:100
	lgG2a isotype control	n.a	BioXCell	BP0089	1:100
Violet	Ki67	PB	BioLegend	652421	1:100
	CD44	PB	BioLegend	156005	1:100
	IFN-γ	PB	BioLegend	505817	1:100
	CD8α	PB	BioLegend	100728	1:50
	CD80	PB	BioLegend	104723	1:100
	CD62L	BV605	BioLegend	104437	1:100

	TIM-3	BV 421	BioLegend	134019	1:50
	Zombie Yellow™ Fixable Viability Kit	BV570	BioLegend	423104	1:100/1:200
Blue	IFN-γ	FITC	BioLegend	505805	1:50
	CD8α	FITC	BioLegend	100803	1:100
	PD-1	FITC	BioLegend	135213	1:100
	Gr-1	FITC	BioLegend	108405	1:100
	CD11b	FITC	BioLegend	101205	1:100
	CD3	FITC	BioLegend	100203	1:100
	CD11c	FITC	BioLegend	117305	1:100
	Puromycin	AF488	Sigma Aldrich	MABE343-AF488	1:200
YG	CD8α	PE Cy7	BioLegend	100721	1:100
	CD4	PE Cy7	BioLegend	100421	1:100
	IFN-γ	PE Cy7	BioLegend	505826	1:100

	CD11b	PE Cy7	BioLegend	101215	1:100
	F4/80	PE Cy7	BioLegend	123113	1:100
	NK1.1	PE Cy7	BioLegend	156513	1:100
	MHC II	PE Cy7	BD Biosciences	750281	1:100
	OVA	PE Cy7	BioLegend	141607	1:100
	TNF-α	PE Cy7	BioLegend	506323	1:100
	CD86	PE	BioLegend	159203	1:100
RED	CD3	APC	BioLegend	100235	1:100
	CD45	APC	BioLegend	147707	1:100

AF488, Alexa Fluor488; APC, allophycocyanin; BV, Brilliant Violet; FITC, Fluorescein Isothiocyanate; PB, Pacific blue; PE Cy7, Phycoerythrin Cyanine 7; n.a., not applicable.

References

1 Yin, F.-X., Wang, F.-S. & Sheng, J.-Z. Uncovering the Catalytic Direction of Chondroitin AC Exolyase: FROM THE REDUCING END TOWARDS THE NON-REDUCING END*. *Journal of Biological Chemistry* **291**, 4399-4406, doi:https://doi.org/10.1074/jbc.C115.708396 (2016).

2 Moody, C. T., Palvai, S. & Brudno, Y. Click cross-linking improves retention and targeting of refillable alginate depots. *Acta Biomaterialia* **112**, 112-121, doi:https://doi.org/10.1016/j.actbio.2020.05.033 (2020).

3 Gurbatri, C. R. *et al.* Engineering tumor-colonizing E. coli Nissle 1917 for detection and treatment of colorectal neoplasia. *Nature Communications* **15**, 646, doi:10.1038/s41467-024-44776-4 (2024).

4 Shatursky, O. *et al.* The Mechanism of Membrane Insertion for a Cholesterol-Dependent Cytolysin: A Novel Paradigm for Pore-Forming

Toxins. *Cell* **99**, 293-299, doi:https://doi.org/10.1016/S0092-8674(00)81660-8 (1999).
5 Abedi, M. H. *et al.* Ultrasound-controllable engineered bacteria for cancer immunotherapy. *Nature Communications* **13**, 1585, doi:10.1038/s41467-022-29065-2 (2022).
6 Kim, Y. C., Tarr, A. W. & Penfold, C. N. Colicin import into E. coli cells: A model system for insights into the import mechanisms of bacteriocins. *Biochimica et Biophysica Acta (BBA) - Molecular Cell Research* **1843**, 1717-1731, doi:https://doi.org/10.1016/j.bbamcr.2014.04.010 (2014).
7 van der Wal, F. J., Luirink, J. & Oudega, B. Bacteriocin release proteins: mode of action, structure, and biotechnological application. *FEMS Microbiology Reviews* **17**, 381-399, doi:10.1111/j.1574-6976.1995.tb00221.x (1995).
8 Dekker, N., Tommassen, J. & Verheij Hubertus, M. Bacteriocin Release Protein Triggers Dimerization of Outer Membrane Phospholipase A In Vivo. *Journal of Bacteriology* **181**, 3281-3283, doi:10.1128/jb.181.10.3281-3283.1999 (1999).
9 Rodríguez-Silvestre, P. *et al.* Perforin-2 is a pore-forming effector of endocytic escape in cross-presenting dendritic cells. *Science* **380**, 1258-1265, doi:10.1126/science.adg8802 (2023).
10 Fu, A. *et al.* Tumor-resident intracellular microbiota promotes metastatic colonization in breast cancer. *Cell* **185**, 1356-1372.e1326, doi:https://doi.org/10.1016/j.cell.2022.02.027 (2022).
